# Supplementary material for: Performance of CADM1, MAL and miR124-2 methylation as triage markers for early detection of cervical cancer in self-collected and clinician-collected samples: an exploratory observational study in Papua New Guinea
Source: BMJ Open. 2024 Jun 19;14(6):e081282. doi: 10.1136/bmjopen-2023-081282 (PMC11191780; doi:10.1136/bmjopen-2023-081282)
Supplement: Author's manuscript [file bmjopen-2023-081282.draft_revisions.pdf]

BMJ Open

BMJ Open is committed to open peer review. As part of this commitment we make the peer review history of every article we publish publicly available.

When an article is published we post the peer reviewers' comments and the authors' responses online. We also post the versions of the paper that were used during peer review. These are the versions that the peer review comments apply to.

The versions of the paper that follow are the versions that were submitted during the peer review process. They are not the versions of record or the final published versions. They should not be cited or distributed as the published version of this manuscript.

BMJ Open is an open access journal and the full, final, typeset and author-corrected version of record of the manuscript is available on our site with no access controls, subscription charges or pay-per-view fees (http://bmjopen.bmj.com).

If you have any questions on BMJ Open's open peer review process please email info.bmjopen@bmj.com

BMJ Open

Clinical performance of CADM1, MAL and miR124-2 methylation as triage markers for early detection of cervical cancer in self- and clinician-collected samples in Papua New Guinea

Journal:	*BMJ Open*
Manuscript ID	bmjopen-2023-081282
Article Type:	Original research
Date Submitted by the Author:	23-Oct-2023
Complete List of Authors:	Molano, Monica; The Royal Women's Hospital, Centre for Women's Infectious Diseases; Murdoch Children's Research Institute<br>Machalek, Dorothy; University of New South Wales, Kirby Institute; The Royal Women's Hospital, Centre for Women's Infectious Diseases<br>Tan, Grace; Victorian Cytology Service<br>Garland, Suzanne; The Royal Women's Hospital, Microbiology and Infectious Diseases Department; The University of Melbourne, Department of Obstetrics and Gynaecology<br>Balgovind, Prisha; The Royal Women's Hospital; Murdoch Children's Research Institute<br>Haqshenas, Gholamreza; The Royal Women's Hospital; The University of Melbourne, Department of Obstetrics and Gynaecology<br>Munnull, Gloria; Papua New Guinea Institute of Medical Research<br>Phillips, Samuel; The Royal Women's Hospital, Microbiology and Infectious Diseases; Murdoch Children's Research Institute<br>Badman, Steven; University of New South Wales, Kirby Insitute<br>Bolnga, John ; Modilon General Hospital, Obs and Gynae ; Papua New Guinea Institute of Medical Research, Sexual and Reproductive Health<br>Cornall, Alyssa; The Royal Women's Hospital, Molecular Microbiology; Murdoch Children's Research Institute<br>Gabuzzi, Josephine; Papua New Guinea Institute of Medical Research<br>Kombati, Zure; Mount Hagen General Hospital, Tininga Clinic<br>Brotherton, Julia; Victorian Cytology Service; The University of Melbourne, School of Population Global Health<br>Saville, Marion; Victorian Cytology Service; The University of Melbourne, Department of Obstetrics and Gynaecology<br>Hawkes, David; Victorian Cytology Service<br>Kaldor, John; University of New South Wales, The Kirby Institute<br>Toliman, Pamela; University of New South Wales, Kirby Institute -Faculty of Medicine<br>Vallely, Andrew; Papua New Guinea Institute of Medical Research, Sexual and Reproductive Health Unit; University of New South Wales, The Kirby Institute<br>Murray , Gerald L ; The University of Melbourne - Parkville Campus, Obstetrics and Gynaecology; The Royal Women's Hospital, Centre for Women's Infectious Diseases

Keywords:	Molecular diagnostics < INFECTIOUS DISEASES, MOLECULAR BIOLOGY, Oncogenes < ONCOLOGY

SCHOLARONE™
Manuscripts

I, the Submitting Author has the right to grant and does grant on behalf of all authors of the Work (as defined in the below author licence), an exclusive licence and/or a non-exclusive licence for contributions from authors who are: i) UK Crown employees; ii) where BMJ has agreed a CC-BY licence shall apply, and/or iii) in accordance with the terms applicable for US Federal Government officers or employees acting as part of their official duties; on a worldwide, perpetual, irrevocable, royalty-free basis to BMJ Publishing Group Ltd ("BMJ") its licensees and where the relevant Journal is co-owned by BMJ to the co-owners of the Journal, to publish the Work in this journal and any other BMJ products and to exploit all rights, as set out in our licence.

The Submitting Author accepts and understands that any supply made under these terms is made by BMJ to the Submitting Author unless you are acting as an employee on behalf of your employer or a postgraduate student of an affiliated institution which is paying any applicable article publishing charge ("APC") for Open Access articles. Where the Submitting Author wishes to make the Work available on an Open Access basis (and intends to pay the relevant APC), the terms of reuse of such Open Access shall be governed by a Creative Commons licence – details of these licences and which Creative Commons licence will apply to this Work are set out in our licence referred to above.

Other than as permitted in any relevant BMJ Author's Self Archiving Policies, I confirm this Work has not been accepted for publication elsewhere, is not being considered for publication elsewhere and does not duplicate material already published. I confirm all authors consent to publication of this Work and authorise the granting of this licence.

For peer review only

Clinical performance of *CADM1, MAL* and *miR124-2* methylation as triage markers for early detection of cervical cancer in self- and clinician-collected samples in Papua New Guinea

Monica Molano[1,2], Dorothy A Machalek[1,3], Grace Tan[5], Suzanne M Garland[1,2,4], Samuel Phillips[1,2], Prisha Balgovind[1,2], Steven G Badman[3], Gholamreza Haqshenas[1,2,4], John Bolnga[6], Alyssa Cornall[1,2], Josephine Gabuzzi[6], Zure Kombati[7], Gloria Munnull[8], Julia M Brotherton[5], Marion Saville[5], David Hawkes[5], John M Kaldor[3], Pamela J Toliman[3], Andrew J Vallely[3]*, Gerald L. Murray[1,2,4]*

1. Centre for Women's Infectious Diseases, The Royal Women's Hospital, Melbourne, Victoria, Australia

2. Murdoch Children's Research Institute, Melbourne, Vic, Australia

3. The Kirby Institute, University of New South Wales, Sydney, NSW, Australia

4. Department of Obstetrics and Gynaecology, University of Melbourne, Parkville, Victoria, Australia

5. Australian Centre for the Prevention of Cervical Cancer, Melbourne, Victoria, Australia

6. Papua New Guinea Institute of Medical Research, Goroka, Papua New Guinea ; Department of Obstetrics and Gynaecology, Modilon General Hospital, Madang, Papua New Guinea.

7. Department of Pathology, Mt Hagen Provincial Hospital, WHP 281, Papua New Guinea

8. Papua New Guinea Institute of Medical Research, Goroka, Papua New Guinea; Department of Obstetrics and Gynaecology, Mt Hagen Provincial Hospital, Mount Hagen, Papua New 6.

*Indicates co-senior authors

Running title: DNA methylation-based triage markers for cervical cancer

Corresponding author:

Monica Molano, PhD

For peer review only

Telephone +61 3 8345 3691

Fax +61 3 9347 8235

Email Monica.MolanoLuque@thewomens.org.au

Disclosure of Potential Conflicts of Interest:

AJV, JG, JB, MG, PJT, SGB, JMK have received subsidised test kits for research from Cepheid. MS, JMB, GT, DH have received donated test kits for research from Abbott, BD, Cepheid, Hologic, Qiagen, Roche, and Seegene. AJV and MS jointly lead the *Elimination of Cervical Cancer in the Western Pacific (ECCWP)* program with philanthropic funding support from the Minderoo Foundation and the Frazer Family Foundation; and equipment, tests and consumables donated by Cepheid for HPV-based cervical screening in PNG and Vanuatu. SMG is a member of the Global Advisory Board for HPV vaccines Merck, and has led investigator-initiated grants from Merck on HPV in young women. MM, DAM, SP, PB, GH, ZK, GLM, declare no conflicting interests.

Grant Support: This work was funded through the National Health and Medical Research Council, Australia, (NHMRC) grants 1013209 and 1104938 (A.J.V), Government of Papua New Guinea (ICRAS 297/1), the NHMRC Centre for Research Excellence in Cervical Cancer Control APP1135172 (JMB, MS, AV, SMG, JMK, DAM, DH) and NHMRC Investigator Grant APP1197951 (SMG).

For peer review only

For peer review only - http://bmjopen.bmj.com/site/about/guidelines.xhtml

Abstract

Introduction: WHO recommends HPV testing for cervical screening, with triage of high-risk human papillomavirus (hrHPV) positive women. However, there are limitations to effective triage for low resource, high burden settings, such as Papua New Guinea (PNG). We assessed the performance of host methylation as triage tools for predicting high-grade squamous intraepithelial lesions (HSIL) in self- and clinician-collected paired samples in an exploratory study of hrHPV+ women that participated in an HPV screen-and-same-day treat trial in PNG.

Methods: Methylation levels of *CADM1, MAL* and *miR124-2* were analysed by methylation-specific PCRs in 44 hrHPV+ women that participated in the cervical screening trial. The primary clinical endpoint in the trial was HSIL or SCC (HSIL+) using liquid-based-cytology (LBC)/p16-Ki67 stain. Diagnostic accuracy was assessed using area under curve receiver operating characteristics (AUC).

Results: 44 paired samples [4 squamous cell carcinomas (SCC), 19 HSIL, 4 low-grade squamous intraepithelial lesions (LSIL), 17 normal] were available for methylation analysis. In clinician-collected samples, *MAL* and *miR124-2* methylation levels were significantly higher in HSIL than normal/LSIL (p=0.013 and p<0.001 respectively). *miR124-2* was the best predictor of HSIL (AUC of 0.819), whilst *MAL* of SCC (0.856). In self-collected samples, *MAL* best predicted HSIL (0.595), whilst *miR124-2* SCC (0.812). Combined *miR124-2/MAL* methylation yielded sensitivity and specificity for HSIL+ of 90.5% (95%CI: 69.6-98.8) and 70% (45.7-88.1) respectively in clinician-collected samples, and 81.8% (59.7-94.8) and 47.6% (25.7-70.2) respectively in self-collected samples. *miR124-2/MAL* plus HPV16/HPV18 or extended genotyping improved sensitivity for HSIL+, but decreased specificity.

For peer review only - http://bmjopen.bmj.com/site/about/guidelines.xhtml

Conclusion: *miR124-2/MAL* methylation constitute a useful triage strategy to be investigated in a real-life 'HPV screen-triage-and-same-day treat' implementation program in LMIC.

Key words:

DNA Methylation, Cervical Cancer, Human papillomavirus, Diagnostic test , Epigenetics, Molecular diagnostics, Molecular Biology , Oncogenes.

For peer review only

Strengths and Limitations of this study

DNA host methylation is a promising triage strategy for early detection of cervical cancer in high income countries, but there is a knowledge gap regarding performance in LMIC, and with self-collected samples. This is the first study that:

- Explored the role of host methylation markers in the detection of HSIL in a group of self-collected samples of women that participated in an HPV screen-and-same-day treat trial in Papua New Guinea, a LMIC.
- Compared DNA methylation levels in self-collected samples and paired clinician-collected samples from a LMIC. This study showed that different cut off points must be established for the detection of HSIL according to the type of sample used.
- Explored the clinical performance of extended genotyping (HPV16/18/31/33/45/58) combined with host methylation analysis in the detection of HSIL as a triage strategy in a LMIC.
- These markers will be evaluated in a real-life 'HPV-screen- triage-and-same-day treat' program to strengthen the management of HPV+ women in LMIC.

Study limitations include

- Small sample size, which led to broad confidence intervals.
- LBC HSIL was the diagnostic reference standard rather than a histological end point as it was not feasible to obtain cervical biopsies due to limited specialist staff and infrastructure.

For peer review only - http://bmjopen.bmj.com/site/about/guidelines.xhtml

Introduction

Cervical cancer is the fourth most frequently diagnosed cancer and the fourth leading cause of cancer-related death in women, with an estimated 604,000 new cases and 342,000 deaths worldwide in 2020.[1] Age standardised rates are disproportionally higher in low- and middle-income countries (LMIC) than in high-income countries (18.8 vs 11.3 per 100,000 for incidence: 12.4 vs 5.2 per 100,000 for mortality), primarily due to disparities in access to effective prevention, screening and treatment strategies. [2, 3, 4]

To achieve cervical cancer elimination[5], many countries (mostly high income) are transitioning their cervical screening programs to HPV nucleic acid-based testing, which is more sensitive than cytology for detecting underlying high-grade disease. Furthermore, sample collection can be self-administered, which has been shown to increase screening participation in many settings. [6-11] Another important advantage is that molecular testing and subsequent treatment can be performed at point of care, greatly simplifying program delivery, particularly in low resource settings. These advances led the WHO, in 2021, to endorse HPV screen-and-treat guidelines for women in LMIC. [6]

An important limitation of HPV testing is that it suffers from a lower specificity than cytology, leading to concern that the treatment of all hrHPV+ women would result in significant overtreatment. [12] Hence, a second (triage) test is recommended to identify hrHPV+ women at highest risk of having underlying disease requiring treatment. The triage technologies most used are cytology, partial genotyping for HPV16 or 18, and in many low resource settings,

For peer review only - http://bmjopen.bmj.com/site/about/guidelines.xhtml

visual inspection using acetic acid or Lugol's iodine. [12] However, these triage approaches present challenges for sensitivity and/or specificity in LMIC settings. [8, 12]

Molecular triage tests are advantageous over current methods as they are less subjective and could be performed on multiple specimen types, including self-samples. In particular, detection of aberrant host gene DNA methylation patterns in promoter regions of some genes has shown promise as a triage marker in cervical cancer screening in high income countries. Aberrant methylation is pivotal in cervical carcinogenesis; it causes changes in gene expression, faulty condensation, and chromosomal instability. [13, 14] Increased DNA methylation has been associated with hrHPV persistence [13] and correlates with increasing severity of cervical disease. [14, 15] DNA methylation markers such as *CADM1, MAL* and *miR124-2* have shown good performance for the detection of CIN2+ among hrHPV+ women. [14, 16, 17] However, data on their performance has been based on studies conducted in high income settings and among highly screened populations. [18] There is currently no data on the performance of these markers among LMIC populations by using self-collected vaginal samples, nor in settings where HPV screening-and- same day-treatment strategies are used.

Papua New Guinea (PNG) has a high burden of cervical cancer, with age standardized incidence and mortality rates of 29.2 and 19.1 per 100,000, respectively, making it the second most common cancer among PNG women. [3] A trial study (HPV-STAT) performed in women from this country evaluated the clinical performance, treatment completion rates, adverse events profile, and acceptability of a fully integrated strategy, comprising point-of-care HPV DNA testing of self-collected specimens and same-day thermal ablation, for screening of cervical cancer. [19] This trial supported the introduction of HPV screening and same-day

For peer review only - http://bmjopen.bmj.com/site/about/guidelines.xhtml

treatment for the control of cervical cancer in LMIC. [19] In the current study, we explored the performance of *CADM1, MAL* and *miR124-2* DNA methylation markers alone and in combination with HPV16/18 and extended genotyping (HPV16/18/31/33/45/52/58) as a triage strategy for the detection of HSIL among HPV+ women that participated in this cervical screening trial. A direct comparison was also performed for each methylation marker, between paired self- and clinician-collected samples.

For peer review only

For peer review only - http://bmjopen.bmj.com/site/about/guidelines.xhtml

Materials and Methods

Study population and design of the trial

The exploratory study was conducted as a sub-study of HPV-STAT ('HPV self-collect, test and-same-day-treat') a prospective, single-arm intervention trial among women attending cervical screening services at two clinical sites in PNG. The trial is registered with ISRCTN, ISRCTN13476702 (https://www.isrctn.com/editorial/retrieveFile/3ed9173e-cce5-4158-a586-577775f0cbdd/35731). Study design, recruitment and protocols have been described. [19] Briefly, between June 5, 2018, and Jan 6, 2020, 4285 women aged 30 to 59 years attending clinics for routine cervical screening at Modilon Hospital (Madang Province) and Mt Hagen General Hospital (Western Highlands Province), who meet the inclusion criteria were invited to participate. Women who were pregnant or who had given birth in the past 6 weeks, or women who had a history of cervical cancer or hysterectomy, were excluded. [19] Those women able to provide written informed consent were eligible to participate, enrolled sequentially and collected a vaginal specimen using a cytobrush ("Just for Me", Preventative Oncology International, Cleveland Heights, Ohio), which was placed into a 20 ml ThinPrep vial (Hologic, Marlborough, MA). [19]

Point Of Care HPV testing and follow-up assessment

For self-collected vaginal specimens, a 1 ml aliquot was tested for the presence of oncogenic HPV genotypes using the Xpert HPV Test (GeneXpert; Cepheid, Sunnyvale, CA, USA) as per the manufacturer's instructions. Results were reported as 'HPV16', 'HPV18/45', and 'Other HPV' (a summary result for HPV31, 33, 35, 39, 51, 52, 56, 58, 59, 66 and 68) and were provided to women before midday to allow sufficient time for same-day counselling, pelvic examination

For peer review only - http://bmjopen.bmj.com/site/about/guidelines.xhtml

and treatment/referral as indicated. [19] All women with a negative HPV test were informed of their results and advised to return to the clinic for HPV-based screening in five years.

HPV-positive women provided a cervical specimen collected by a clinician using a Cervex-Brush Combi (Rovers Medical Devices, Oss, The Netherlands), placed in a 20 ml ThinPrep vial, and stored at 4°C prior to shipment to Victorian Cytology Service Foundation (VCS) in Melbourne, Australia for liquid-based cytology (LBC) and p16/Ki67 dual stain cytology. A 15% random sample of HPV negative women were also asked to provide a clinician-collected cervical specimen for LBC, as above.

Cytological assessment

LBC was carried out in accordance with established laboratory procedures at VCS. Slides were independently assessed by two cytologists blinded to HPV test results. Where both cytologists agreed on a diagnosis of HSIL or worse (HSIL+), a final diagnosis was recorded. If the assessment differed, dual p16/Ki-67 immunostaining was carried out by using CINTec PLUS Cytology (Roche Diagnostics, Pleasanton, USA) to make a final diagnosis. [19] The primary clinical endpoint was then HSIL or SCC (HSIL+) using LBC/p16-Ki67 stain. LBC was accepted as a reference standard rather than histology, which is the gold reference in high-resource settings. It is not currently feasible from a staffing and logistical perspective to provide colposcopy or to collect cervical biopsies for histological examination in PNG.

Participants in the exploratory study and design

The sub-study included 44 paired cervical and vaginal samples from women participating in the trial at Mt Hagen General Hospital (Mount Hagen, Western Highlands Province). This included all 23 hrHPV+ HSIL or SCC (19 HSIL and 4 SCC) cases identified on LBC conducted at

For peer review only - http://bmjopen.bmj.com/site/about/guidelines.xhtml

For peer review only

VCS by the end of 2018 and 21 randomly selected hrHPV+ normal/LSIL samples (17 normal LBC and 4 LSIL). Sample size selection was driven by a published study by Li D et al., 2015 that showed through simulation studies and real data from 2 studies downloaded from the NCBI Gene Expression Omnibus that at least 12 specimens in each group is needed to detect truly differential DNA methylation with enough power (≥ 80%), reproducible data and consistence when using different statistical methods. [20]

Molecular biologists and technicians were blinded to point of care HPV and clinical diagnosis.

DNA Extraction and HPV specific typing

DNA was extracted as described previously, quantitated by Qubit® Fluorometer (Life technologies, California, USA), and assessed for integrity by quantitative PCR amplification of a 260 base-pair product of the human beta-globin gene. [21] In addition, DNA extracted from cervical cell line SiHa (1-2 copies of HPV16 per cell, ATCC Cat# HTB-35, RRID:CVCL_0032; American Type Culture Collection (ATCC), Manassas, Virginia, USA; 100 ng) was used as positive control for methylation analysis. This DNA was confirmed to have only HPV16 by HPV typing for 28 HPV types (Anyplex II HPV28 Detection, Seegene, Seoul, South Korea.

HPV genotyping was performed using the Anyplex II HR HPV Detection multiplex assay (Seegene, Seoul, South Korea), which detects 14 oncogenic HPV genotypes (16, 18, 31, 33, 35, 39, 45, 51, 52, 56, 58, 59, 66, 68) and an internal control, according to manufacturer's recommendations.

For peer review only - http://bmjopen.bmj.com/site/about/guidelines.xhtml

Bisulphite DNA modification and Quantitative methylation-specific PCR for CADM1, MAL and miR124 genes

DNA from the paired samples (1-100ng) and control (SiHa, 100 ng) were bisulphite treated using Methylamp DNA modification Kit (Epigentek, Brooklyn, NY, USA) as per the manufacturer's instructions. Modified DNA was eluted in 40µL of the Methylamp elution buffer. Samples without the adequate concentration were excluded from the bisulphite modification and methylation analysis.

Quantitative methylation-specific PCR (qMSP) targeting CpG sites in promoter regions of *CADM1* (promoter region M9), *MAL* (promoter region M1) and *miR124-2* (promoter region 2) were performed as previously described, [14] with minor modifications. Due to the use of a different platform (Light Cycler 480 II, Roche), validations of the qPCRs were performed utilising duplicated dilution series of SiHa cell line (intra and inter assays of reproducibility) and by using a training panel of samples as reported previously. [21]

Statistical Analysis

The percentage of methylation was calculated as described previously. [22] Differences in the median percentage methylation by disease grade (SCC, HSIL, LSIL/normal) were visualised using box and whisker plots and compared using Wilcoxon test (between groups) and Kruskal-Wallis (overall analysis). [20, 21] Area under the receiver operating characteristic curve (AUC) was used to assess the ability of the methylated genes to classify HSIL or SCC, providing two main outcomes: the diagnostic accuracy of the test and the optimal cut off point value for dichotomization of the methylation test. [23] The optimal cut off-point was calculated by using the maximum sum of sensitivity and specificity. [24] Sensitivity and specificity were determined

For peer review only - http://bmjopen.bmj.com/site/about/guidelines.xhtml

For peer review only

for the following triage strategies: (I) single gene methylation analysis, (II) combination of methylation markers, (III) HPV 16 genotyping, (IV) combination of HPV16/18 genotyping, (V) combination of HPV 16/18/31/33/45/52/58 genotyping, (VI) HPV16/18 with single and combined methylation analysis, and (VII) HPV 16/18/31/33/45/52/58 with single and combined methylation analysis.

Median percentage methylation for each marker was compared for self- and clinician-collected vaginal paired specimens using the Wilcoxon signed rank test. The vaginal specimen was considered the test while the cervical specimen acted as the non-reference standard. Agreements between DNA methylation results from cervical and vaginal specimens were determined using the optimal cut off point established for each gene by calculating positive percent agreement (PPA), negative percent agreement (NPA) and overall percent agreement (OPA) as per recommendations by the Food and Drug Administration, USA. [25]

The results were analysed by using XLSTAT and the statistical platform R studio (v4.0.1) and programs ggplots2 (v3.3.2), ggpubr (v0.4), pROC (v1.16.2) and cutpointr (v1.1.1). [21, 24]

We used the STARD reporting guidelines for reporting diagnostic accuracy studies. [26]

Ethical considerations

Approval of the trial was provided by the Medical Research Advisory Committee (MRAC) of the Papua New Guinea National Department of Health (approval number 17.36), the Institutional Review Board of the Papua New Guinea Institute of Medical Research (IRB 1712), and the Human Research Ethics Committee (HREC) of UNSW Australia (approval number HC17631). Written informed consent was obtained from all participants prior to enrolment.

For peer review only - http://bmjopen.bmj.com/site/about/guidelines.xhtml

Results

DNA Methylation of CADM1, MAL and miR124-2 and associations with lesion grade

Overall, 44 hrHPV+ paired cervical and vaginal samples from women participating in the trial at Mt Hagen General Hospital were used [23 cases (19 HSIL and 4 SCC 8) and 21 normal/LSIL (17 normal LBC and 4 LSIL)]. Of these samples, 41 (93.2%) clinician-collected (4 SCC, 17 HSIL, 4 LSIL and 16 normal LBC) and 43 (97.7%) self-collected samples (4 SCC, 18 HSIL, 4 LSIL and 17 normal LBC) were deemed assessable. The methylation analysis did not interfere with the clinical management of the women, and thus did not induce any adverse effects.

In clinician-collected samples DNA methylation of *MAL* was significantly higher in HSIL (median 2.0%; 95%CI:0.7-20.4) and SCC (63.3%; 46.8-67.9) than normal/LSIL samples (1.4%; 0.8-3.1) (p=0.013 and p=0.004, respectively) (Figure 1, top central panel). Methylation of *mirR124-2* was also significantly higher in HSIL (5.6%; 3.9-20.5) and SCC (24.1%; 12.4-34.5) than normal/LSIL samples (2.6%; 1.1-5.1) ($p<0.001$ and $p=0.033$, respectively) (Figure 1, top right panel). Methylation levels of both genes did not differ significantly between HSIL and SCC. Methylation of *CADM1* did not differ significantly with increasing cytology grade (Figure 1, top left panel).

Using clinician-collected samples, ROC analysis showed that *miR124-2* was the best methylation marker to distinguish SCC and HSIL (from normal/LSIL), with an AUC of 0.850 and 0.819, respectively (Figure 2). Methylation of *MAL* showed higher AUC for detecting SCC (0.856) than HSIL (0.700). *CADM1* methylation showed poor performance for detecting SCC or HSIL with AUC of 0.700 and 0.607 respectively (Figure 2).

For peer review only - http://bmjopen.bmj.com/site/about/guidelines.xhtml

In self-collected samples, DNA methylation of *MAL* was significantly higher in SCC (median 6.9%; 95%CI:0.1-26.1) than normal/LSIL samples (1.1%; 0.5-3.2) (p=0.032) (Figure 1, lower centre panel). Methylation levels did not differ significantly between HSIL and normal/LSIL or HSIL and SCC. DNA methylation of *miR124-2* was significantly higher in SCC (3.3%; 2.6-8.5) than HSIL (1.5%; 1.1-2.6) and normal/LSIL (1.3%; 0.9-2.5) (p=0.049 and p=0.042, respectively) (Figure 1, lower right panel). Methylation levels did not differ significantly between HSIL and normal/LSIL. As found for clinician-collected samples, methylation of *CADM1* did not differ significantly with increasing cytology grade (Figure 1, lower left panel).

ROC analysis showed that from self-collected samples, *miR124-2* was the best methylation marker to distinguish SCC, but not HSIL, from normal/LSIL with an AUC of 0.812 and 0.563, respectively (Figure 2). Methylation of *MAL* or *CADM1* showed a lower clinical performance for SCC (AUC of 0.725 and 0.750, respectively) than *miR124-2*, and a similar performance for HSIL (AUC of 0.595 and 0.515, respectively) (Figure 2).

Diagnostic performance of DNA methylation markers for the detection of HSIL or SCC

Diagnostic performance of individual DNA methylation markers for detecting HSIL+ in clinician-collected samples showed that *miR124-2* methylation had the highest sensitivity of 81.0% (95%CI: 58.1-94.6) and *MAL* methylation the highest specificity of 90.0% (68.3-98.8) (Table 1). A combination of *miR124-2* and *MAL* showed the optimal diagnostic performance with a sensitivity of 90.5% (69.6-98.8) and specificity of 70.0% (45.7-88.1). A combination of *miR124-2/MAL* and *CADM1* showed a maximal sensitivity of 100% (95% CI 83.9-100) at the expense of a low specificity, 25% (8.7-49.1).

For peer review only - http://bmjopen.bmj.com/site/about/guidelines.xhtml

In self-collected samples, *miR124-2* methylation had a sensitivity of 77.3 % (95% CI 54.6-92.2) for detecting HSIL+, but much lower specificity 47.6% (25.7-70.2) and *MAL* showed the best specificity 90.5% (69.6-98.8) at the expense of a low sensitivity 32.0% (13.9-54.9) (Table 1). A combination of *miR124-2* and *MAL* improved the diagnostic performance with a sensitivity of 81.8% (95% CI 59.7-94.8) and specificity of 47.6% (25.7-70.2). A combination of *miR124-2/MAL* and *CADM1* increased the sensitivity 90.9% (70.8-98.9) at the expense of a low specificity 23.8% (8.2-47.2) (Table 1).

For peer review only

For peer review only - http://bmjopen.bmj.com/site/about/guidelines.xhtml

327 ***Table 1.*** Performance of host DNA methylation markers for detection of HSIL+

Sample type	Gene[a]	Percent methylation cut-off	Sensitivity % (95% CI)	Specificity % (95% CI)
Cervical, clinician-collected	*miR124-2*	3.7	81.0 (58.1-94.6)	70.0 (45.7-88.1)
	MAL	0.25	57.1 (34.0-78.2)	90.0 (68.3-98.8)
	CADM1	3.5	80.9 (58.1-94.6)	40.0 (19.1-63.9)
	miR124-2/MAL	3.7/0.25	90.5 (69.6-98.8)	70.0 (45.7-88.1)
	miR124-2/CADM1	3.7/3.5	95.2 (76.2-99.9)	25.0 (8.7-49.1)
	MAL/CADM1	0.25/3.5	95.2 (76.2-99.9)	35.0 (15.4-59.2)
	miR124-2/MAL/CADM1	3.7/0.25/3.5	100 (83.9-100)	25.0 (8.7-49.1)
Vaginal, self-collected	*miR124-2*	1.2	77.3 (54.6-92.2)	47.6 (25.7-70.2)
	MAL	0.1	32.0 (13.9-54.9)	90.5 (69.6-98.8)
	CADM1	3.9	68.2 (45.1-86.1)	33.3 (14.6-57.0)
	miR124-2/MAL	1.2/0.1	81.8 (59.7-94.8)	47.6 (25.7-70.2)
	miR124-2/CADM1	1.2/3.9	86.4 (65.1-97.1)	23.8 (8.2-47.2)
	MAL/CADM1	0.1/3.9	81.8 (59.7-94.8)	28.6 (11.3-52.2)
	miR124-2/MAL/CADM1	1.2/0.1/3.9	90.9 (70.8-98.9)	23.8 (8.2-47.2)

328 [a] Positive for at least one of the indicated host genes

329

330 Shading rows show markers or combination of markers with the best diagnostic performance and those with the maximal sensitivity for detection of HSIL+

For peer review only - http://bmjopen.bmj.com/site/about/guidelines.xhtml

Diagnostic performance of HPV specific typing combined with DNA methylation markers for the detection of HSIL or SCC

Diagnostic performance of HPV16 for detecting HSIL+ in clinician-collected samples showed -a sensitivity of 52.2% (95% CI 30.6-73.2) and specificity of 81.0% (95% CI 58.1-94.6) (Table 2). HPV16 and HPV18 combined showed a sensitivity of 60.9% (95% CI 38.5-80.3) and specificity of 81.0% (95% CI 58.1-94.6). A combination of *miR124-2/MAL* methylation markers and HPV16/18 genotyping showed a satisfactory diagnostic performance with a sensitivity 95.2% (95% CI 76.2-99.9) and specificity of 55% (95% CI 31.5-76.9). A combination of *MAL/CADM1* and HPV16/18 genotyping showed a maximal sensitivity of 100% (95% CI 85.9-100) at the expense of a specificity of 30% (11.9-54.3). Using HPV16/18/31/33/45/ 52/58 genotyping, either as a single marker or combined with different methylation markers, showed high sensitivities but at the expense of low specificities (Table 2).

For self-collected samples, detection of individual and combined HPV genotypes had the same diagnostic performance as seen in clinician-collected samples (Table 2). A combination of *miR124-2 or miR124/MAL and* HPV16/HPV18 showed similar sensitivity found for clinician-collected samples of 95.5% (95%CI:77.2-99.9), but reduced specificity (33.3%, 95%CI: 14.6-50.7). A combination of *miR124-2/CADM1* and HPV16/18 genotyping showed a maximal sensitivity of 100% (95% CI 84.6-100) and specificity of 23.8% (8.2-47.2). Adding HPV16/18/31/33/45/58 genotyping combined with the different methylation markers showed high sensitivities at expenses of a very low specificities (Table 2).

For peer review only - http://bmjopen.bmj.com/site/about/guidelines.xhtml

Table 2. Performance of HPV genotyping combined with methylation markers for the detection of HSIL+

Sample type	Combined HPV/methylation detection for decision making			Sensitivity % (95% CI)	Specificity (95%CI)
	**HPV genotype [a]**	**Host Gene[b]**	**Percent methylation cut-off**		
Cervical, clinician-collected	16	NA	NA	52.2 (30.6-73.2)	81.0 (58.1-94.6)
	16,18	NA	NA	60.9 (38.5-80.3)	81.0 (58.1-94.6)
	16,18,31,33,45,52,58	NA	NA	95.7 (78.1-99.9)	33.3 (14.6-56.9)
	16,18	*miR124-2*	3.7	85.7 (63.7-97.0)	55.0 (31.5-76.9)
	16,18,31,33,45,52,58	*miR124-2*	3.7	95.2 (76-2-99.9)	25.0 (8.7-49.1)
	16,18	*MAL*	0.25	76.2 (52.8-91.8)	70.0 (45.7-88.1)
	16,18,31,33,45,52,58	*MAL*	0.25	100 (84.6-100)	14.3 (3.1-36.3)
	16,18	*CADM1*	3.5	90.5 (69.6-98.8)	35.0 (15.4-59.2)
	16,18,31,33,45,52,58	*CADM1*	3.5	95.2 (76.2-99.9)	20.0 (5.7-43.7)
	16,18	*miR124-2/MAL*	3.7/0.25	95.2 (76.2-99.9)	55.0 (31.5-76.9)
	16,18,31,33,45,52,58	*miR124-2/MAL*	3.7/0.25	100 (83.9-100)	25.0 (8.7-49.0)
	16,18	*miR124-2/CADM1*	3.7/3.5	95.2 (76.2-99.9)	25.0 (8.7-49.1)
	16,18,31,33,45,52,58	*miR124-2/CADM1*	3.7/3.5	95.2 (76.2-99.9)	15.0 (3.2-37.9)
	16,18	*MAL/CADM1*	0.25/3.7	100 (85.9-100)	30.0 (11.9-54.3)
	16,18,31,33,45,52,58	*MAL/CADM1*	0.25/3.7	100 (83.9-100)	20.0 (5.7-43.7)
Vaginal, self-collected	16	NA	NA	52.2 (30.6-73.2)	81.0 (58.1-94.6)
	16,18	NA	NA	60.9 (38.5-80.3)	81.0 (58.1-94.6)
	16,18,31,33,45,52,58	NA	NA	95.7 (78.1-99.9)	33.3 (14.6-56.9)
	16,18	*miR124-2*	1.2	95.5 (77.2-99.9)	33.3 (14.6-50.7)
	16,18,31,33,45,52,58	*miR124-2*	1.2	100 (84.6-100)	14.3 (3.1-36.3)
	16,18	*MAL*	0.1	68.2 (45.1-86.1)	71.4 (47.8-88.7)
	16,18,31,33,45,52,58	*MAL*	0.1	95.5 (77.2-99.9)	28.6 (11.3-52.2)
	16,18	*CADM1*	3.9	90.9 (70.8-98.9)	33.3 (14.6-56.9)

For peer review only

For peer review only - http://bmjopen.bmj.com/site/about/guidelines.xhtml

353

16,18,31,33,45,52,58	*CADM1*	3.9	100 (84.6-100)	9.5 (1.2-30.4)
16,18	*miR124-2/MAL*	1.2/0.1	95.5 (77.2-99.9)	33.3 (14.6-57.0)
16,18,31,33,45,52,58	*miR124-2/MAL*	1.2/0.1	100 (84.6-100)	14.3 (3.1-36.3)
16,18	*miR124-2/CADM1*	1.2/3.9	100 (84.6-100)	23.8 (8.2-47.2)
16,18,31,33,45,52,58	*miR124-2/CADM1*	1.2/3.9	100 (84.6-100)	4.8 (0.1-23.8)
16,18	*MAL/CADM1*	0.1/3.9	90.9 (70.8-98.9)	28.6 (11.3-52.2)
16,18,31,33,45,52,58	*MAL/CADM1*	0.1/3.9	100 (84.6-100)	9.5 (1.2-30.4)

354 [a] Detection of at least one of the indicated HPV genotypes
355
356 [b] Positive for at least one of the indicated host genes
357
358 Shading rows show markers or combination of markers with the best diagnostic performance and those with the maximal sensitivity for detection of HSIL+

For peer review only

Comparison of levels of methylation between paired self-collected and clinician-collected samples according to cytological results

For each cytological diagnosis, there was generally a lower percentage of methylation for each marker in vaginal samples compared with their paired cervical samples (Figure 3). However, only for *miR124-2* did this difference reach significance in HSIL when using the Wilcoxon signed rank test (Figure 3).

Agreement analysis of paired self-collected and clinician-collected samples for *miR124-2*, *MAL* and *CADM1* methylation

Analysis was performed for 40 patients with paired, assessable samples. A first analysis was performed without considering the underlying disease status. The analysis found OPA, PPA and NPA were 62.5%, 77.3% and 44.4% respectively for *miR124-2* methylation, 77.5%, 46.2% and 92.6% respectively for *MAL* methylation, and 57.5%, 67.9% and 33.3% respectively for *CADM1*.

In analyses limited to HSIL+ paired clinician- and self-collected samples, the OPA, PPA and NPA were 75.0% (53.1-88.8), 81.3% (57.0-93.4) and 50.0% (15.0-85.0) respectively for *miR124-2* methylation, 65.0% (43.3-81.9), 45.5% (21.3-72.0) and 88.9% (56.5-98.0) respectively for *MAL* methylation; and 65.0% (43.3-81.9), 68.8% (44.4-85.8) and 50.0% (15.0-85.0) respectively for *CADM1*.

For peer review only - http://bmjopen.bmj.com/site/about/guidelines.xhtml

Discussion

In this study, we explored *CADM1*, *MAL* and *miR124-2* DNA methylation using clinician- and self-collected HPV+ samples from PNG, a setting with a high burden of cervical disease. In clinician-collected cervical samples, DNA methylation of *MAL* and *miR124-2* were significantly higher in HSIL/cancer compared with normal/LSIL samples, while the methylation of *CADM1* failed to distinguish between cytology grades. Methylation of *miR124-2* showed the highest AUC to distinguish HSIL from normal/LSIL and the second-highest performance to predict SCC, with AUC values above 0.819. Exploration of diagnostic performance of *miR124-2* in combination with *MAL* for detection of HSIL+, showed an excellent performance (sensitivity 90.5%, specificity 70%). Similar findings were obtained with self-collected samples, though their performance was reduced. Overall, *miR124-2* in combination with *MAL* DNA methylation perform well for the detection of HSIL+ in women from LMIC. Addition of HPV16/18 or extensive genotyping will improve sensitivity at the expense of lower specificity and higher referrals to immediate treatment, but still lower that using the current algorithm. *miR124-2/MAL* methylation markers are suitable for evaluation in LMIC-based large field studies for triage of hrHPV+ women to identify high-grade disease.

Increased levels of *miR124-2*, *CADM1* and *MAL* methylation according to lesion grade have been observed in studies performed in clinician-collected cervical samples from high-income populations. [27-29] In addition, the diagnostic performance of *miR124-2* methylation alone or in combination with *CADM1* and *MAL* has shown consistently high sensitivity and specificity for the detection of CIN3+ in HPV+ clinician-collected cervical specimens in women from high-income countries (sensitivity 68.0–94.7%, specificity 50.7–78.9%). [30]

For peer review only - http://bmjopen.bmj.com/site/about/guidelines.xhtml

Few studies have been performed in LMIC using clinician-collected samples. An analysis of the same markers in HPV+ and HIV+ women from Kenya found the AUC of *miR124-2* methylation for CIN2+ and CIN3+ were 0.730 and 0.810 respectively, [31] marginally lower to those obtained in the current exploratory study. However, these results are not directly comparable to ours as their lesion grade was based on histological diagnosis and HIV+ participants. In the current study, we were unable to stratify by HIV status, but assume a low HIV prevalence as the HIV prevalence in PNG general population is <1%. Two other studies performed in South Africa and Thailand (upper middle-income countries) also showed good performance of some of these markers, alone or in combination, for the detection of HSIL+. [32, 33]

CADM1 was not a good diagnostic marker, consistent with the findings from two other studies performed in high-income countries. [34, 35]

No research has been conducted looking methylation at self-collected samples in LMIC. Diagnostic performance of *miR124-2* for detection of HSIL+ in self-collected samples showed a sensitivity of 77.3% and specificity of 47.6%, while combining *miR124-2* with *MAL* increased the sensitivity to 81.8%, showing promise for self-sampling applications. While the sensitivity/specificity was slightly lower than clinician-collected samples, this may be offset by higher screening rates achieved by this more acceptable method of collection and reduced referral to colposcopy/treatment compared to current screening algorithms.

By comparison, studies of *miR124-2* and *MAL* single or in combination with other methylation markers for detection of CIN3+ in self-collected samples from high-income settings, have shown a wide range of sensitivities and specificities with values between 37.5–78.4% and 47.2–98% respectively. [36-39] This wide variation may be explained by variable methodology [self-sampling collection devices, methylation detection assays, genes analysed, thresholds to

For peer review only - http://bmjopen.bmj.com/site/about/guidelines.xhtml

define methylation positivity, study populations, disease end points (cytological HSIL or histological CIN2/3+), etc].

A triage strategy combining HPV16/18 genotyping and methylation analysis in clinician-collected samples has shown promise in recent studies conducted in high-income settings. [37,38,40,41] In our study, combining HPV16/18 detection with *miR124-2/MAL* methylation improved sensitivity (increasing from 90.5 to 95.2%), but reduced specificity (decreasing from 70.0% to 55.0%). In self-collected samples the sensitivity was similar to clinician-collected samples (95.5%) but the specificity was lower (33.3%). By comparison, in self-collected samples from a high-income country, methylation analysis of *miR124-2/MAL* combined with HPV16/18 genotyping had a sensitivity for CIN3+ of 77.6% and specificity of 54.8%. [40] A recent prospective cohort study in an upper middle-income country analysing methylation markers ZNF671/ASTN1/ITGA4/RXFP3/SOX17/DLX1 showed a sensitivity and specificity of 96.6% and 58.3% respectively to detect CIN2+ when combining methylation and HPV16/18. [42]

Combining extended genotyping (HPV16/18/31/33/45/58) with miR124-2/MAL methylation had similar sensitivity to *miR124/MAL/HPV16/18* testing, but in our setting showed lower specificity. Regardless of study population, HPV prevalence, sample type, marker panel and age, the combination of host cell DNA methylation analysis with partial or extended genotyping could serve as a potential triage strategy for hrHPV+ women, at the expense of a higher referral to treatment but still lower that using the current algorithm.

Self-collected samples generally had lower levels of methylation than paired clinician-collected samples. This is most likely due to lower diseased/normal cell ratios in self-collected vaginal vs clinician-collected cervical samples collected directly from the cervix. For example, values for the GynTect® assay (detecting six methylation markers) were significantly lower for

For peer review only - http://bmjopen.bmj.com/site/about/guidelines.xhtml

self-collected than clinician-collected samples and the overall concordance was moderate (kappa 0.394). [43] Further work to identify markers suitable for self-collection should consider using self-collected samples for this purpose. [44]

Limitations of this study include the small sample size which led to broad confidence intervals; however, it aimed to provide estimated effects (point estimates with confidence intervals) for all measures of interest which are of great value for the planning of subsequent studies in LMIC. It was not feasible to conduct cervical biopsies in this setting due to limited specialist staff and infrastructure; hence LBC HSIL was the diagnostic reference standard rather than a histological end point. This is unlikely to have led to disease misclassification as all slides were assessed by two independent experienced cytologists, and dual p16/Ki-67 immunostaining was performed to resolve disagreements. A triage test based on methylation markers could be highly suited to regions without access to colposcopy, where the combination of HPV detection, methylation analysis and cytology could confirm HSIL+ in the absence of colposcopy examination. Additional information is required to understand the long-term safety of methylation markers in screening programs, including longitudinal data from a low-resource setting and analysis of methylation in self-collected samples from general population and HIV+ women.

In conclusion, DNA methylation of *miR124-2/MAL* alone or in combination with HPV16/18 is very promising for the prediction of HSIL/SCC as a triage test among women with oncogenic HPV infections in clinician-collected samples. Self-collected samples showed reduced specificity compared to clinician-collected samples, indicating a need to continue working in the identification of additional biomarkers specific for this sample type for a greater

For peer review only - http://bmjopen.bmj.com/site/about/guidelines.xhtml

diagnostic performance. Future work will focus on the evaluation of these results in large-scale follow-up studies in different populations and performing real-life pilot HPV-based 'screen, triage and treat' implementation programs to strengthen the management of HPV+ women in LMIC.

Acknowledgements

We thank the women who participated in this research and their families and community, without whom this work would not have been possible. Special thanks to the PNG study team who collaborated to the fieldwork, and Cepheid (Sunnyvale, CA, USA) for donating Xpert HPV cartridges.

Authors' Contributions

MM: Conceptualization, methodology, laboratory work, writing original draft, software analysis. DAM. Supervision, methodology, investigation, writing–review and editing. GT: Coordinated the evaluation of liquid-based cytology specimens, review and editing. SMG: Conceived the original trial, supervision, funding acquisition, project administration, writing—review and editing. SP: Software analysis, data curation, review and editing. PB, DH, PJT: Investigation, laboratory work, review and editing. SGB: Conceived the original trial, coordinated clinical implementation, review and editing. JB, JG, ZK, GMM: coordinated clinical implementation in the original trial, review and editing. AC, RH investigation, review and editing. JMB, MS, JMK: Conceived the original trial, funding acquisition, review and editing.

For peer review only - http://bmjopen.bmj.com/site/about/guidelines.xhtml

For peer review only

AJV: Conceived the original trial, conceptualization, funding acquisition, project administration, writing, review and editing GM: Conceptualization, supervision, funding acquisition, investigation, project administration, writing, review and editing.

For peer review only

For peer review only - http://bmjopen.bmj.com/site/about/guidelines.xhtml

References

1. Sung H, Ferlay J, Siegel RL, et al. Global cancer statistics 2020: GLOBOCAN estimates of incidence and mortality worldwide for 36 cancers in 185 countries. CA Cancer J Clin. 2021;71(3):209-249.

2. Lemp JM, De Neve JW, Bussmann H, et al. Lifetime prevalence of cervical cancer screening in 55 low- and middle-income countries. JAMA. 2020;324:1532-1542.

3. Ferlay J, Ervik M, Lam F, et al. Global Cancer Observatory: Cancer Today. Lyon, France: International Agency for Research on Cancer. Available from: https://gco.iarc.fr/today, accessed 20 07 2021.

4. PATH. Global HPV Vaccine Introduction Overview: projected and current national introductions, demonstration/pilot projects, gender-neutral vaccination programs, and global HPV vaccine introduction maps (2006-2023). PATH; 2020. Accessed November 23, 2020. Path.org/resources/global-hpv-vaccine-introduction-overview/ 179.

5. Simelela PN. WHO global strategy to eliminate cervical cancer as a public health problem: An opportunity to make it a disease of the past. Int J Gynaecol Obstet. 2021;152(1):1-3.

6. WHO. WHO guideline for screening and treatment of cervical pre-cancer lesions for cervical cancer prevention, second edition. Geneva: World Health Organization; 2021

7. Toliman P, Badman SG, Gabuzzi J, et al. Field Evaluation of Xpert HPV Point-of-Care Test for Detection of Human Papillomavirus Infection by Use of Self-Collected Vaginal and Clinician-Collected Cervical Specimens. J Clin Microbiol. 2016;54(7):1734-7.

8. Toliman PJ, Kaldor JM, Badman SG, et al. Evaluation of self-collected vaginal specimens for the detection of high-risk human papillomavirus infection and the prediction of high-grade

For peer review only

For peer review only - http://bmjopen.bmj.com/site/about/guidelines.xhtml

cervical intraepithelial lesions in a high-burden, low-resource setting. Clin Microbiol Infect. 2019;25:496-503.

9. Arbyn M, Smith SB, Temin S, et al. Detecting cervical precancer and reaching under screened women by using HPV testing on self samples: updated meta-analyses. BMJ (Clinical research ed) 2018; 363: k4823.

10. De Pauw H, Donders G, Weyers S, et al. Cervical cancer screening using HPV tests on self-samples: attitudes and preferences of women participating in the VALHUDES study. Arch Public Health. 2021;79(1):155.

11. Hawkes D, Keung MHT, Huang Y, et al. Self-Collection for Cervical Screening Programs: From Research to Reality. Cancers (Basel). 2020;12(4):1053.

12. Murillo R, Gamboa O, Hernández G, et al. Accuracy of combined molecular and morphology-based triage for HPV-positive women in routine cervical cancer screening services from Colombia. Prev Med. 2021;153:106801.

13. Mirabello L, Sun C, Ghosh A, et al. Methylation of human papillomavirus type 16 genome and risk of cervical precancer in a Costa Rican population. J. Natl Cancer Inst. 2012;104:556–565.

14. De Strooper LM, van Zummeren M, Steenbergen RD, . CADM1, MAL and miR124-2 methylation analysis in cervical scrapes to detect cervical and endometrial cancer. J Clin Pathol. 2014; 67(12): 1067-71.

15. Cook D A, Krajden M, Brentnall A R, et al. Evaluation of a validated methylation triage signature for human papillomavirus positive women in the HPV FOCAL cervical cancer screening trial. Int J Cancer. 2018; 144: 2587–2595.

For peer review only - http://bmjopen.bmj.com/site/about/guidelines.xhtml

16. Bonde J, Floore A, Ejegod D, et al. Methylation markers FAM19A4 and miR124-2 as triage strategy for primary human papillomavirus screen positive women: A large European multicenter study. Int J Cancer. 2021;148(2):396-405.

17. Verhoef VMJ, van Kemenade FJ, Rozendaal L, et al. Follow-up of high-risk HPV positive women by combined cytology and bi-marker CADM1/MAL methylation analysis on cervical scrapes. Gynecol Oncol. 2015;137(1):55–59.

18. Zhang L, Tan W, Yang H, et al. Detection of Host Cell Gene/HPV DNA Methylation Markers: A Promising Triage Approach for Cervical Cancer. Front Oncol. 2022;12:831949.

19. Vallely AJ, Saville M, Badman SG, et al. Point-of-care HPV-DNA testing of self-collected specimens and same-day thermal ablation for the early detection and treatment of cervical pre-cancer in high-burden, low-resource settings: Results of a prospective intervention trial in Papua New Guinea (HPV-STAT). Lancet Glob Health. 2022 Sep;10(9):e1336-e1346.

20. Li D, Xie Z, Pape ML, Dye T. An evaluation of statistical methods for DNA methylation microarray data analysis. BMC Bioinformatics. 2015 Jul 10;16:217.

21. Phillips S, Cassells K, Garland SM, et al. Gene methylation of CADM1 and MAL identified as a biomarker of high grade anal intraepithelial neoplasia. Sci Rep. 2022;12(1):3565.

22. Hesselink AT, Heideman DA, Steenbergen RD, et al. Methylation marker analysis of self-sampled cervico-vaginal lavage specimens to triage high-risk HPV-positive women for colposcopy. Int J Cancer. 2014;135(4):880-6.

23. Unal I. Defining an Optimal Cut-Point Value in ROC Analysis: An Alternative Approach. Comput Math Methods Med. 2017;2017:3762651.

24.Thiele C & Hirschfeld G. Cutpointr: Improved estimation and validation of optimal cutpoints in R. J. Stat. Softw. 2021;98(11): 1–27.

For peer review only - http://bmjopen.bmj.com/site/about/guidelines.xhtml

25. Food, U. S. “Drug Administration. Statistical Guidance on Reporting Results from Studies Evaluating Diagnostic Tests-Guidance for Industry and FDA Staff.” (2007).

26. Bossuyt PM, Reitsma JB, Bruns DE, et al. STARD 2015: An Updated List of Essential Items for Reporting Diagnostic Accuracy Studies.

27. Hesselink AT, Heideman DA, Steenbergen RD, et al. Combined promoter methylation analysis of CADM1 and MAL: an objective triage tool for high-risk human papillomavirus DNA-positive women. Clin Cancer Res. 2011;17:2459–2465.

28. Bierkens M, Hesselink AT, Meijer CJ, et al. CADM1 and MAL promoter methylation levels in hrHPV-positive cervical scrapes increase proportional to degree and duration of underlying cervical disease. Int J Cancer. 2013;133:1293–1299

29. Clarke MA, Luhn P, Gage JC, et al. Discovery and validation of candidate host DNA methylation markers for detection of cervical precancer and cancer. Int J Cancer. 2017;141(4):701-710

30. Kremer WW, Steenbergen R, Heideman D, et al. The use of host cell DNA methylation analysis in the detection and management of women with advanced cervical intraepithelial neoplasia: a review. BJOG. 2021;128(3):504-514.

31. De Vuyst H, Franceschi S, Plummer M, et al. Methylation levels of CADM1, MAL, and MIR124-2 in cervical scrapes for triage of HIV-infected, high-risk HPV-positive women in Kenya. J Acquir Immune Defic Syndr. 2015;70:311–8.

32. Van Zummeren M, Kremer WW, Van Aardt MC, et al. Selection of women at risk for cervical cancer in an HIV-infected South African population. AIDS. 2017;31:1945–53.

For peer review only - http://bmjopen.bmj.com/site/about/guidelines.xhtml

33. Dankai W, Khunamornpong S, Siriaunkgul S, et al. Role of genomic DNA methylation in detection of cytologic and histologic abnormalities in high risk HPV infected women. PloS One. 2019;14(1):e0210289

34. Vasiljevic N, Scibior-Bentkowska D, Brentnall AR, et al. Credentialing of DNA methylation assays for human genes as diagnostic biomarkers of cervical intraepithelial neoplasia in high-risk HPV positive women. Gynecol Oncol. 2014;132:709–14.

35. Salta S, Maia-Moço L, Estev~aoPereira H, et al. Performance of DNA methylation-based biomarkers in the cervical cancer screening program of northern Portugal: A feasibility study. Int J Cancer. 2021;149(11):1916-1925.

36. Verhoef VMJ, Bosgraaf RP, van Kemenade FJ, et al. Triage by methylation-marker testing versus cytology in women who test HPV-positive on self-collected cervicovaginal specimens (PROHTECT-3): a randomised controlled non-inferiority trial. Lancet Oncol. 2014;15(3):315–322.

37. De Strooper LMA, Verhoef VMJ, Berkhof J, et al. Validation of the FAM19A4/mir124-2 DNA methylation test for both lavage- and brush-based self-samples to detect cervical (pre)cancer in HPV positive. Gynecol Oncol. 2016;141:341–347.

38. Luttmer R, De Strooper LM, Dijkstra MG, et al. FAM19A4 methylation analysis in self samples compared with cervical scrapes for detecting cervical (pre)cancer in HPV-positive women. Br J Cancer. 2016;115:579–87

39. Del Mistro A, Frayle H, Rizzi M, et al. Methylation analysis and HPV genotyping of self-collected cervical samples from women not responding to screening invitation and review of the literature. PloS ONE. 2017;12(3):e0172226

For peer review only - http://bmjopen.bmj.com/site/about/guidelines.xhtml

40. Verhoef VMJ, Heideman DAM, van Kemenade FJ, et al. Methylation marker analysis and HPV16/18 genotyping in high-risk HPV positive self-sampled specimens to identify women with high grade CIN or cervical cancer. Gynecol Oncol. 2014;135(1):58–63.

41. Leeman A, Del Pino M, Marimon L, et al. Reliable Identification of Women With CIN3+ Using hrHPV Genotyping and Methylation Markers in a Cytology-Screened Referral Population. Int J Cancer. 2019; 144:160–8.

42. Zhang L, Zhao X, Hu S, et al. Triage performance and predictive value of the human gene methylation panel among women positive on self-collected HPV test: Results from a prospective cohort study. Int J Cancer. 2022;151(6):878-887

43. Klischke L, von Ehr J, Kohls F, et al. Performance of a six-methylation-marker assay on self-collected cervical samples – A feasibility study. J Virol Methods. 2021;295:114219.

44. Verlaat W, Snoek BC, Heideman DAM, et al. Identification and Validation of a 3-Gene Methylation Classifier for HPV-Based Cervical Screening on Self-Samples. Clin Cancer Res. 2018;24(14):3456-3464.

For peer review only - http://bmjopen.bmj.com/site/about/guidelines.xhtml

Figure 1. Percentage of DNA methylation of gene *CADM1, MAL* and *miR124-2* according to cytological grade in oncogenic HPV positive clinician-collected cervical samples (Cervical) in the top panel and self-collected vaginal samples (Vaginal) in the bottom panel. Comparisons were assessed by the nonparametric Wilcoxon test, with whiskers corresponding to the first and third quartiles (the 25th and 75th percentiles). Additional analysis for overall significance by Kruskal-Wallis also is showed in the left side of each graph.

For peer review only

Figure 2**.** Receiver operating characteristic (ROC) curve values of the performance of DNA methylation of *CADM1, MAL* and *miR124-2* for distinguishing HSIL from LSIL/normal, and SCC from LSIL/normal, stratified by type; clinician-collected cervical samples (black bars) or self-collected vaginal samples (grey bars).

For peer review only

For peer review only - http://bmjopen.bmj.com/site/about/guidelines.xhtml

Figure 3. Percentage methylation of gene *CADM1, MAL* and *miR124-2* comparing paired clinician-collected cervical samples (Cervical) and self-collected vaginal samples (Vaginal). Comparisons assessed by Wilcoxon signed rank test, with whiskers corresponding to the first and third quartiles (the 25th and 75th percentiles).

For peer review only

For peer review only - http://bmjopen.bmj.com/site/about/guidelines.xhtml

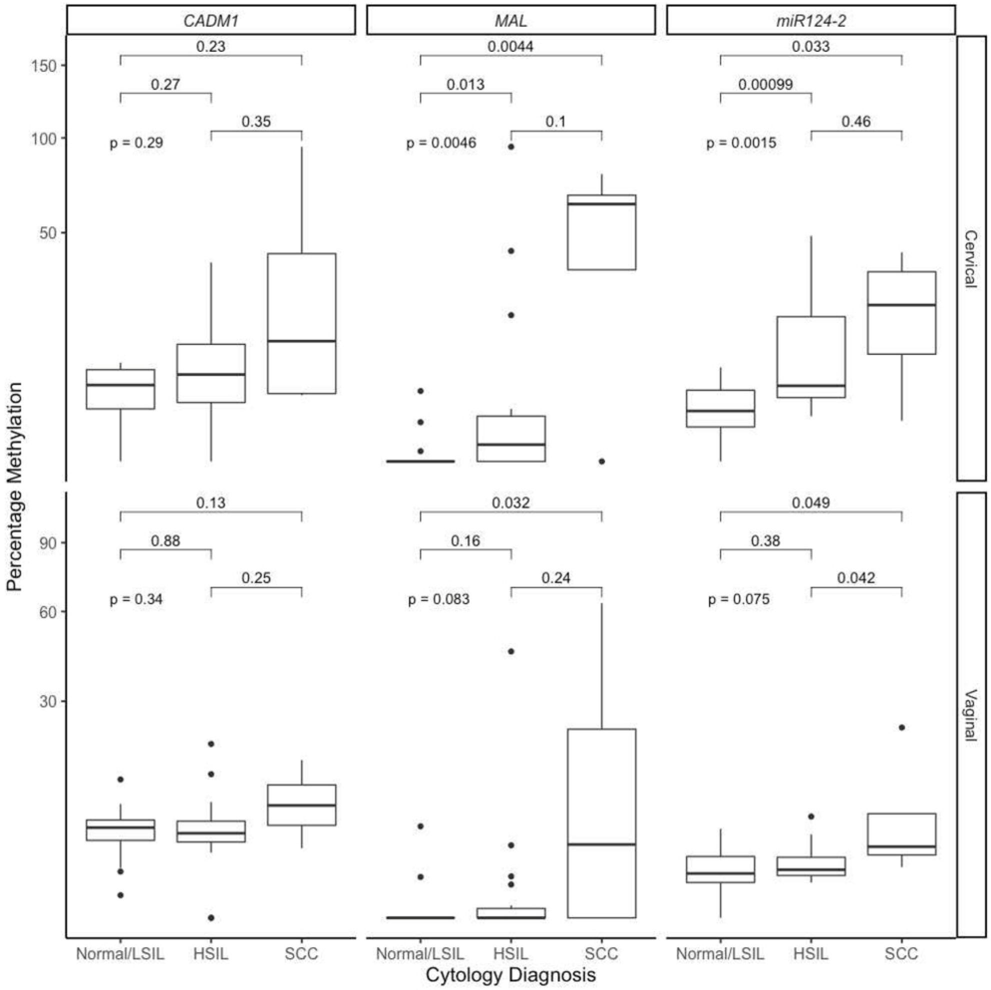

Figure 1. Percentage of DNA methylation of gene CADM1, MAL and miR124-2 according to cytological grade in oncogenic HPV positive clinician-collected cervical samples (Cervical) in the top panel and self-collected vaginal samples (Vaginal) in the bottom panel. Comparisons were assessed by the nonparametric Wilcoxon test, with whiskers corresponding to the first and third quartiles (the 25th and 75th percentiles). Additional analysis for overall significance by Kruskal-Wallis also is showed in the left side of each graph.

190x190mm (133 x 133 DPI)

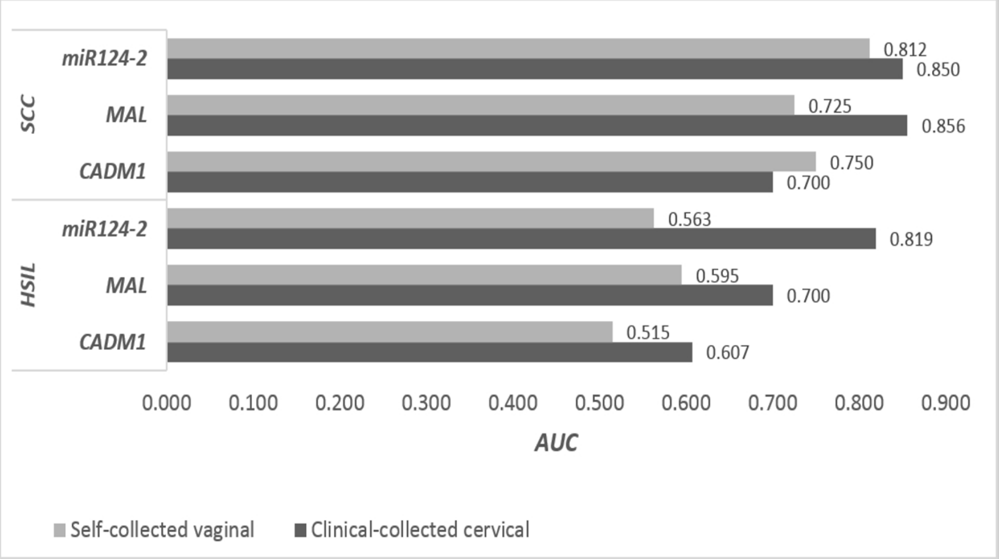

Figure 2. Receiver operating characteristic (ROC) curve values of the performance of DNA methylation of CADM1, MAL and miR124-2 for distinguishing HSIL from LSIL/normal, and SCC from LSIL/normal, stratified by type; clinician-collected cervical samples (black bars) or self-collected vaginal samples (grey bars).

295x142mm (117 x 137 DPI)

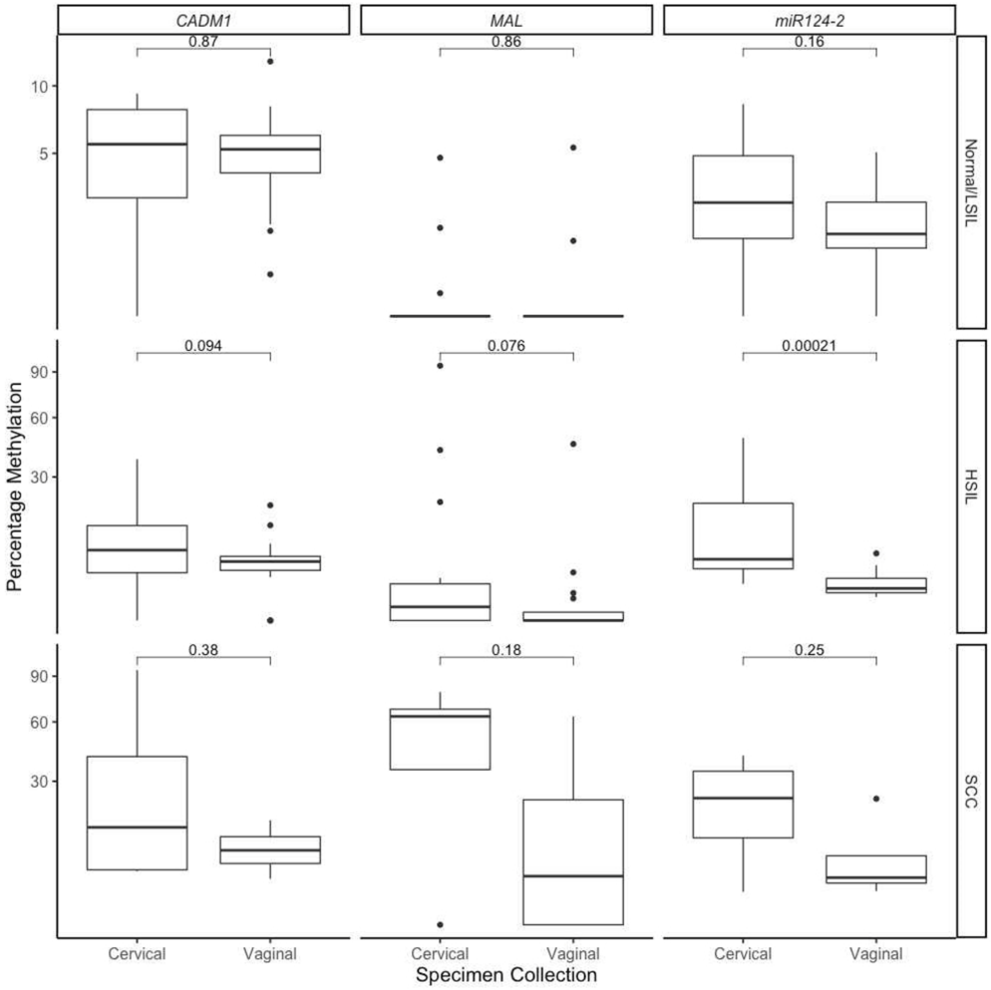

Figure 3. Percentage methylation of gene CADM1, MAL and miR124-2 comparing paired clinician-collected cervical samples (Cervical) and self-collected vaginal samples (Vaginal). Comparisons assessed by Wilcoxon signed rank test, with whiskers corresponding to the first and third quartiles (the 25th and 75th percentiles).

190x190mm (133 x 133 DPI)

Reporting checklist for diagnostic test accuracy study.

Based on the STARD guidelines.

Instructions to authors

Complete this checklist by entering the page numbers from your manuscript where readers will find each of the items listed below.

Your article may not currently address all the items on the checklist. Please modify your text to include the missing information. If you are certain that an item does not apply, please write "n/a" and provide a short explanation.

Upload your completed checklist as an extra file when you submit to a journal.

In your methods section, say that you used the STARDreporting guidelines, and cite them as:

Bossuyt PM, Reitsma JB, Bruns DE, Gatsonis CA, Glasziou PP, Irwig L, LijmerJG Moher D, Rennie D, de Vet HCW, Kressel HY, Rifai N, Golub RM, Altman DG, Hooft L, Korevaar DA, Cohen JF, For the STARD Group. STARD 2015: An Updated List of Essential Items for Reporting Diagnostic Accuracy Studies.

		Reporting Item	Page Number
**Title or abstract**			
None	#1	Identification as a study of diagnostic accuracy using at least one measure of accuracy (such	3

For peer review only

		as sensitivity, specificity, predictive values, or AUC)	

Abstract

None	#2	Structured summary of study design, methods, results, and conclusions (for specific guidance, see STARD for Abstracts https://www.equator-network.org/reporting-guidelines/stard-abstracts/)	3

Introduction

None	#3	Scientific and clinical background, including the intended use and clinical role of the index test	6-7
None	#4	Study objectives and hypotheses	8

Methods

Study design	#5	Whether data collection was planned before the index test and reference standard were performed (prospective study) or after (retrospective study)	9-10
Participants	#6	Eligibility criteria	9-10

For peer review only

Participants	#7	On what basis potentially eligible participants were identified (such as symptoms, results from previous tests, inclusion in registry)	9-10
Participants	#8	Where and when potentially eligible participants were identified (setting, location and dates)	9,11
Participants	#9	Whether participants formed a consecutive, random or convenience series	9,11
Test methods	#10	Index and reference tests in sufficient detail to allow replication	9-13
Test methods	#11	Rationale for choosing the reference standard (if alternatives exist)	10
Test methods	#12	Definition of and rationale for test positivity cut-offs or result categories of the index and reference tests, distinguishing pre-specified from exploratory	10,12-13
Test methods	#13	Whether clinical information and reference standard results were available to the performers / readers of the index test; Whether clinical information and index test results were available to the assessors of the reference standard	11

For peer review only

Analysis	#14	Methods for estimating or comparing measures of diagnostic accuracy	12-13
Analysis	#15	How indeterminate index test or reference standard results were handled	12-13
Analysis	#16	How missing data on the index test and reference standard were handled	12
Analysis	#17	Any analyses of variability in diagnostic accuracy, distinguishing pre-specified from exploratory	10,13
Analysis	#18	Intended sample size and how it was determined	9,11
**Results**			
Participants	#19	Flow of participants, using a diagram	NA<br>This is an exploratory study. The flow of participants was outlined in detail in the results section, and thus a diagram was not deemed necessary.
Participants	#20	Baseline demographic and clinical characteristics of participants	15

For peer review only

Participants	#21	Distribution of severity of disease in those with the target condition, and distribution of alternative diagnoses in those without the target condition	15
Participants	#22	Time interval and any clinical interventions between index test and reference standard	11,15
Test results	#23	Cross tabulation of the index test results (or their distribution) by the results of the reference standard	15,16,22
Test results	#24	Estimates of diagnostic accuracy and their precision (such as 95% confidence intervals)	16-21
Test results	#25	Any adverse events from performing the index test or the reference standard	15
**Discussion**			
None	#26	Study limitations, including sources of potential bias, statistical uncertainty, and generalisability	26
None	#27	Implications for practice, including the intended use and clinical role of the index test	26-27
**Other information**			
None	#28	Registration number and name of registry	9

For peer review only

None	#29	Where the full study protocol can be accessed	9
None	#30	Sources of funding and other support; role of funders	2

The STARD checklist is distributed under the terms of the Creative Commons Attribution License CC-BY. This checklist was completed on 10. October 2023 using https://www.goodreports.org/, a tool made by the EQUATOR Network in collaboration with Penelope.ai

For peer review only

BMJ Open

Performance of CADM1, MAL and miR124-2 methylation as triage markers for early detection of cervical cancer in self- and clinician-collected samples: a case-control observational study in Papua New Guinea

Journal:	*BMJ Open*
Manuscript ID	bmjopen-2023-081282.R1
Article Type:	Original research
Date Submitted by the Author:	07-Mar-2024
Complete List of Authors:	Molano, Monica; The Royal Women's Hospital, Centre for Women's Infectious Diseases; Murdoch Children's Research Institute<br>Machalek, Dorothy; University of New South Wales, Kirby Institute; The Royal Women's Hospital, Centre for Women's Infectious Diseases<br>Tan, Grace; Australian Centre for the Prevention of Cervical Cancer<br>Garland, Suzanne; The Royal Women's Hospital; The University of Melbourne, Department of Obstetrics, Gynaecology and Newborn Health<br>Balgovind, Prisha; The Royal Women's Hospital; Murdoch Children's Research Institute<br>Haqshenas, Gholamreza; The Royal Women's Hospital; The University of Melbourne, Department of Obstetrics, Gynaecology and Newborn Health<br>Munnull, Gloria; Papua New Guinea Institute of Medical Research<br>Phillips, Samuel; The Royal Women's Hospital; Murdoch Children's Research Institute<br>Badman, Steven; University of New South Wales, Kirby Insitute<br>Bolnga, John ; Modilon General Hospital, Obstetrics and Gynaecology ; Papua New Guinea Institute of Medical Research, Sexual and Reproductive Health<br>Cornall, Alyssa; The Royal Women's Hospital; Murdoch Children's Research Institute<br>Gabuzzi, Josephine; Papua New Guinea Institute of Medical Research<br>Kombati, Zure; Mount Hagen General Hospital, Tininga Clinic<br>Brotherton, Julia; Australian Centre for the Prevention of Cervical Cancer; The University of Melbourne, School of Population Global Health<br>Saville, Marion; Australian Centre for the Prevention of Cervical Cancer<br>Hawkes, David; Australian Centre for the Prevention of Cervical Cancer<br>Kaldor, John; University of New South Wales, The Kirby Institute<br>Toliman, Pamela; University of New South Wales, Kirby Institute -Faculty of Medicine<br>Vallely, Andrew; Papua New Guinea Institute of Medical Research, Sexual and Reproductive Health Unit; University of New South Wales, The Kirby Institute<br>Murray , Gerald L ; The University of Melbourne - Parkville Campus, Obstetrics, Gynaecology and Newborn Health; The Royal Women's Hospital, Centre for Women's Infectious Diseases
**Primary Subject Heading**:	Obstetrics and gynaecology

Secondary Subject Heading:	Oncology, Infectious diseases, Diagnostics
Keywords:	Molecular diagnostics < INFECTIOUS DISEASES, MOLECULAR BIOLOGY, Oncogenes < ONCOLOGY, Human Papillomavirus Viruses

SCHOLARONE™ Manuscripts

I, the Submitting Author has the right to grant and does grant on behalf of all authors of the Work (as defined in the below author licence), an exclusive licence and/or a non-exclusive licence for contributions from authors who are: i) UK Crown employees; ii) where BMJ has agreed a CC-BY licence shall apply, and/or iii) in accordance with the terms applicable for US Federal Government officers or employees acting as part of their official duties; on a worldwide, perpetual, irrevocable, royalty-free basis to BMJ Publishing Group Ltd ("BMJ") its licensees and where the relevant Journal is co-owned by BMJ to the co-owners of the Journal, to publish the Work in this journal and any other BMJ products and to exploit all rights, as set out in our licence.

The Submitting Author accepts and understands that any supply made under these terms is made by BMJ to the Submitting Author unless you are acting as an employee on behalf of your employer or a postgraduate student of an affiliated institution which is paying any applicable article publishing charge ("APC") for Open Access articles. Where the Submitting Author wishes to make the Work available on an Open Access basis (and intends to pay the relevant APC), the terms of reuse of such Open Access shall be governed by a Creative Commons licence – details of these licences and which Creative Commons licence will apply to this Work are set out in our licence referred to above.

Other than as permitted in any relevant BMJ Author's Self Archiving Policies, I confirm this Work has not been accepted for publication elsewhere, is not being considered for publication elsewhere and does not duplicate material already published. I confirm all authors consent to publication of this Work and authorise the granting of this licence.

For peer review only

Performance of *CADM1, MAL* and *miR124-2* methylation as triage markers for early detection of cervical cancer in self- and clinician-collected samples: a case-control observational study in Papua New Guinea

Monica Molano[1,2], Dorothy A Machalek[1,3], Grace Tan[5], Suzanne M Garland[1,2,4], Samuel Phillips[1,2], Prisha Balgovind[1,2], Steven G Badman[3], Gholamreza Haqshenas[1,2,4], John Bolnga[6], Alyssa Cornall[1,2], Josephine Gabuzzi[6], Zure Kombati[7], Gloria Munnull[8], Julia M Brotherton[5], Marion Saville[5], David Hawkes[5], John M Kaldor[3], Pamela J Toliman[3], Andrew J Vallely[3]*, Gerald L. Murray[1,2,4]*

1. Centre for Women's Infectious Diseases, The Royal Women's Hospital, Melbourne, Victoria, Australia

2. Murdoch Children's Research Institute, Melbourne, Vic, Australia

3. The Kirby Institute, University of New South Wales, Sydney, NSW, Australia

4. Department of Obstetrics and Gynaecology, University of Melbourne, Parkville, Victoria, Australia

5. Australian Centre for the Prevention of Cervical Cancer, Melbourne, Victoria, Australia

6. Papua New Guinea Institute of Medical Research, Goroka, Papua New Guinea ; Department of Obstetrics and Gynaecology, Modilon General Hospital, Madang, Papua New Guinea.

7. Department of Pathology, Mt Hagen Provincial Hospital, WHP 281, Papua New Guinea

8. Papua New Guinea Institute of Medical Research, Goroka, Papua New Guinea; Department of Obstetrics and Gynaecology, Mt Hagen Provincial Hospital, Mount Hagen, Papua New Guinea.

*Indicates co-senior authors

Running title: DNA methylation-based triage markers for cervical cancer

Corresponding author:

For peer review only

Monica Molano, PhD

Telephone +61 3 8345 3691

Fax +61 3 9347 8235

Email Monica.MolanoLuque@thewomens.org.au

Disclosure of Potential Conflicts of Interest:

AJV, JG, JB, MG, PJT, SGB, JMK have received subsidised test kits for research from Cepheid. MS, JMB, GT, DH have received donated test kits for research from Abbott, BD, Cepheid, Hologic, Qiagen, Roche, and Seegene. AJV and MS jointly lead the *Elimination of Cervical Cancer in the Western Pacific (ECCWP)* program with philanthropic funding support from the Minderoo Foundation and the Frazer Family Foundation; and equipment, tests and consumables donated by Cepheid for HPV-based cervical screening in Papua New Guinea and Vanuatu. SMG is a member of the Global Advisory Board for HPV vaccines Merck and has led investigator-initiated grants from Merck on HPV in young women. MM, DAM, SP, PB, GH, ZK, GLM, declare no conflicting interests.

Grant Support: This work was funded through the National Health and Medical Research Council, Australia, (NHMRC) grants 1013209 and 1104938 (A.J.V), Government of Papua New Guinea (ICRAS 297/1), the NHMRC Centre for Research Excellence in Cervical Cancer Control APP1135172 (JMB, MS, AV, SMG, JMK, DAM, DH) and NHMRC Investigator Grant APP1197951 (S.M.G).

For peer review only - http://bmjopen.bmj.com/site/about/guidelines.xhtml

Abstract

Objective: WHO recommends human papillomavirus (HPV) testing for cervical screening, with triage of high-risk human papillomavirus (hrHPV) positive women. However, there are limitations to effective triage for low resource, high burden settings, such as Papua New Guinea. In this exploratory study we assessed the performance of host methylation as triage tools for predicting high-grade squamous intraepithelial lesions (HSIL) in self- and clinician-collected samples.

Design: case-control observational study

Setting: Provincial hospital, same day cervical screen-and-treat trial, Papua New Guinea

Participants: 44 hrHPV+ women, with paired self/clinician-collected samples [4 squamous cell carcinomas (SCC), 19 HSIL, 4 low-grade squamous intraepithelial lesions (LSIL), 17 normal]

Primary and secondary outcome measures: Methylation levels of *CADM1, MAL* and *miR124-2* analysed by methylation-specific PCRs against the clinical endpoint of HSIL or SCC (HSIL+) measured using liquid-based-cytology (LBC)/p16-Ki67 stain.

Results: In clinician-collected samples, *MAL* and *miR124-2* methylation levels were significantly higher with increasing grade of disease ($p=0.0046$ and $p<0.0015$ respectively). *miR124-2* was the best predictor of HSIL (AUC of 0.819), whilst *MAL* of SCC (0.856). In self-collected samples, *MAL* best predicted HSIL (0.595), whilst *miR124-2* SCC (0.812). Combined *miR124-2/MAL* methylation yielded sensitivity and specificity for HSIL+ of 90.5% (95%CI: 69.6-98.8) and 70% (45.7-88.1) respectively in clinician-collected samples, and 81.8% (59.7-94.8) and 47.6% (25.7-70.2) respectively in self-collected samples. *miR124-2/MAL* plus HPV16/HPV18 improved sensitivity for HSIL+ (95.2%, 76.2-99.9), but decreased specificity (55.0%, 31.5-76.9).

For peer review only - http://bmjopen.bmj.com/site/about/guidelines.xhtml

Conclusion: *miR124-2/MAL* methylation is a potential triage strategy for the detection of HSIL/SCC in LMIC.

Key words:

DNA Methylation, Cervical Cancer, Human papillomavirus, Diagnostic test, Epigenetics, Molecular diagnostics, Molecular Biology, Oncogenes.

Data availability statement: Limited data may be available on reasonable request from the corresponding author, subject to ethical approval.

Strengths and Limitations of this study

- This study uniquely investigated host methylation markers in self-collected samples in a low-middle income country
- A direct comparison of self-collected samples and paired clinician-collected samples was possible, thus identifying different performance and optimal cut-off values by sample type
- Study design allowed exploration of the clinical performance of extended genotyping (HPV16/18/31/33/45/52/58) combined with host methylation analysis for the detection of HSIL
- A small sample size led to broad confidence intervals
- Due to limited specialist staff and infrastructure, liquid-based cytology was the diagnostic reference standard rather than a histological end point

For peer review only - http://bmjopen.bmj.com/site/about/guidelines.xhtml

For peer review only

Introduction

Cervical cancer is the fourth most frequently diagnosed cancer and the fourth leading cause of cancer-related death in women, with an estimated 604,000 new cases and 342,000 deaths worldwide in 2020 (1). Age standardised rates are disproportionally higher in low- and middle-income countries (LMIC) than in high-income countries (18.8 vs 11.3 per 100,000 for incidence: 12.4 vs 5.2 per 100,000 for mortality), primarily due to disparities in access to effective prevention, screening and treatment strategies (2-5). Infection with high-risk HPV types (hrHPV) may cause cervical abnormalities graded histologically as cervical intraepithelial neoplasia (CIN, grades 1-3) or cancer (principally SCC or adenocarcinoma), or graded cytologically (using exfoliated cells from the cervix) as LSIL, HSIL or SCC (6).

To achieve cervical cancer elimination (5), many countries are transitioning their cervical screening programs to HPV nucleic acid-based testing, which is more sensitive than cytology for detecting underlying high-grade disease. Furthermore, sample collection can be self-administered, which has been shown to increase screening participation in many settings (6-11). Another important advantage is that nucleic acid amplification testing and subsequent treatment can be performed at point of care, greatly simplifying program delivery, particularly in low resource settings. These advances led the WHO, in 2021, to endorse HPV screen-and-treat guidelines for women in LMIC (6).

For peer review only - http://bmjopen.bmj.com/site/about/guidelines.xhtml

An important limitation of HPV testing is that it suffers from a lower specificity than cytology, leading to concern that the treatment of all hrHPV+ women would result in substantial overtreatment (12). Hence, a second (triage) test is recommended to identify hrHPV+ women at highest risk of having underlying disease requiring treatment. The triage technologies most used are cytology, partial genotyping for HPV16 or 18, and in many low resource settings, visual inspection using acetic acid or Lugol's iodine (12). However, these triage approaches present challenges for sensitivity and/or specificity in LMIC settings (8, 12).

Molecular triage tests are advantageous over current methods as they are less subjective and could be performed on multiple specimen types, including self-samples. In particular, aberrant host gene DNA methylation patterns in promoter regions of some genes has shown promise as a triage marker in cervical screening in high income countries. Aberrant methylation is pivotal in cervical carcinogenesis; it causes changes in gene expression, faulty condensation, and chromosomal instability (13,14). Increased DNA methylation has been associated with hrHPV persistence (13) and correlates with increasing severity of cervical disease (14, 15). DNA methylation markers such as *CADM1, MAL* and *miR124-2* have shown good performance for the detection of CIN2 or worse (CIN2+) among hrHPV+ women (14, 16, 17). However, data on their performance has been based on studies conducted in high income settings and among highly screened populations (18). There is currently no data on the performance of these markers among LMIC populations by using self-collected vaginal samples, nor in settings where HPV-based screening-and- same day-treatment strategies are used.

For peer review only - http://bmjopen.bmj.com/site/about/guidelines.xhtml

Papua New Guinea has a high burden of cervical cancer, with age standardized incidence and mortality rates of 29.2 and 19.1 per 100,000, respectively, making it the second most common cancer among Papua New Guinean women (3). A trial study (HPV-STAT) performed in women from this country evaluated the clinical performance, treatment completion rates, adverse events profile, and acceptability of a fully integrated screening strategy, comprising point-of-care HPV test of self-collected specimens and same-day thermal ablation (19). This trial supported the introduction of HPV screening and same-day treatment for the control of cervical cancer in LMIC (19). In the current study, we explored the performance of *CADM1, MAL* and *miR124-2* DNA methylation markers alone and in combination with HPV16/18 and extended genotyping (HPV16/18/31/33/45/52/58) as a triage strategy for the detection of HSIL+ among HPV+ women that participated in this cervical screening trial. A direct comparison was also performed for each methylation marker, between paired self- and clinician-collected samples.

Materials and Methods

Study population and design of the trial

The exploratory study was conducted as a sub-study of HPV-STAT ('HPV self-collect, test and-same-day-treat') a prospective, single-arm intervention trial among women attending cervical screening services at two clinical sites in Papua New Guinea. The trial is registered with ISRCTN, ISRCTN13476702 (https://www.isrctn.com/editorial/retrieveFile/3ed9173e-cce5-4158-a586-577775f0cbdd/35731). Study design, recruitment and protocols have been described (19). Briefly, between June 5, 2018, and Jan 6, 2020, 4285 women aged 30 to 59 years attending clinics for routine cervical screening at Modilon Hospital (Madang Province) and Mt Hagen

For peer review only - http://bmjopen.bmj.com/site/about/guidelines.xhtml

General Hospital (Western Highlands Province), who meet the inclusion criteria were invited to participate. Women who were pregnant or who had given birth in the past 6 weeks, or women who had a history of cervical cancer or hysterectomy, were excluded (19). Those women able to provide written informed consent were eligible to participate, enrolled sequentially and collected a vaginal specimen using a cytobrush ("Just for Me", Preventative Oncology International, Cleveland Heights, Ohio), which was placed into a 20 ml ThinPrep vial (Hologic, Marlborough, MA) (19).

Point Of Care HPV testing and follow-up assessment

For self-collected vaginal specimens, a 1 ml aliquot was tested for the presence of oncogenic HPV genotypes using the Xpert HPV Test (GeneXpert; Cepheid, Sunnyvale, CA, USA) as per the manufacturer's instructions. Results were reported as 'HPV16', 'HPV18/45', and 'Other HPV' (a summary result for HPV31, 33, 35, 39, 51, 52, 56, 58, 59, 66 and 68) and were provided to women before midday to allow sufficient time for same-day counselling, pelvic examination and treatment/referral as indicated (19). All women with a negative HPV test were informed of their results and advised to return to the clinic for HPV-based screening in five years.

hrHPV+ women provided a cervical specimen collected by a clinician using a Cervex-Brush Combi (Rovers Medical Devices, Oss, The Netherlands), placed in a 20 ml ThinPrep vial, and stored at 4°C prior to shipment to the Australian Centre for the Prevention of Cervical Cancer in Melbourne, Australia for liquid-based cytology (LBC) and p16/Ki67 dual stain cytology. A 15% random sample of HPV negative women were also asked to provide a clinician-collected cervical specimen for LBC, as above.

Cytological assessment

For peer review only - http://bmjopen.bmj.com/site/about/guidelines.xhtml

LBC was carried out in accordance with established laboratory procedures at the Australian Centre for the Prevention of Cervical Cancer. Slides were independently assessed by two cytologists blinded to HPV test results. Where both cytologists agreed on a diagnosis of HSIL or HSIL+, a final diagnosis was recorded. If the assessment differed, dual p16/Ki-67 dual stain was carried out by using CINTec PLUS Cytology (Roche Diagnostics, Pleasanton, USA) to make a final diagnosis (19). The primary clinical endpoint was then HSIL or SCC (HSIL+) using LBC/p16-Ki67 stain. LBC was accepted as a reference standard rather than histology, which is the gold reference in high-resource settings. It was not feasible from a staffing and logistical perspective to provide colposcopy or to collect cervical biopsies for histological examination in Papua New Guinea.

Participants in the exploratory study and design

The sub-study included 44 paired cervical and vaginal samples from women participating in the trial at Mt Hagen General Hospital (Mount Hagen, Western Highlands Province). This included all 23 hrHPV+ HSIL+ (19 HSIL and 4 SCC) cases identified on LBC by the end of 2018 and 21 randomly selected hrHPV+ normal/LSIL samples (17 normal LBC and 4 LSIL). Sample size selection was driven by a published study by Li et al., 2015 that showed through simulation studies and real data from 2 studies downloaded from the NCBI Gene Expression Omnibus that at least 12 specimens in each group is needed to detect truly differential DNA methylation with enough power (≥ 80%), reproducible data and consistence when using different statistical methods (20). Molecular biologists and technicians were blinded to point of care HPV and clinical diagnosis.

DNA Extraction and HPV specific typing

For peer review only - http://bmjopen.bmj.com/site/about/guidelines.xhtml

DNA was extracted as described previously, quantitated by Qubit® Fluorometer (Life technologies, California, USA), and assessed for integrity by quantitative PCR amplification of a 260 base-pair product of the human beta-globin gene (21). In addition, DNA extracted from cervical cell line SiHa (1-2 copies of HPV16 per cell, ATCC Cat# HTB-35, RRID:CVCL_0032; American Type Culture Collection (ATCC), Manassas, Virginia, USA; 100 ng) was used as positive control for methylation analysis. This DNA was confirmed to have only HPV16 by HPV typing for 28 HPV types (Anyplex II HPV28 Detection, Seegene, Seoul, South Korea).

HPV genotyping was performed using the Anyplex II HR HPV Detection multiplex assay (Seegene, Seoul, South Korea), which detects 14 oncogenic HPV genotypes (16, 18, 31, 33, 35, 39, 45, 51, 52, 56, 58, 59, 66, 68) and an internal control, according to manufacturer's recommendations.

Bisulphite DNA modification and Quantitative methylation-specific PCR for CADM1, MAL and miR124 genes

DNA from the paired samples (1-100ng) and control (SiHa, 100 ng) were bisulphite treated using Methylamp DNA modification Kit (Epigentek, Brooklyn, NY, USA) as per the manufacturer's instructions. Modified DNA was eluted in 40µL of the Methylamp elution buffer. Samples without the adequate concentration were excluded from the bisulphite modification and methylation analysis.

Quantitative methylation-specific PCR (qMSP) targeting CpG sites in promoter regions of *CADM1* (promoter region M9), *MAL* (promoter region M1) and *miR124-2* (promoter region 2)

For peer review only - http://bmjopen.bmj.com/site/about/guidelines.xhtml

were performed as previously described (14), with minor modifications. Due to the use of a different platform (Light Cycler 480 II, Roche), validations of the qPCRs were performed utilising duplicated dilution series of SiHa cell line (intra and inter assays of reproducibility) and by using a training panel of samples as reported previously (21). As a reference, a qMSP for the housekeeping gene b-actin *(ACTB*) was performed. Samples with Ct values for *ACTB* of >34 were considered *ACTB* negative as this indicated poor sample quality due to insufficient DNA or inadequate bisulphite conversion. Target DNA methylation levels were normalized to the reference gene as described previously (21).

Statistical Analysis

The percentage of methylation was calculated as described previously (22). Differences in the median percentage methylation by disease grade (SCC, HSIL, LSIL/normal) were visualised using box and whisker plots and compared using Wilcoxon test (between groups) and Kruskal-Wallis (overall analysis) (20, 21). Area under the receiver operating characteristic curve (AUC) was used to assess the ability of the methylated genes to classify HSIL or SCC, providing two main outcomes: the diagnostic accuracy of the test and the optimal cut off point value for dichotomization of the methylation test (23). The optimal cut off-point was calculated by using the maximum sum of sensitivity and specificity (24). Sensitivity [number of correct positives (i.e. positive for at least one marker)/ number of reference assay positives] and specificity (number of correct negatives/number of reference assay negatives) were determined for the following triage strategies: (I) single gene methylation analysis, (II) combination of methylation markers, (III) HPV 16 genotyping, (IV) combination of HPV16/18 genotyping, (V) combination of HPV 16/18/31/33/45/52/58 genotyping, (VI) HPV16/18 with

For peer review only - http://bmjopen.bmj.com/site/about/guidelines.xhtml

single and combined methylation analysis, and (VII) HPV 16/18/31/33/45/52/58 with single and combined methylation analysis.

Median percentage methylation for each marker was compared for self- and clinician-collected vaginal paired specimens using the Wilcoxon signed rank test. The vaginal specimen was considered the test while the cervical specimen acted as the non-reference standard. Agreements between DNA methylation results from cervical and vaginal specimens were determined using the optimal cut off point established for each gene by calculating positive percent agreement (PPA), negative percent agreement (NPA) and overall percent agreement (OPA) as per recommendations by the Food and Drug Administration, USA (25).

The results were analysed by using XLSTAT and the statistical platform R studio (v4.0.1) and programs ggplots2 (v3.3.2), ggpubr (v0.4), pROC (v1.16.2) and cutpointr (v1.1.1) (21, 24).

We used the STARD reporting guidelines for reporting diagnostic accuracy studies (26).

Ethical considerations

Approval of the trial was provided by the Medical Research Advisory Committee (MRAC) of the Papua New Guinea National Department of Health (approval number 17.36), the Institutional Review Board of the Papua New Guinea Institute of Medical Research (IRB 1712), and the Human Research Ethics Committee (HREC) of UNSW Australia (approval number HC17631). Written informed consent was obtained from all participants prior to enrolment.

Patient and Public Involvement

For peer review only - http://bmjopen.bmj.com/site/about/guidelines.xhtml

There was no patient or public involvement in study design or conduct.

For peer review only

For peer review only - http://bmjopen.bmj.com/site/about/guidelines.xhtml

Results

DNA Methylation of CADM1, MAL and miR124-2 and associations with lesion grade

Overall, 44 hrHPV+ paired cervical and vaginal samples from women participating in the trial at Mt Hagen General Hospital were used [23 cases (19 HSIL and 4 SCC 8) and 21 normal/LSIL (17 normal LBC and 4 LSIL)]. Of these samples, 41 (93.2%) clinician-collected (4 SCC, 17 HSIL, 4 LSIL and 16 normal LBC) and 43 (97.7%) self-collected samples (4 SCC, 18 HSIL, 4 LSIL and 17 normal LBC) were deemed assessable. The methylation analysis was not used for the clinical management of the women, and thus did not induce any adverse effects.

In clinician-collected samples DNA methylation of *MAL* was higher with increasing disease grade from normal/LSIL samples (1.4%; 0.8-3.1), HSIL (median 2.0%; 95%CI:0.7-20.4) and SCC (63.3%; 46.8-67.9) (p=0.0046) (Figure 1, top central panel). Methylation of *mirR124-2* was also higher with increasing disease grade from normal/LSIL samples (2.6%; 1.1-5.1), HSIL (5.6%; 3.9-20.5) and SCC (24.1%; 12.4-34.5) (p=0.0015) (Figure 1, top right panel). Methylation of *CADM1* did not differ significantly with increasing cytology grade (Figure 1, top left panel).

Using clinician-collected samples, ROC analysis showed that *miR124-2* was the best methylation marker to distinguish SCC and HSIL (from normal/LSIL), with an AUC of 0.850 and 0.819, respectively (Figure 2). Methylation of *MAL* showed higher AUC for detecting SCC (0.856) than HSIL (0.700). *CADM1* methylation showed poor performance for detecting SCC or HSIL with AUC of 0.700 and 0.607 respectively (Figure 2).

For peer review only - http://bmjopen.bmj.com/site/about/guidelines.xhtml

In self-collected samples, there was a pattern of increasing DNA methylation of *MAL* with disease grade for MAL and MiR124-2, though this did not reach significance (p=0.083, p=0.075, respectively) (Figure 1, lower centre panel). As found for clinician-collected samples, methylation of *CADM1* did not differ significantly with increasing cytology grade (Figure 1, lower left panel).

ROC analysis showed that from self-collected samples, *miR124-2* was the best methylation marker to distinguish SCC, but not HSIL, from normal/LSIL with an AUC of 0.812 and 0.563, respectively (Figure 2). Methylation of *MAL* or *CADM1* showed a lower clinical performance for SCC (AUC of 0.725 and 0.750, respectively) than *miR124-2*, and a similar performance for HSIL (AUC of 0.595 and 0.515, respectively) (Figure 2).

Diagnostic performance of DNA methylation markers for the detection of HSIL or SCC

Diagnostic performance of individual DNA methylation markers for detecting HSIL+ in clinician-collected samples showed that *miR124-2* methylation had the highest sensitivity of 81.0% (95%CI: 58.1-94.6) and *MAL* methylation the highest specificity of 90.0% (68.3-98.8) (Table 1). A combination of *miR124-2* and *MAL* showed the optimal diagnostic performance with a sensitivity of 90.5% (69.6-98.8) and specificity of 70.0% (45.7-88.1). A combination of *miR124-2/MAL* and *CADM1* showed a maximal sensitivity of 100% (95% CI 83.9-100) at the expense of a low specificity, 25% (8.7-49.1).

In self-collected samples, *miR124-2* methylation had a sensitivity of 77.3 % (95% CI 54.6-92.2) for detecting HSIL+, but much lower specificity 47.6% (25.7-70.2). *MAL* showed the best

For peer review only - http://bmjopen.bmj.com/site/about/guidelines.xhtml

specificity 90.5% (69.6-98.8) at the expense of a low sensitivity 32.0% (13.9-54.9) (Table 1). A combination of *miR124-2* and *MAL* improved the diagnostic performance with a sensitivity of 81.8% (95% CI 59.7-94.8) and specificity of 47.6% (25.7-70.2). A combination of *miR124-2/MAL* and *CADM1* increased the sensitivity to 90.9% (70.8-98.9) at the expense of a low specificity of 23.8% (8.2-47.2) (Table 1).

For peer review only

For peer review only - http://bmjopen.bmj.com/site/about/guidelines.xhtml

336 **Table 1.** Performance of host DNA methylation markers for detection of HSIL+

Sample type	Gene[a]	Percent methylation cut-off	Sensitivity % (95% CI)	Specificity % (95% CI)
Cervical, clinician-collected	*miR124-2*	3.7	81.0 (58.1-94.6)	70.0 (45.7-88.1)
	MAL	0.25	57.1 (34.0-78.2)	90.0 (68.3-98.8)
	CADM1	3.5	80.9 (58.1-94.6)	40.0 (19.1-63.9)
	miR124-2/MAL	3.7/0.25	90.5 (69.6-98.8)	70.0 (45.7-88.1)
	miR124-2/CADM1	3.7/3.5	95.2 (76.2-99.9)	25.0 (8.7-49.1)
	MAL/CADM1	0.25/3.5	95.2 (76.2-99.9)	35.0 (15.4-59.2)
	miR124-2/MAL/CADM1	3.7/0.25/3.5	100 (83.9-100)	25.0 (8.7-49.1)
Vaginal, self-collected	*miR124-2*	1.2	77.3 (54.6-92.2)	47.6 (25.7-70.2)
	MAL	0.1	32.0 (13.9-54.9)	90.5 (69.6-98.8)
	CADM1	3.9	68.2 (45.1-86.1)	33.3 (14.6-57.0)
	miR124-2/MAL	1.2/0.1	81.8 (59.7-94.8)	47.6 (25.7-70.2)
	miR124-2/CADM1	1.2/3.9	86.4 (65.1-97.1)	23.8 (8.2-47.2)
	MAL/CADM1	0.1/3.9	81.8 (59.7-94.8)	28.6 (11.3-52.2)
	miR124-2/MAL/CADM1	1.2/0.1/3.9	90.9 (70.8-98.9)	23.8 (8.2-47.2)

337 [a] Positive for at least one of the indicated host genes

338

339 Shading rows show markers or combination of markers with the best diagnostic performance and those with the maximal sensitivity for detection of HSIL+

For peer review only - http://bmjopen.bmj.com/site/about/guidelines.xhtml

Diagnostic performance of HPV genotyping combined with DNA methylation markers for the detection of HSIL or SCC

Diagnostic performance of HPV16 for detecting HSIL+ in clinician-collected samples showed -a sensitivity of 52.2% (95% CI 30.6-73.2) and specificity of 81.0% (95% CI 58.1-94.6) (Table 2). HPV16 and HPV18 combined showed a sensitivity of 60.9% (95% CI 38.5-80.3) and specificity of 81.0% (95% CI 58.1-94.6). A combination of *miR124-2/MAL* methylation markers and HPV16/18 genotyping showed a satisfactory diagnostic performance with a sensitivity 95.2% (95% CI 76.2-99.9) and specificity of 55% (95% CI 31.5-76.9). A combination of *MAL/CADM1* and HPV16/18 genotyping showed a maximal sensitivity of 100% (95% CI 85.9-100) at the expense of a specificity of 30% (11.9-54.3). Using HPV16/18/31/33/45/52/58 genotyping, either as a single marker or combined with different methylation markers, showed high sensitivities but at the expense of low specificities (Table 2).

For self-collected samples, detection of individual and combined HPV genotypes had the same diagnostic performance as seen in clinician-collected samples (Table 2). A combination of *miR124-2 or miR124/MAL and* HPV16/HPV18 showed similar sensitivity found for clinician-collected samples of 95.5% (95%CI:77.2-99.9), but reduced specificity (33.3%, 95%CI: 14.6-50.7). A combination of *miR124-2/CADM1* and HPV16/18 genotyping showed a maximal sensitivity of 100% (95% CI 84.6-100) and specificity of 23.8% (8.2-47.2). Adding HPV16/18/31/33/45/52/58 genotyping combined with the different methylation markers showed high sensitivities at expenses of a very low specificities (Table 2).

For peer review only - http://bmjopen.bmj.com/site/about/guidelines.xhtml

361 ***Table 2.*** Performance of HPV genotyping combined with methylation markers for the detection of HSIL+

	Combined HPV/methylation detection for decision making				
**Sample type**	**HPV genotype [a]**	**Host Gene[b]**	**Percent methylation cut-off**	**Sensitivity % (95% CI)**	**Specificity (95%CI)**
Cervical, clinician-collected	16	NA	NA	52.2 (30.6-73.2)	81.0 (58.1-94.6)
	16,18	NA	NA	60.9 (38.5-80.3)	81.0 (58.1-94.6)
	16,18,31,33,45,52,58	NA	NA	95.7 (78.1-99.9)	33.3 (14.6-56.9)
	16,18	*miR124-2*	3.7	85.7 (63.7-97.0)	55.0 (31.5-76.9)
	16,18,31,33,45,52,58	*miR124-2*	3.7	95.2 (76-2-99.9)	25.0 (8.7-49.1)
	16,18	*MAL*	0.25	76.2 (52.8-91.8)	70.0 (45.7-88.1)
	16,18,31,33,45,52,58	*MAL*	0.25	100 (84.6-100)	14.3 (3.1-36.3)
	16,18	*CADM1*	3.5	90.5 (69.6-98.8)	35.0 (15.4-59.2)
	16,18,31,33,45,52,58	*CADM1*	3.5	95.2 (76.2-99.9)	20.0 (5.7-43.7)
	16,18	*miR124-2/MAL*	3.7/0.25	95.2 (76.2-99.9)	55.0 (31.5-76.9)
	16,18,31,33,45,52,58	*miR124-2/MAL*	3.7/0.25	100 (83.9-100)	25.0 (8.7-49.0)
	16,18	*miR124-2/CADM1*	3.7/3.5	95.2 (76.2-99.9)	25.0 (8.7-49.1)
	16,18,31,33,45,52,58	*miR124-2/CADM1*	3.7/3.5	95.2 (76.2-99.9)	15.0 (3.2-37.9)
	16,18	*MAL/CADM1*	0.25/3.7	100 (85.9-100)	30.0 (11.9-54.3)
	16,18,31,33,45,52,58	*MAL/CADM1*	0.25/3.7	100 (83.9-100)	20.0 (5.7-43.7)
Vaginal, self-collected	16	NA	NA	52.2 (30.6-73.2)	81.0 (58.1-94.6)
	16,18	NA	NA	60.9 (38.5-80.3)	81.0 (58.1-94.6)
	16,18,31,33,45,52,58	NA	NA	95.7 (78.1-99.9)	33.3 (14.6-56.9)
	16,18	*miR124-2*	1.2	95.5 (77.2-99.9)	33.3 (14.6-50.7)
	16,18,31,33,45,52,58	*miR124-2*	1.2	100 (84.6-100)	14.3 (3.1-36.3)
	16,18	*MAL*	0.1	68.2 (45.1-86.1)	71.4 (47.8-88.7)
	16,18,31,33,45,52,58	*MAL*	0.1	95.5 (77.2-99.9)	28.6 (11.3-52.2)
	16,18	*CADM1*	3.9	90.9 (70.8-98.9)	33.3 (14.6-56.9)

For peer review only - http://bmjopen.bmj.com/site/about/guidelines.xhtml

For peer review only

16,18,31,33,45,52,58	*CADM1*	3.9	100 (84.6-100)	9.5 (1.2-30.4)
16,18	*miR124-2/MAL*	1.2/0.1	95.5 (77.2-99.9)	33.3 (14.6-57.0)
16,18,31,33,45,52,58	*miR124-2/MAL*	1.2/0.1	100 (84.6-100)	14.3 (3.1-36.3)
16,18	*miR124-2/CADM1*	1.2/3.9	100 (84.6-100)	23.8 (8.2-47.2)
16,18,31,33,45,52,58	*miR124-2/CADM1*	1.2/3.9	100 (84.6-100)	4.8 (0.1-23.8)
16,18	*MAL/CADM1*	0.1/3.9	90.9 (70.8-98.9)	28.6 (11.3-52.2)
16,18,31,33,45,52,58	*MAL/CADM1*	0.1/3.9	100 (84.6-100)	9.5 (1.2-30.4)

[a] Detection of at least one of the indicated HPV genotypes

[b] Positive for at least one of the indicated host genes

Shading rows show markers or combination of markers with the best diagnostic performance and those with the maximal sensitivity for detection of HSIL+

For peer review only

Comparison of levels of methylation between paired self-collected and clinician-collected samples

There was generally a lower percentage of methylation for each marker in vaginal samples compared with their paired cervical samples (Figure 3). However, this only reached statistical significance for *miR124-2* in HSIL (p=0.0002) (Figure 3).

Agreement of paired self-collected and clinician-collected samples for *miR124-2*, *MAL* and *CADM1* methylation

Analysis was performed for 40 patients with paired, assessable samples. Initial analysis was performed without considering the underlying disease status. OPA, PPA and NPA were 62.5%, 77.3% and 44.4% respectively for *miR124-2* methylation, 77.5%, 46.2% and 92.6% respectively for *MAL* methylation, and 57.5%, 67.9% and 33.3% respectively for *CADM1*.

In analyses limited to HSIL+ paired clinician- and self-collected samples, the OPA, PPA and NPA were 75.0% (53.1-88.8), 81.3% (57.0-93.4) and 50.0% (15.0-85.0) respectively for *miR124-2* methylation, 65.0% (43.3-81.9), 45.5% (21.3-72.0) and 88.9% (56.5-98.0) respectively for *MAL* methylation; and 65.0% (43.3-81.9), 68.8% (44.4-85.8) and 50.0% (15.0-85.0) respectively for *CADM1*.

For peer review only - http://bmjopen.bmj.com/site/about/guidelines.xhtml

For peer review only

Discussion

In this study, we explored *CADM1*, *MAL* and *miR124-2* DNA methylation using clinician- and self-collected HPV+ samples from Papua New Guinea, a setting with a high burden of cervical disease. In clinician-collected cervical samples, DNA methylation of *MAL* and *miR124-2* were significantly higher in HSIL/cancer compared with normal/LSIL samples, while the methylation of *CADM1* failed to distinguish between cytology grades. Methylation of *miR124-2* showed the highest AUC to distinguish HSIL from normal/LSIL and the second-highest performance to predict SCC, with AUC values above 0.819. Exploration of diagnostic performance of *miR124-2* in combination with *MAL* for detection of HSIL+, showed an excellent performance (sensitivity 90.5%, specificity 70%). Similar findings were obtained with self-collected samples, though their performance was reduced. Overall, *miR124-2* in combination with *MAL* DNA methylation performed well for the detection of HSIL+ in women from LMIC. Addition of HPV16/18 or extensive genotyping improved sensitivity at the expense of lower specificity and higher referrals to immediate treatment. *miR124-2/MAL* methylation markers are suitable for further evaluation against histology end points, and subsequently in LMIC-based field studies for triage of hrHPV+ women to identify high-grade disease.

Increased levels of *miR124-2*, *CADM1* and *MAL* methylation according to lesion grade have been observed in studies performed in clinician-collected cervical samples from high-income populations (27-29). *miR124-2* methylation alone or in combination with *CADM1* and *MAL* has high sensitivity and specificity for the detection of CIN3+ in HPV+ clinician-collected cervical specimens in women from high-income countries (sensitivity 68.0–94.7%, specificity 50.7–78.9%) (30).

For peer review only - http://bmjopen.bmj.com/site/about/guidelines.xhtml

For peer review only

Few studies have been performed in LMIC using clinician-collected samples. An analysis of the same markers in HPV+ and HIV+ women from Kenya found the AUC of *miR124-2* methylation for CIN2+ and CIN3+ were 0.730 and 0.810 respectively (31), marginally lower to those obtained in the current exploratory study. However, these results are not directly comparable to ours as their lesion grade was based on histological diagnosis and HIV+ participants. In the current study, we were unable to stratify by HIV status, but assume a low HIV prevalence as the HIV prevalence in the Papua New Guinean general population is <1%. Two other studies performed in South Africa and Thailand (upper middle-income countries) also showed good performance of some of these markers, alone or in combination, for the detection of HSIL+ (32, 33).

CADM1 was not a good diagnostic marker, consistent with the findings from two other studies performed in high-income countries (34, 35).

No research has been conducted looking methylation in self-collected samples from LMIC. Diagnostic performance of *miR124-2* for detection of HSIL+ in self-collected samples showed a sensitivity of 77.3% and specificity of 47.6%, while combining *miR124-2* with *MAL* increased the sensitivity to 81.8%, showing promise for self-sampling applications. While the sensitivity/specificity was slightly lower than clinician-collected samples, this may be offset by higher screening rates achieved by this more acceptable method of collection.

For peer review only - http://bmjopen.bmj.com/site/about/guidelines.xhtml

By comparison, studies of *miR124-2* and *MAL* single or in combination with other methylation markers for detection of CIN3+ in self-collected samples from high-income settings, have shown a wide range of sensitivities and specificities with values between 37.5–78.4% and 47.2–98% respectively (36-39). This wide variation may be explained by variable methodology [self-sampling collection devices, methylation detection assays, genes analysed, thresholds to define methylation positivity, study populations, disease end points (cytological HSIL or histological CIN2/3+), etc].

Triage combining HPV16/18 genotyping and methylation analysis has shown promise in clinician-collected samples in high-income settings (37, 38, 40, 41). Methylation analysis of *miR124-2/MAL* combined with HPV16/18 genotyping had a sensitivity for CIN3+ of 77.6% and specificity of 54.8% (40). A recent prospective cohort study in an upper middle-income country analysing methylation markers ZNF671/ASTN1/ITGA4/RXFP3/SOX17/DLX1 showed a sensitivity and specificity of 96.6% and 58.3% respectively to detect CIN2+ when combining methylation and HPV16/18 (42). In our study, combining HPV16/18 detection with *miR124-2/MAL* methylation improved sensitivity (increasing from 90.5 to 95.2%), but reduced specificity (decreasing from 70.0% to 55.0%). In self-collected samples the sensitivity was similar to clinician-collected samples (95.5%) but the specificity was lower (33.3%).

Combining extended genotyping (HPV16/18/31/33/45/52/58) with miR124-2/MAL methylation had similar sensitivity to *miR124/MAL/HPV16/18* testing, but in our setting showed lower specificity. Regardless of study population, HPV prevalence, sample type, marker panel and age, the combination of host cell DNA methylation analysis with partial or

For peer review only - http://bmjopen.bmj.com/site/about/guidelines.xhtml

extended genotyping could serve as a potential triage strategy for hrHPV+ women, at the expense of a higher referral to treatment.

Self-collected samples generally had lower levels of methylation than paired clinician-collected samples, likely due to lower diseased/normal cell ratios. For example, values for the GynTect® assay (detecting six methylation markers) were significantly lower for self-collected than clinician-collected samples and the overall concordance was moderate (kappa 0.394) (43). Further work to identify markers suitable for self-collection should consider using self-collected samples for this purpose (44).

Limitations of this study include the small sample size which led to broad confidence intervals; however, it aimed to provide estimated effects (point estimates with confidence intervals) for all measures of interest which are valuable for planning subsequent studies in LMIC. It was not feasible to conduct cervical biopsies in this setting due to limited specialist staff and infrastructure; hence LBC HSIL+ was the diagnostic reference standard rather than a histological end point and this may have impacted estimates of sensitivity/specificity for methylation markers. However, to avoid disease misclassification by cytology all slides were assessed by two independent experienced cytologists, and dual p16/Ki-67 immunostaining was performed to resolve disagreements. A triage test based on methylation markers could be highly suited to regions without access to colposcopy, where the combination of HPV detection, methylation analysis and cytology could confirm HSIL+ in the absence of colposcopy examination. Additional information is required to understand the long-term safety of methylation markers in screening programs, including longitudinal data from a low-

For peer review only

resource setting and analysis of methylation in self-collected samples from general population and HIV+ women.

Conclusions

DNA methylation of *miR124-2/MAL* alone or in combination with HPV16/18 is very promising as a triage test for the prediction of HSIL/SCC among women with oncogenic HPV infections. Self-collected samples showed reduced specificity compared to clinician-collected samples, indicating a need to identify additional biomarkers for this sample type. Future evaluation of these results against histological end points, and subsequently in different populations may lead to better tests to manage HPV+ women in LMIC.

Acknowledgements

We thank the women who participated in this research and their families and community, without whom this work would not have been possible. Special thanks to the Papua New Guinea study team who collaborated to the fieldwork, and Cepheid (Sunnyvale, CA, USA) for donating Xpert HPV cartridges.

Authors' Contributions

MM: Conceptualization, methodology, laboratory work, writing original draft, software analysis. DAM. Supervision, methodology, investigation, writing–review and editing. GT: Coordinated the evaluation of liquid-based cytology specimens, review and editing. SMG:

For peer review only - http://bmjopen.bmj.com/site/about/guidelines.xhtml

For peer review only

502 Conceived the original trial, supervision, funding acquisition, project administration, writing—
503 review and editing. SP: Software analysis, data curation, review and editing. PB, DH, PJT:
504 Investigation, laboratory work, review and editing. SGB: Conceived the original trial,
505 coordinated clinical implementation, review and editing. JB, JG, ZK, GMM: coordinated clinical
506 implementation in the original trial, review and editing. AC, GH investigation, review and
507 editing. JMB, MS, JMK: Conceived the original trial, funding acquisition, review and editing.
508 AJV: Conceived the original trial, conceptualization, funding acquisition, project
509 administration, writing, review and editing GM: Conceptualization, supervision, funding
510 acquisition, investigation, project administration, writing, review and editing.

511

512

For peer review only - http://bmjopen.bmj.com/site/about/guidelines.xhtml

References

1. Sung H, Ferlay J, Siegel RL, et al. Global cancer statistics 2020: GLOBOCAN estimates of incidence and mortality worldwide for 36 cancers in 185 countries. CA Cancer J Clin. 2021;71(3):209-249.

2. Lemp JM, De Neve JW, Bussmann H, et al. Lifetime prevalence of cervical cancer screening in 55 low- and middle-income countries. JAMA. 2020;324:1532-1542.

3. Ferlay J, Ervik M, Lam F, et al. Global Cancer Observatory: Cancer Today. Lyon, France: International Agency for Research on Cancer. Available from: https://gco.iarc.fr/today, accessed 20 07 2021.

4. PATH. Global HPV Vaccine Introduction Overview: projected and current national introductions, demonstration/pilot projects, gender-neutral vaccination programs, and global HPV vaccine introduction maps (2006-2023). PATH; 2020. Accessed November 23, 2020. Path.org/resources/global-hpv-vaccine-introduction-overview/ 179.

5. Simelela PN. WHO global strategy to eliminate cervical cancer as a public health problem: An opportunity to make it a disease of the past. Int J Gynaecol Obstet. 2021;152(1):1-3.

6. WHO. WHO guideline for screening and treatment of cervical pre-cancer lesions for cervical cancer prevention, second edition. Geneva: World Health Organization; 2021

7. Toliman P, Badman SG, Gabuzzi J, et al. Field Evaluation of Xpert HPV Point-of-Care Test for Detection of Human Papillomavirus Infection by Use of Self-Collected Vaginal and Clinician-Collected Cervical Specimens. J Clin Microbiol. 2016;54(7):1734-7.

8. Toliman PJ, Kaldor JM, Badman SG, et al. Evaluation of self-collected vaginal specimens for the detection of high-risk human papillomavirus infection and the prediction of high-grade

For peer review only - http://bmjopen.bmj.com/site/about/guidelines.xhtml

cervical intraepithelial lesions in a high-burden, low-resource setting. Clin Microbiol Infect. 2019;25:496-503.

9. Arbyn M, Smith SB, Temin S, et al. Detecting cervical precancer and reaching under screened women by using HPV testing on self samples: updated meta-analyses. BMJ (Clinical research ed) 2018; 363: k4823.

10. De Pauw H, Donders G, Weyers S, et al. Cervical cancer screening using HPV tests on self-samples: attitudes and preferences of women participating in the VALHUDES study. Arch Public Health. 2021;79(1):155.

11. Hawkes D, Keung MHT, Huang Y, et al. Self-Collection for Cervical Screening Programs: From Research to Reality. Cancers (Basel). 2020;12(4):1053.

12. Murillo R, Gamboa O, Hernández G, et al. Accuracy of combined molecular and morphology-based triage for HPV-positive women in routine cervical cancer screening services from Colombia. Prev Med. 2021;153:106801.

13. Mirabello L, Sun C, Ghosh A, et al. Methylation of human papillomavirus type 16 genome and risk of cervical precancer in a Costa Rican population. J. Natl Cancer Inst. 2012;104:556–565.

14. De Strooper LM, van Zummeren M, Steenbergen RD, . CADM1, MAL and miR124-2 methylation analysis in cervical scrapes to detect cervical and endometrial cancer. J Clin Pathol. 2014; 67(12): 1067-71.

15. Cook D A, Krajden M, Brentnall A R, et al. Evaluation of a validated methylation triage signature for human papillomavirus positive women in the HPV FOCAL cervical cancer screening trial. Int J Cancer. 2018; 144: 2587–2595.

For peer review only - http://bmjopen.bmj.com/site/about/guidelines.xhtml

16. Bonde J, Floore A, Ejegod D, et al. Methylation markers FAM19A4 and miR124-2 as triage strategy for primary human papillomavirus screen positive women: A large European multicenter study. Int J Cancer. 2021;148(2):396-405.

17. Verhoef VMJ, van Kemenade FJ, Rozendaal L, et al. Follow-up of high-risk HPV positive women by combined cytology and bi-marker CADM1/MAL methylation analysis on cervical scrapes. Gynecol Oncol. 2015;137(1):55–59.

18. Zhang L, Tan W, Yang H, et al. Detection of Host Cell Gene/HPV DNA Methylation Markers: A Promising Triage Approach for Cervical Cancer. Front Oncol. 2022;12:831949.

19. Vallely AJ, Saville M, Badman SG, et al. Point-of-care HPV-DNA testing of self-collected specimens and same-day thermal ablation for the early detection and treatment of cervical pre-cancer in high-burden, low-resource settings: Results of a prospective intervention trial in Papua New Guinea (HPV-STAT). Lancet Glob Health. 2022 Sep;10(9):e1336-e1346.

20. Li D, Xie Z, Pape ML, Dye T. An evaluation of statistical methods for DNA methylation microarray data analysis. BMC Bioinformatics. 2015 Jul 10;16:217.

21. Phillips S, Cassells K, Garland SM, et al. Gene methylation of CADM1 and MAL identified as a biomarker of high grade anal intraepithelial neoplasia. Sci Rep. 2022;12(1):3565.

22. Hesselink AT, Heideman DA, Steenbergen RD, et al. Methylation marker analysis of self-sampled cervico-vaginal lavage specimens to triage high-risk HPV-positive women for colposcopy. Int J Cancer. 2014;135(4):880-6.

23. Unal I. Defining an Optimal Cut-Point Value in ROC Analysis: An Alternative Approach. Comput Math Methods Med. 2017;2017:3762651.

24.Thiele C & Hirschfeld G. Cutpointr: Improved estimation and validation of optimal cutpoints in R. J. Stat. Softw. 2021;98(11): 1–27.

For peer review only - http://bmjopen.bmj.com/site/about/guidelines.xhtml

25. Food, U. S. “Drug Administration. Statistical Guidance on Reporting Results from Studies Evaluating Diagnostic Tests-Guidance for Industry and FDA Staff.” (2007).

26. Bossuyt PM, Reitsma JB, Bruns DE, et al. STARD 2015: An Updated List of Essential Items for Reporting Diagnostic Accuracy Studies.

27. Hesselink AT, Heideman DA, Steenbergen RD, et al. Combined promoter methylation analysis of CADM1 and MAL: an objective triage tool for high-risk human papillomavirus DNA-positive women. Clin Cancer Res. 2011;17:2459–2465.

28. Bierkens M, Hesselink AT, Meijer CJ, et al. CADM1 and MAL promoter methylation levels in hrHPV-positive cervical scrapes increase proportional to degree and duration of underlying cervical disease. Int J Cancer. 2013;133:1293–1299

29. Clarke MA, Luhn P, Gage JC, et al. Discovery and validation of candidate host DNA methylation markers for detection of cervical precancer and cancer. Int J Cancer. 2017;141(4):701-710

30. Kremer WW, Steenbergen R, Heideman D, et al. The use of host cell DNA methylation analysis in the detection and management of women with advanced cervical intraepithelial neoplasia: a review. BJOG. 2021;128(3):504-514.

31. De Vuyst H, Franceschi S, Plummer M, et al. Methylation levels of CADM1, MAL, and MIR124-2 in cervical scrapes for triage of HIV-infected, high-risk HPV-positive women in Kenya. J Acquir Immune Defic Syndr. 2015;70:311–8.

32. Van Zummeren M, Kremer WW, Van Aardt MC, et al. Selection of women at risk for cervical cancer in an HIV-infected South African population. AIDS. 2017;31:1945–53.

For peer review only

33. Dankai W, Khunamornpong S, Siriaunkgul S, et al. Role of genomic DNA methylation in detection of cytologic and histologic abnormalities in high risk HPV infected women. PloS One. 2019;14(1):e0210289

34. Vasiljevic N, Scibior-Bentkowska D, Brentnall AR, et al. Credentialing of DNA methylation assays for human genes as diagnostic biomarkers of cervical intraepithelial neoplasia in high-risk HPV positive women. Gynecol Oncol. 2014;132:709–14.

35. Salta S, Maia-Moço L, Estev~aoPereira H, et al. Performance of DNA methylation-based biomarkers in the cervical cancer screening program of northern Portugal: A feasibility study. Int J Cancer. 2021;149(11):1916-1925.

36. Verhoef VMJ, Bosgraaf RP, van Kemenade FJ, et al. Triage by methylation-marker testing versus cytology in women who test HPV-positive on self-collected cervicovaginal specimens (PROHTECT-3): a randomised controlled non-inferiority trial. Lancet Oncol. 2014;15(3):315–322.

37. De Strooper LMA, Verhoef VMJ, Berkhof J, et al. Validation of the FAM19A4/mir124-2 DNA methylation test for both lavage- and brush-based self-samples to detect cervical (pre)cancer in HPV positive. Gynecol Oncol. 2016;141:341–347.

38. Luttmer R, De Strooper LM, Dijkstra MG, et al. FAM19A4 methylation analysis in self samples compared with cervical scrapes for detecting cervical (pre)cancer in HPV-positive women. Br J Cancer. 2016;115:579–87

39. Del Mistro A, Frayle H, Rizzi M, et al. Methylation analysis and HPV genotyping of self-collected cervical samples from women not responding to screening invitation and review of the literature. PloS ONE. 2017;12(3):e0172226

For peer review only - http://bmjopen.bmj.com/site/about/guidelines.xhtml

40. Verhoef VMJ, Heideman DAM, van Kemenade FJ, et al. Methylation marker analysis and HPV16/18 genotyping in high-risk HPV positive self-sampled specimens to identify women with high grade CIN or cervical cancer. Gynecol Oncol. 2014;135(1):58–63.

41. Leeman A, Del Pino M, Marimon L, et al. Reliable Identification of Women With CIN3+ Using hrHPV Genotyping and Methylation Markers in a Cytology-Screened Referral Population. Int J Cancer. 2019; 144:160–8.

42. Zhang L, Zhao X, Hu S, et al. Triage performance and predictive value of the human gene methylation panel among women positive on self-collected HPV test: Results from a prospective cohort study. Int J Cancer. 2022;151(6):878-887

43. Klischke L, von Ehr J, Kohls F, et al. Performance of a six-methylation-marker assay on self-collected cervical samples – A feasibility study. J Virol Methods. 2021;295:114219.

44. Verlaat W, Snoek BC, Heideman DAM, et al. Identification and Validation of a 3-Gene Methylation Classifier for HPV-Based Cervical Screening on Self-Samples. Clin Cancer Res. 2018;24(14):3456-3464.

For peer review only - http://bmjopen.bmj.com/site/about/guidelines.xhtml

Figure 1. Percentage of DNA methylation of gene *CADM1, MAL* and *miR124-2* according to cytological grade in oncogenic HPV positive clinician-collected cervical samples (Cervical) in the top panel and self-collected vaginal samples (Vaginal) in the bottom panel. Whiskers correspond to the first and third quartiles (the 25th and 75th percentiles). Overall significance by Kruskal-Wallis is indicated.

For peer review only

For peer review only - http://bmjopen.bmj.com/site/about/guidelines.xhtml

Figure 2. Receiver operating characteristic (ROC) curve values of the performance of DNA methylation of *CADM1, MAL* and *miR124-2* for distinguishing HSIL from LSIL/normal, and SCC from LSIL/normal, stratified by type; clinician-collected cervical samples (black bars) or self-collected vaginal samples (grey bars).

For peer review only

Figure 3. Percentage methylation of gene *CADM1, MAL* and *miR124-2* comparing paired clinician-collected cervical samples (Cervical) and self-collected vaginal samples (Vaginal). Comparisons assessed by Wilcoxon signed rank test, with whiskers corresponding to the first and third quartiles (the 25th and 75th percentiles).

For peer review only

For peer review only - http://bmjopen.bmj.com/site/about/guidelines.xhtml

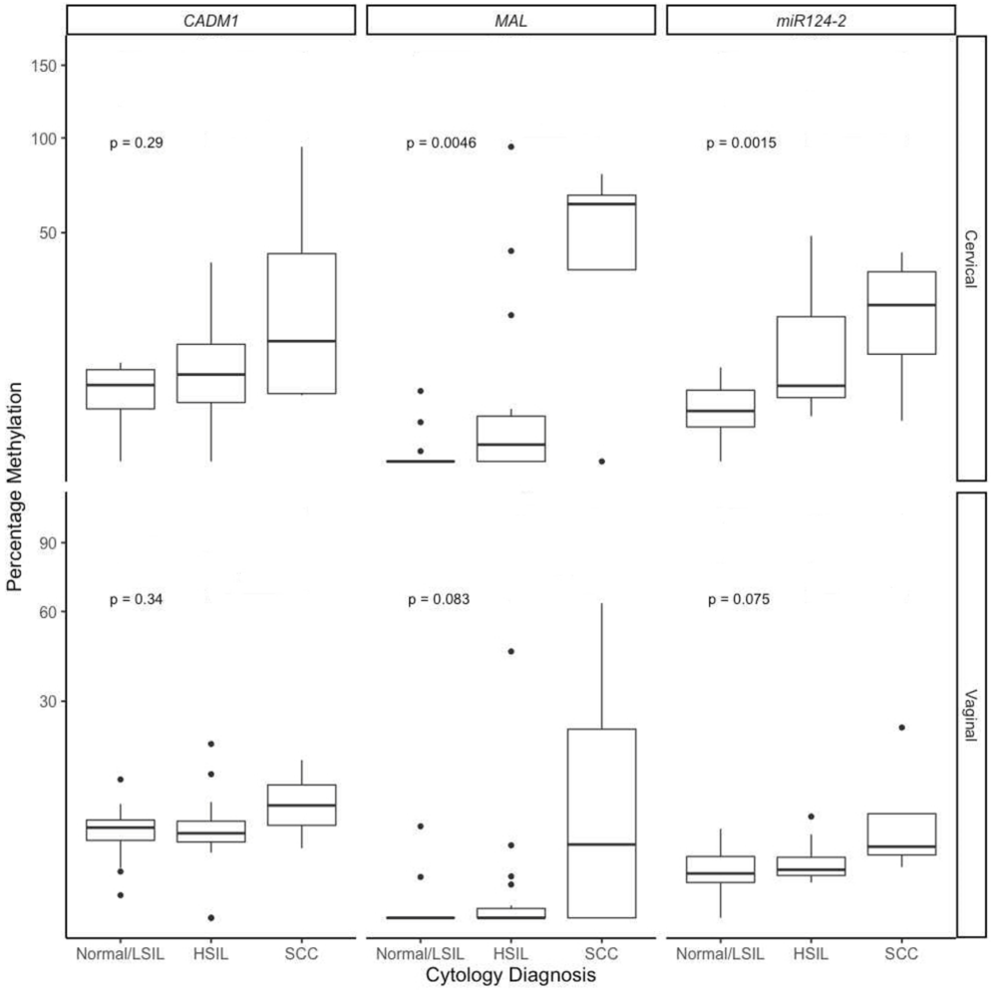

Figure 1. Percentage of DNA methylation of gene CADM1, MAL and miR124-2 according to cytological grade in oncogenic HPV positive clinician-collected cervical samples (Cervical) in the top panel and self-collected vaginal samples (Vaginal) in the bottom panel. Whiskers correspond to the first and third quartiles (the 25th and 75th percentiles). Overall significance by Kruskal-Wallis is indicated.

190x190mm (133 x 133 DPI)

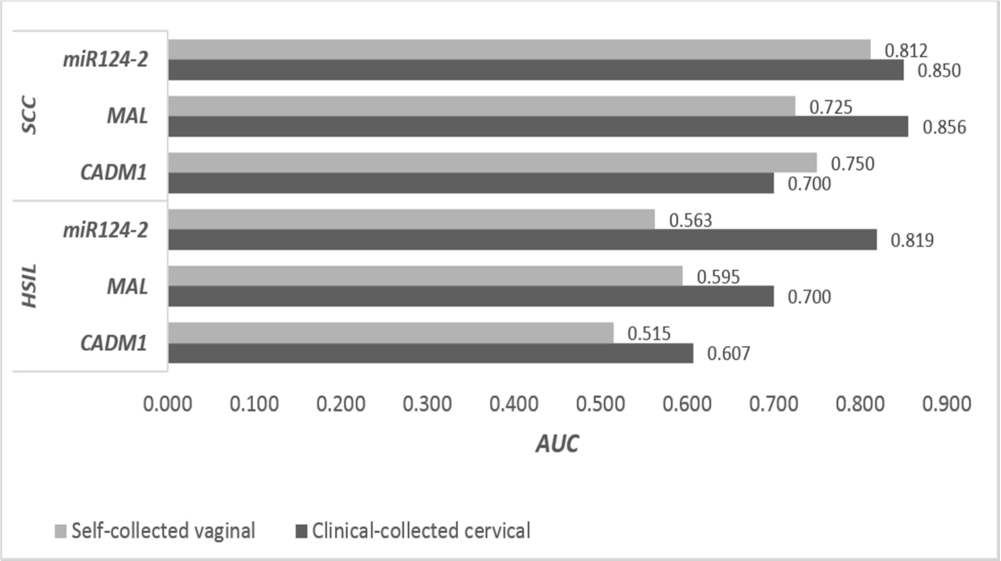

Figure 2. Receiver operating characteristic (ROC) curve values of the performance of DNA methylation of CADM1, MAL and miR124-2 for distinguishing HSIL from LSIL/normal, and SCC from LSIL/normal, stratified by type; clinician-collected cervical samples (black bars) or self-collected vaginal samples (grey bars).

295x142mm (117 x 137 DPI)

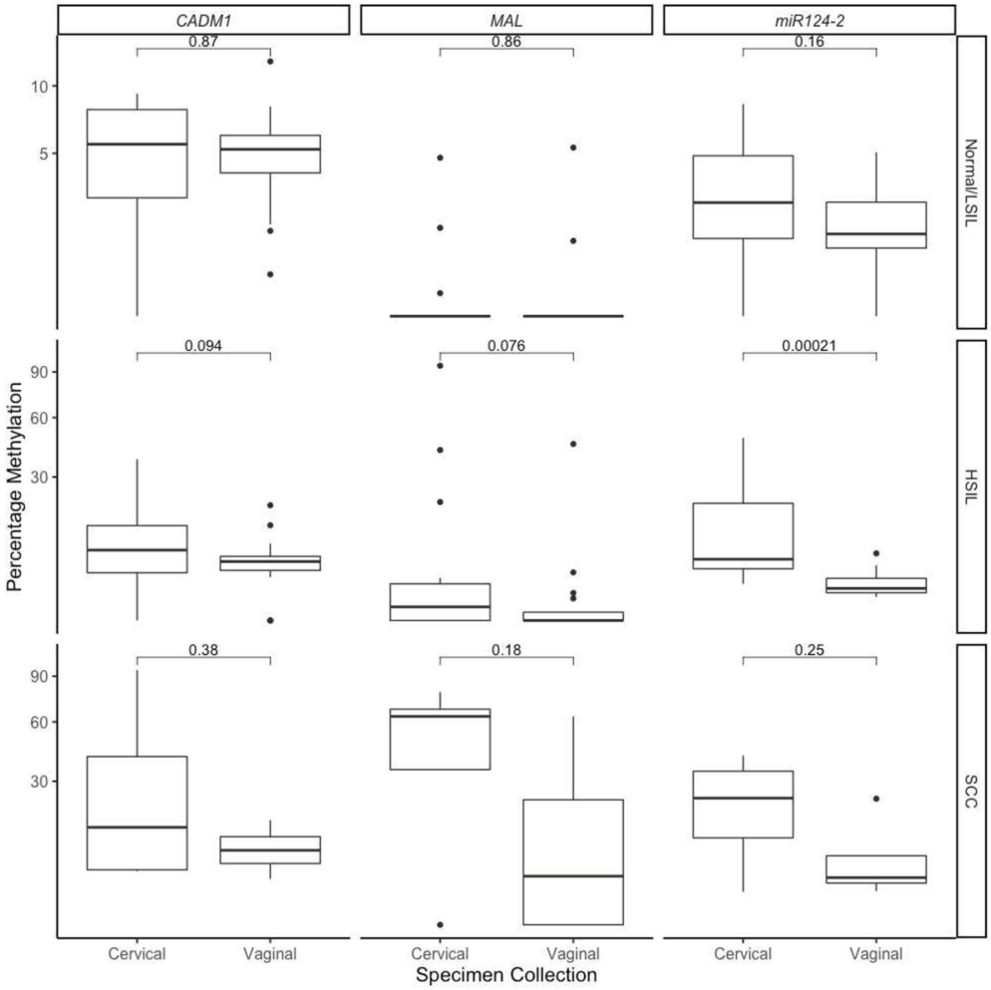

Figure 3. Percentage methylation of gene CADM1, MAL and miR124-2 comparing paired clinician-collected cervical samples (Cervical) and self-collected vaginal samples (Vaginal). Comparisons assessed by Wilcoxon signed rank test, with whiskers corresponding to the first and third quartiles (the 25th and 75th percentiles).

190x190mm (133 x 133 DPI)

Reporting checklist for diagnostic test accuracy study.

Based on the STARD guidelines.

Instructions to authors

Complete this checklist by entering the page numbers from your manuscript where readers will find each of the items listed below.

Your article may not currently address all the items on the checklist. Please modify your text to include the missing information. If you are certain that an item does not apply, please write "n/a" and provide a short explanation.

Upload your completed checklist as an extra file when you submit to a journal.

In your methods section, say that you used the STARDreporting guidelines, and cite them as:

Bossuyt PM, Reitsma JB, Bruns DE, Gatsonis CA, Glasziou PP, Irwig L, LijmerJG Moher D, Rennie D, de Vet HCW, Kressel HY, Rifai N, Golub RM, Altman DG, Hooft L, Korevaar DA, Cohen JF, For the STARD Group. STARD 2015: An Updated List of Essential Items for Reporting Diagnostic Accuracy Studies.

		Reporting Item	Page Number
**Title or abstract**			
None	#1	Identification as a study of diagnostic accuracy using at least one measure of accuracy (such	3

For peer review only

		as sensitivity, specificity, predictive values, or AUC)	
**Abstract**			
None	#2	Structured summary of study design, methods, results, and conclusions (for specific guidance, see STARD for Abstracts https://www.equator-network.org/reporting-guidelines/stard-abstracts/)	3
**Introduction**			
None	#3	Scientific and clinical background, including the intended use and clinical role of the index test	6-7
None	#4	Study objectives and hypotheses	8
**Methods**			
Study design	#5	Whether data collection was planned before the index test and reference standard were performed (prospective study) or after (retrospective study)	9-10
Participants	#6	Eligibility criteria	9-10

For peer review only

Participants	#7	On what basis potentially eligible participants were identified (such as symptoms, results from previous tests, inclusion in registry)	9-10
Participants	#8	Where and when potentially eligible participants were identified (setting, location and dates)	9,11
Participants	#9	Whether participants formed a consecutive, random or convenience series	9,11
Test methods	#10	Index and reference tests in sufficient detail to allow replication	9-13
Test methods	#11	Rationale for choosing the reference standard (if alternatives exist)	10
Test methods	#12	Definition of and rationale for test positivity cut-offs or result categories of the index and reference tests, distinguishing pre-specified from exploratory	10,12-13
Test methods	#13	Whether clinical information and reference standard results were available to the performers / readers of the index test; Whether clinical information and index test results were available to the assessors of the reference standard	11

For peer review only

Analysis	#14	Methods for estimating or comparing measures of diagnostic accuracy	12-13
Analysis	#15	How indeterminate index test or reference standard results were handled	12-13
Analysis	#16	How missing data on the index test and reference standard were handled	12
Analysis	#17	Any analyses of variability in diagnostic accuracy, distinguishing pre-specified from exploratory	10,13
Analysis	#18	Intended sample size and how it was determined	9,11
**Results**			
Participants	#19	Flow of participants, using a diagram	NA<br>This is an exploratory study. The flow of participants was outlined in detail in the results section, and thus a diagram was not deemed necessary.
Participants	#20	Baseline demographic and clinical characteristics of participants	15

For peer review only

Participants	#21	Distribution of severity of disease in those with the target condition, and distribution of alternative diagnoses in those without the target condition	15
Participants	#22	Time interval and any clinical interventions between index test and reference standard	11,15
Test results	#23	Cross tabulation of the index test results (or their distribution) by the results of the reference standard	15,16,22
Test results	#24	Estimates of diagnostic accuracy and their precision (such as 95% confidence intervals)	16-21
Test results	#25	Any adverse events from performing the index test or the reference standard	15
**Discussion**			
None	#26	Study limitations, including sources of potential bias, statistical uncertainty, and generalisability	26
None	#27	Implications for practice, including the intended use and clinical role of the index test	26-27
**Other information**			
None	#28	Registration number and name of registry	9

For peer review only

None	#29	Where the full study protocol can be accessed	9
None	#30	Sources of funding and other support; role of funders	2

The STARD checklist is distributed under the terms of the Creative Commons Attribution License CC-BY. This checklist was completed on 10. October 2023 using https://www.goodreports.org/, a tool made by the EQUATOR Network in collaboration with Penelope.ai

For peer review only

Performance of CADM1, MAL and miR124-2 methylation as triage markers for early detection of cervical cancer in self- and clinician-collected samples: an exploratory observational study in Papua New Guinea

Journal:	*BMJ Open*
Manuscript ID	bmjopen-2023-081282.R2
Article Type:	Original research
Date Submitted by the Author:	24-May-2024
Complete List of Authors:	Molano, Monica; The Royal Women's Hospital, Centre for Women’s Infectious Diseases; Murdoch Children's Research Institute<br>Machalek, Dorothy; University of New South Wales, Kirby Institute; The Royal Women's Hospital, Centre for Women's Infectious Diseases<br>Tan, Grace; Australian Centre for the Prevention of Cervical Cancer<br>Garland, Suzanne; The Royal Women's Hospital; The University of Melbourne, Department of Obstetrics, Gynaecology and Newborn Health<br>Balgovind, Prisha; The Royal Women's Hospital; Murdoch Children's Research Institute<br>Haqshenas, Gholamreza; The Royal Women's Hospital; The University of Melbourne, Department of Obstetrics, Gynaecology and Newborn Health<br>Munnull, Gloria; Papua New Guinea Institute of Medical Research<br>Phillips, Samuel; The Royal Women's Hospital; Murdoch Children's Research Institute<br>Badman, Steven; University of New South Wales, Kirby Insitute<br>Bolnga, John ; Modilon General Hospital, Obstetrics and Gynaecology ; Papua New Guinea Institute of Medical Research, Sexual and Reproductive Health<br>Cornall, Alyssa; The Royal Women's Hospital; Murdoch Children's Research Institute<br>Gabuzzi, Josephine; Papua New Guinea Institute of Medical Research<br>Kombati, Zure; Mount Hagen General Hospital, Tininga Clinic<br>Brotherton, Julia; Australian Centre for the Prevention of Cervical Cancer; The University of Melbourne, School of Population Global Health<br>Saville, Marion; Australian Centre for the Prevention of Cervical Cancer<br>Hawkes, David; Australian Centre for the Prevention of Cervical Cancer<br>Kaldor, John; University of New South Wales, The Kirby Institute<br>Toliman, Pamela; University of New South Wales, Kirby Institute -Faculty of Medicine<br>Vallely, Andrew; Papua New Guinea Institute of Medical Research, Sexual and Reproductive Health Unit; University of New South Wales, The Kirby Institute<br>Murray , Gerald L ; The University of Melbourne - Parkville Campus, Obstetrics, Gynaecology and Newborn Health; The Royal Women's Hospital, Centre for Women’s Infectious Diseases
**Primary Subject Heading**:	Obstetrics and gynaecology

Secondary Subject Heading:	Oncology, Infectious diseases, Diagnostics
Keywords:	Molecular diagnostics < INFECTIOUS DISEASES, MOLECULAR BIOLOGY, Oncogenes < ONCOLOGY, Human Papillomavirus Viruses

SCHOLARONE™ Manuscripts

I, the Submitting Author has the right to grant and does grant on behalf of all authors of the Work (as defined in the below author licence), an exclusive licence and/or a non-exclusive licence for contributions from authors who are: i) UK Crown employees; ii) where BMJ has agreed a CC-BY licence shall apply, and/or iii) in accordance with the terms applicable for US Federal Government officers or employees acting as part of their official duties; on a worldwide, perpetual, irrevocable, royalty-free basis to BMJ Publishing Group Ltd ("BMJ") its licensees and where the relevant Journal is co-owned by BMJ to the co-owners of the Journal, to publish the Work in this journal and any other BMJ products and to exploit all rights, as set out in our licence.

The Submitting Author accepts and understands that any supply made under these terms is made by BMJ to the Submitting Author unless you are acting as an employee on behalf of your employer or a postgraduate student of an affiliated institution which is paying any applicable article publishing charge ("APC") for Open Access articles. Where the Submitting Author wishes to make the Work available on an Open Access basis (and intends to pay the relevant APC), the terms of reuse of such Open Access shall be governed by a Creative Commons licence – details of these licences and which Creative Commons licence will apply to this Work are set out in our licence referred to above.

Other than as permitted in any relevant BMJ Author's Self Archiving Policies, I confirm this Work has not been accepted for publication elsewhere, is not being considered for publication elsewhere and does not duplicate material already published. I confirm all authors consent to publication of this Work and authorise the granting of this licence.

For peer review only

Performance of *CADM1, MAL* and *miR124-2* methylation as triage markers for early detection of cervical cancer in self- and clinician-collected samples: an exploratory observational study in Papua New Guinea

Monica Molano[1,2], Dorothy A Machalek[1,3], Grace Tan[5], Suzanne M Garland[1,2,4], Samuel Phillips[1,2], Prisha Balgovind[1,2], Steven G Badman[3], Gholamreza Haqshenas[1,2,4], John Bolnga[6], Alyssa Cornall[1,2], Josephine Gabuzzi[6], Zure Kombati[7], Gloria Munnull[8], Julia M Brotherton[5], Marion Saville[5], David Hawkes[5], John M Kaldor[3], Pamela J Toliman[3], Andrew J Vallely[3]*, Gerald L. Murray[1,2,4]*

1. Centre for Women's Infectious Diseases, The Royal Women's Hospital, Melbourne, Victoria, Australia

2. Murdoch Children's Research Institute, Melbourne, Vic, Australia

3. The Kirby Institute, University of New South Wales, Sydney, NSW, Australia

4. Department of Obstetrics and Gynaecology, University of Melbourne, Parkville, Victoria, Australia

5. Australian Centre for the Prevention of Cervical Cancer, Melbourne, Victoria, Australia

6. Papua New Guinea Institute of Medical Research, Goroka, Papua New Guinea ; Department of Obstetrics and Gynaecology, Modilon General Hospital, Madang, Papua New Guinea.

7. Department of Pathology, Mt Hagen Provincial Hospital, WHP 281, Papua New Guinea

8. Papua New Guinea Institute of Medical Research, Goroka, Papua New Guinea; Department of Obstetrics and Gynaecology, Mt Hagen Provincial Hospital, Mount Hagen, Papua New Guinea.

*Indicates co-senior authors

Running title: DNA methylation-based triage markers for cervical cancer

Corresponding author:

For peer review only

For peer review only - http://bmjopen.bmj.com/site/about/guidelines.xhtml

Monica Molano, PhD

Telephone +61 3 8345 3691

Fax +61 3 9347 8235

Email Monica.MolanoLuque@thewomens.org.au

Disclosure of Potential Conflicts of Interest:

AJV, JG, JB, MG, PJT, SGB, JMK have received subsidised test kits for research from Cepheid. MS, JMB, GT, DH have received donated test kits for research from Abbott, BD, Cepheid, Hologic, Qiagen, Roche, and Seegene. AJV and MS jointly lead the *Elimination of Cervical Cancer in the Western Pacific (ECCWP)* program with philanthropic funding support from the Minderoo Foundation and the Frazer Family Foundation; and equipment, tests and consumables donated by Cepheid for HPV-based cervical screening in Papua New Guinea and Vanuatu. SMG is a member of the Global Advisory Board for HPV vaccines Merck and has led investigator-initiated grants from Merck on HPV in young women. MM, DAM, SP, PB, GH, ZK, GLM, declare no conflicting interests.

Grant Support: This work was funded through the National Health and Medical Research Council, Australia, (NHMRC) grants 1013209 and 1104938 (A.J.V), Government of Papua New Guinea (ICRAS 297/1), the NHMRC Centre for Research Excellence in Cervical Cancer Control APP1135172 (JMB, MS, AV, SMG, JMK, DAM, DH) and NHMRC Investigator Grant APP1197951 (S.M.G).

For peer review only - http://bmjopen.bmj.com/site/about/guidelines.xhtml

Abstract

Objective: WHO recommends human papillomavirus (HPV) testing for cervical screening, with triage of high-risk human papillomavirus (hrHPV) positive women. However, there are limitations to effective triage for low resource, high burden settings, such as Papua New Guinea. In this exploratory study we assessed the performance of host methylation as triage tools for predicting high-grade squamous intraepithelial lesions (HSIL) in self- and clinician-collected samples.

Design: Exploratory observational study

Setting: Provincial hospital, same day cervical screen-and-treat trial, Papua New Guinea

Participants: 44 hrHPV+ women, with paired self/clinician-collected samples [4 squamous cell carcinomas (SCC), 19 HSIL, 4 low-grade squamous intraepithelial lesions (LSIL), 17 normal]

Primary and secondary outcome measures: Methylation levels of *CADM1, MAL* and *miR124-2* analysed by methylation-specific PCRs against the clinical endpoint of HSIL or SCC (HSIL+) measured using liquid-based-cytology (LBC)/p16-Ki67 stain.

Results: In clinician-collected samples, *MAL* and *miR124-2* methylation levels were significantly higher with increasing grade of disease (p=0.0046 and p<0.0015 respectively). *miR124-2* was the best predictor of HSIL (AUC 0.819), whilst *MAL* of SCC (AUC 0.856). In self-collected samples, *MAL* best predicted HSIL (AUC 0.595), whilst *miR124-2* SCC (AUC 0.812). Combined *miR124-2/MAL* methylation yielded sensitivity and specificity for HSIL+ of 90.5% (95%CI: 69.6-98.8) and 70% (45.7-88.1) respectively in clinician-collected samples, and 81.8% (59.7-94.8) and 47.6% (25.7-70.2) respectively in self-collected samples. *miR124-2/MAL* plus HPV16/HPV18 improved sensitivity for HSIL+ (95.2%, 76.2-99.9), but decreased specificity (55.0%, 31.5-76.9).

For peer review only - http://bmjopen.bmj.com/site/about/guidelines.xhtml

For peer review only

Conclusion: *miR124-2/MAL* methylation is a potential triage strategy for the detection of HSIL/SCC in LMIC.

Key words:

DNA Methylation, Cervical Cancer, Human papillomavirus, Diagnostic test, Epigenetics, Molecular diagnostics, Molecular Biology, Oncogenes.

Data availability statement: Limited data may be available on reasonable request from the corresponding author, subject to ethical approval.

Strengths and Limitations of this study

- The assessment of methylation markers in self-collected vaginal samples recollected in a low-middle income country allows the detection of HSIL+ and open the way to full molecular screening in these settings.
- A direct comparison of self-collected samples and paired clinician-collected samples was possible, thus identifying different performance and optimal cut-off values by sample type.
- Study design allowed exploration of the clinical performance of extended genotyping (HPV16/18/31/33/45/52/58) combined with host methylation analysis for the detection of HSIL.
- A small sample size led to broad confidence intervals.
- Due to limited specialist staff and infrastructure, liquid-based cytology was the

For peer review only - http://bmjopen.bmj.com/site/about/guidelines.xhtml

diagnostic reference standard rather than a histological end point.

Introduction

Cervical cancer is the fourth most frequently diagnosed cancer and the fourth leading cause of cancer-related death in women, with an estimated 604,000 new cases and 342,000 deaths worldwide in 2020 (1). Age standardised rates are disproportionally higher in low- and middle-income countries (LMIC) than in high-income countries (18.8 vs 11.3 per 100,000 for incidence: 12.4 vs 5.2 per 100,000 for mortality), primarily due to disparities in access to effective prevention, screening and treatment strategies (2-5). Infection with high-risk HPV types (hrHPV) may cause cervical abnormalities graded histologically as cervical intraepithelial neoplasia (CIN, grades 1-3) or cancer (principally SCC or adenocarcinoma), or graded cytologically (using exfoliated cells from the cervix) as LSIL, HSIL or SCC (6).

To achieve cervical cancer elimination (5), many countries are transitioning their cervical screening programs to HPV nucleic acid-based testing, which is more sensitive than cytology for detecting underlying high-grade disease. Furthermore, sample collection can be self-administered, which has been shown to increase screening participation in many settings (6-11). Another important advantage is that nucleic acid amplification testing and subsequent treatment can be performed at point of care, greatly simplifying program delivery, particularly

For peer review only - http://bmjopen.bmj.com/site/about/guidelines.xhtml

in low resource settings. These advances led the WHO, in 2021, to endorse HPV screen-and-treat guidelines for women in LMIC (6).

An important limitation of HPV testing is that it suffers from a lower specificity than cytology, leading to concern that the treatment of all hrHPV+ women would result in substantial overtreatment (12). Hence, a second (triage) test is recommended to identify hrHPV+ women at highest risk of having underlying disease requiring treatment. The triage technologies most used are cytology, partial genotyping for HPV16 or 18, and in many low resource settings, visual inspection using acetic acid or Lugol's iodine (12). However, these triage approaches present challenges for sensitivity and/or specificity in LMIC settings (8, 12).

Molecular triage tests are advantageous over current methods as they are less subjective and could be performed on multiple specimen types, including self-samples. In particular, aberrant host gene DNA methylation patterns in promoter regions of some genes has shown promise as a triage marker in cervical screening in high income countries. Aberrant methylation is pivotal in cervical carcinogenesis; it causes changes in gene expression, faulty condensation, and chromosomal instability (13,14). Increased DNA methylation has been associated with hrHPV persistence (13) and correlates with increasing severity of cervical disease (14, 15). DNA methylation markers such as *CADM1, MAL* and *miR124-2* have shown good performance for the detection of CIN2 or worse (CIN2+) among hrHPV+ women (14, 16, 17). However, data on their performance has been based on studies conducted in high income settings and among highly screened populations (18). There is currently no data on the performance of these markers among LMIC populations by using self-collected vaginal

For peer review only

samples, nor in settings where HPV-based screening-and- same day-treatment strategies are used.

Papua New Guinea has a high burden of cervical cancer, with age standardized incidence and mortality rates of 29.2 and 19.1 per 100,000, respectively, making it the second most common cancer among Papua New Guinean women (3). A trial study (HPV-STAT) performed in women from this country evaluated the clinical performance, treatment completion rates, adverse events profile, and acceptability of a fully integrated screening strategy, comprising point-of-care HPV test of self-collected specimens and same-day thermal ablation (19). This trial supported the introduction of HPV screening and same-day treatment for the control of cervical cancer in LMIC (19). In the current study, we explored the performance of *CADM1, MAL* and *miR124-2* DNA methylation markers alone and in combination with HPV16/18 and extended genotyping (HPV16/18/31/33/45/52/58) as a triage strategy for the detection of HSIL+ among HPV+ women that participated in this cervical screening trial. A direct comparison was also performed for each methylation marker, between paired self- and clinician-collected samples.

Materials and Methods

Study population and design of the trial

The exploratory study was conducted as a sub-study of HPV-STAT ('HPV self-collect, test and-same-day-treat') a prospective, single-arm intervention trial among women attending cervical screening services at two clinical sites in Papua New Guinea. The trial is registered with ISRCTN, ISRCTN13476702 (https://www.isrctn.com/editorial/retrieveFile/3ed9173e-cce5-

For peer review only - http://bmjopen.bmj.com/site/about/guidelines.xhtml

4158-a586-577775f0cbdd/35731). Study design, recruitment and protocols have been described (19). Briefly, between June 5, 2018, and Jan 6, 2020, 4285 women aged 30 to 59 years attending clinics for routine cervical screening at Modilon Hospital (Madang Province) and Mt Hagen General Hospital (Western Highlands Province), who meet the inclusion criteria were invited to participate. Women who were pregnant or who had given birth in the past 6 weeks, or women who had a history of cervical cancer or hysterectomy, were excluded (19). Those women able to provide written informed consent were eligible to participate, enrolled sequentially and collected a vaginal specimen using a cytobrush ("Just for Me", Preventative Oncology International, Cleveland Heights, Ohio), which was placed into a 20 ml ThinPrep vial (Hologic, Marlborough, MA) (19).

Point Of Care HPV testing and follow-up assessment

For self-collected vaginal specimens, a 1 ml aliquot was tested for the presence of oncogenic HPV genotypes using the Xpert HPV Test (GeneXpert; Cepheid, Sunnyvale, CA, USA) as per the manufacturer's instructions. Results were reported as 'HPV16', 'HPV18/45', and 'Other HPV' (a summary result for HPV31, 33, 35, 39, 51, 52, 56, 58, 59, 66 and 68) and were provided to women before midday to allow sufficient time for same-day counselling, pelvic examination and treatment/referral as indicated (19). All women with a negative HPV test were informed of their results and advised to return to the clinic for HPV-based screening in five years.

hrHPV+ women provided a cervical specimen collected by a clinician using a Cervex-Brush Combi (Rovers Medical Devices, Oss, The Netherlands), placed in a 20 ml ThinPrep vial, and stored at 4°C prior to shipment to the Australian Centre for the Prevention of Cervical Cancer in Melbourne, Australia for liquid-based cytology (LBC) and p16/Ki67 dual stain cytology. A

For peer review only - http://bmjopen.bmj.com/site/about/guidelines.xhtml

15% random sample of HPV negative women were also asked to provide a clinician-collected cervical specimen for LBC, as above.

Cytological assessment

LBC was carried out in accordance with established laboratory procedures at the Australian Centre for the Prevention of Cervical Cancer. Slides were independently assessed by two cytologists blinded to HPV test results. Where both cytologists agreed on a diagnosis of HSIL or HSIL+, a final diagnosis was recorded. If the assessment differed, dual p16/Ki-67 dual stain was carried out by using CINTec PLUS Cytology (Roche Diagnostics, Pleasanton, USA) to make a final diagnosis (19). The primary clinical endpoint was then HSIL or SCC (HSIL+) using LBC/p16-Ki67 stain. LBC was accepted as a reference standard rather than histology, which is the gold reference in high-resource settings. It was not feasible from a staffing and logistical perspective to provide colposcopy or to collect cervical biopsies for histological examination in Papua New Guinea.

Participants in the exploratory study and design

The sub-study included 44 paired cervical and vaginal samples from women participating in the trial at Mt Hagen General Hospital (Mount Hagen, Western Highlands Province). This included all 23 hrHPV+ HSIL+ (19 HSIL and 4 SCC) cases identified on LBC by the end of 2018 and 21 randomly selected hrHPV+ normal/LSIL samples (17 normal LBC and 4 LSIL). Sample size selection was driven by a published study by Li et al., 2015 that showed through simulation studies and real data from 2 studies downloaded from the NCBI Gene Expression Omnibus that at least 12 specimens in each group is needed to detect truly differential DNA methylation with enough power (≥ 80%), reproducible data and consistence when using

For peer review only - http://bmjopen.bmj.com/site/about/guidelines.xhtml

different statistical methods (20). Molecular biologists and technicians were blinded to point of care HPV and clinical diagnosis.

DNA Extraction and HPV specific typing

DNA was extracted as described previously, quantitated by Qubit® Fluorometer (Life technologies, California, USA), and assessed for integrity by quantitative PCR amplification of a 260 base-pair product of the human beta-globin gene (21). In addition, DNA extracted from cervical cell line SiHa (1-2 copies of HPV16 per cell, ATCC Cat# HTB-35, RRID:CVCL_0032; American Type Culture Collection (ATCC), Manassas, Virginia, USA; 100 ng) was used as positive control for methylation analysis. This DNA was confirmed to have only HPV16 by HPV typing for 28 HPV types (Anyplex II HPV28 Detection, Seegene, Seoul, South Korea).

HPV genotyping was performed using the Anyplex II HR HPV Detection multiplex assay (Seegene, Seoul, South Korea), which detects 14 oncogenic HPV genotypes (16, 18, 31, 33, 35, 39, 45, 51, 52, 56, 58, 59, 66, 68) and an internal control, according to manufacturer's recommendations.

Bisulphite DNA modification and Quantitative methylation-specific PCR for CADM1, MAL and miR124 genes

DNA from the paired samples (1-100ng) and control (SiHa, 100 ng) were bisulphite treated using Methylamp DNA modification Kit (Epigentek, Brooklyn, NY, USA) as per the manufacturer's instructions. Modified DNA was eluted in 40µL of the Methylamp elution

For peer review only

For peer review only - http://bmjopen.bmj.com/site/about/guidelines.xhtml

buffer. Samples without the adequate concentration were excluded from the bisulphite modification and methylation analysis.

Quantitative methylation-specific PCR (qMSP) targeting CpG sites in promoter regions of *CADM1* (promoter region M9), *MAL* (promoter region M1) and *miR124-2* (promoter region 2) were performed as previously described (14), with minor modifications. Due to the use of a different platform (Light Cycler 480 II, Roche), validations of the qPCRs were performed utilising duplicated dilution series of SiHa cell line (intra and inter assays of reproducibility) and by using a training panel of samples as reported previously (21). As a reference, a qMSP for the housekeeping gene b-actin *(ACTB*) was performed. Samples with Ct values for *ACTB* of >34 were considered *ACTB* negative as this indicated poor sample quality due to insufficient DNA or inadequate bisulphite conversion. Target DNA methylation levels were normalized to the reference gene as described previously (21).

Statistical Analysis

The percentage of methylation was calculated as described previously (22). Differences in the median percentage methylation by disease grade (SCC, HSIL, LSIL/normal) were visualised using box and whisker plots and compared using Wilcoxon test (between groups) and Kruskal-Wallis (overall analysis) (20, 21). Area under the receiver operating characteristic curve (AUC) was used to assess the ability of the methylated genes to classify HSIL or SCC, providing two main outcomes: the diagnostic accuracy of the test and the optimal cut off point value for dichotomization of the methylation test (23). The optimal cut off-point was calculated by using the maximum sum of sensitivity and specificity (24). Sensitivity [number of correct positives (i.e. positive for at least one marker)/ number of reference assay positives] and

For peer review only - http://bmjopen.bmj.com/site/about/guidelines.xhtml

specificity (number of correct negatives/number of reference assay negatives) were determined for the following triage strategies: (I) single gene methylation analysis, (II) combination of methylation markers, (III) HPV 16 genotyping, (IV) combination of HPV16/18 genotyping, (V) combination of HPV 16/18/31/33/45/52/58 genotyping, (VI) HPV16/18 with single and combined methylation analysis, and (VII) HPV 16/18/31/33/45/52/58 with single and combined methylation analysis.

Median percentage methylation for each marker was compared for self- and clinician-collected vaginal paired specimens using the Wilcoxon signed rank test. The vaginal specimen was considered the test while the cervical specimen acted as the non-reference standard. Agreements between DNA methylation results from cervical and vaginal specimens were determined using the optimal cut off point established for each gene by calculating positive percent agreement (PPA), negative percent agreement (NPA) and overall percent agreement (OPA) as per recommendations by the Food and Drug Administration, USA (25).

The results were analysed by using XLSTAT and the statistical platform R studio (v4.0.1) and programs ggplots2 (v3.3.2), ggpubr (v0.4), pROC (v1.16.2) and cutpointr (v1.1.1) (21, 24).

We used the STARD reporting guidelines for reporting diagnostic accuracy studies (26).

Ethical considerations

Approval of the trial was provided by the Medical Research Advisory Committee (MRAC) of the Papua New Guinea National Department of Health (approval number 17.36), the Institutional Review Board of the Papua New Guinea Institute of Medical Research (IRB 1712),

For peer review only - http://bmjopen.bmj.com/site/about/guidelines.xhtml

and the Human Research Ethics Committee (HREC) of UNSW Australia (approval number HC17631). Written informed consent was obtained from all participants prior to enrolment.

Patient and Public Involvement

There was no patient or public involvement in study design or conduct.

For peer review only

For peer review only - http://bmjopen.bmj.com/site/about/guidelines.xhtml

Results

DNA Methylation of CADM1, MAL and miR124-2 and associations with lesion grade

Overall, 44 hrHPV+ paired cervical and vaginal samples from women participating in the trial at Mt Hagen General Hospital were used [23 cases (19 HSIL and 4 SCC 8) and 21 normal/LSIL (17 normal LBC and 4 LSIL)]. Of these samples, 41 (93.2%) clinician-collected (4 SCC, 17 HSIL, 4 LSIL and 16 normal LBC) and 43 (97.7%) self-collected samples (4 SCC, 18 HSIL, 4 LSIL and 17 normal LBC) were deemed assessable. The methylation analysis was not used for the clinical management of the women, and thus did not induce any adverse effects.

In clinician-collected samples DNA methylation of *MAL* was higher with increasing disease grade from normal/LSIL samples (1.4%; 0.8-3.1), HSIL (median 2.0%; 95%CI:0.7-20.4) and SCC (63.3%; 46.8-67.9) (p=0.0046) (Figure 1, top central panel). Methylation of *mirR124-2* was also higher with increasing disease grade from normal/LSIL samples (2.6%; 1.1-5.1), HSIL (5.6%; 3.9-20.5) and SCC (24.1%; 12.4-34.5) (p=0.0015) (Figure 1, top right panel). Methylation of *CADM1* did not differ significantly with increasing cytology grade (Figure 1, top left panel).

Using clinician-collected samples, ROC analysis showed that *miR124-2* was the best methylation marker to distinguish SCC and HSIL (from normal/LSIL), with an AUC of 0.850 and 0.819, respectively (Figure 2 A, C). Methylation of *MAL* showed higher AUC for detecting SCC (0.856) than HSIL (0.700). *CADM1* methylation showed poor performance for detecting SCC or HSIL with AUC of 0.700 and 0.607 respectively (Figure 2 A, C).

For peer review only - http://bmjopen.bmj.com/site/about/guidelines.xhtml

In self-collected samples, there was a trend of increasing DNA methylation of *MAL* with disease grade for MAL and *miR124-2*, though this did not reach significance (p=0.083, p=0.075, respectively) (Figure 1, lower centre panel). As found for clinician-collected samples, methylation of *CADM1* did not differ significantly with increasing cytology grade (Figure 1, lower left panel).

ROC analysis showed that from self-collected samples, *miR124-2* was the best methylation marker to distinguish SCC, but not HSIL, from normal/LSIL with an AUC of 0.812 and 0.563, respectively (Figure 2 B, D). Methylation of *MAL* or *CADM1* showed a lower clinical performance for SCC (AUC of 0.725 and 0.750, respectively) than *miR124-2*, and a similar performance for HSIL (AUC of 0.595 and 0.515, respectively) (Figure 2 B, D).

Diagnostic performance of DNA methylation markers for the detection of HSIL or SCC

Diagnostic performance of individual DNA methylation markers for detecting HSIL+ in clinician-collected samples showed that *miR124-2* methylation had the highest sensitivity of 81.0% (95%CI: 58.1-94.6) and *MAL* methylation the highest specificity of 90.0% (68.3-98.8) (Table 1). A combination of *miR124-2* and *MAL* showed the optimal diagnostic performance with a sensitivity of 90.5% (69.6-98.8) and specificity of 70.0% (45.7-88.1). A combination of *miR124-2/MAL* and *CADM1* showed a maximal sensitivity of 100% (95% CI 83.9-100) at the expense of a low specificity, 25% (8.7-49.1).

In self-collected samples, *miR124-2* methylation had a sensitivity of 77.3 % (95% CI 54.6-92.2) for detecting HSIL+, but much lower specificity 47.6% (25.7-70.2). *MAL* showed the best

For peer review only - http://bmjopen.bmj.com/site/about/guidelines.xhtml

specificity 90.5% (69.6-98.8) at the expense of a low sensitivity 32.0% (13.9-54.9) (Table 1). A combination of *miR124-2* and *MAL* improved the diagnostic performance with a sensitivity of 81.8% (95% CI 59.7-94.8) and specificity of 47.6% (25.7-70.2). A combination of *miR124-2/MAL* and *CADM1* increased the sensitivity to 90.9% (70.8-98.9) at the expense of a low specificity of 23.8% (8.2-47.2) (Table 1).

For peer review only

Table 1. Performance of host DNA methylation markers for detection of HSIL+

Sample type	Gene[a]	Percent methylation cut-off	Sensitivity % (95% CI)	Specificity % (95% CI)
Cervical, clinician-collected	*miR124-2*	3.7	81.0 (58.1-94.6)	70.0 (45.7-88.1)
	MAL	0.25	57.1 (34.0-78.2)	90.0 (68.3-98.8)
	CADM1	3.5	80.9 (58.1-94.6)	40.0 (19.1-63.9)
	miR124-2/MAL	3.7/0.25	90.5 (69.6-98.8)	70.0 (45.7-88.1)
	miR124-2/CADM1	3.7/3.5	95.2 (76.2-99.9)	25.0 (8.7-49.1)
	MAL/CADM1	0.25/3.5	95.2 (76.2-99.9)	35.0 (15.4-59.2)
	miR124-2/MAL/CADM1	3.7/0.25/3.5	100 (83.9-100)	25.0 (8.7-49.1)
Vaginal, self-collected	*miR124-2*	1.2	77.3 (54.6-92.2)	47.6 (25.7-70.2)
	MAL	0.1	32.0 (13.9-54.9)	90.5 (69.6-98.8)
	CADM1	3.9	68.2 (45.1-86.1)	33.3 (14.6-57.0)
	miR124-2/MAL	1.2/0.1	81.8 (59.7-94.8)	47.6 (25.7-70.2)
	miR124-2/CADM1	1.2/3.9	86.4 (65.1-97.1)	23.8 (8.2-47.2)
	MAL/CADM1	0.1/3.9	81.8 (59.7-94.8)	28.6 (11.3-52.2)
	miR124-2/MAL/CADM1	1.2/0.1/3.9	90.9 (70.8-98.9)	23.8 (8.2-47.2)

[a] Positive for at least one of the indicated host genes

Shading rows show markers or combination of markers with the best diagnostic performance and those with the maximal sensitivity for detection of HSIL+

For peer review only - http://bmjopen.bmj.com/site/about/guidelines.xhtml

Diagnostic performance of HPV genotyping combined with DNA methylation markers for the detection of HSIL or SCC

Diagnostic performance of HPV16 for detecting HSIL+ in clinician-collected samples showed - a sensitivity of 52.2% (95% CI 30.6-73.2) and specificity of 81.0% (95% CI 58.1-94.6) (Table 2). HPV16 and HPV18 combined showed a sensitivity of 60.9% (95% CI 38.5-80.3) and specificity of 81.0% (95% CI 58.1-94.6). A combination of *miR124-2/MAL* methylation markers and HPV16/18 genotyping showed a satisfactory diagnostic performance with a sensitivity 95.2% (95% CI 76.2-99.9) and specificity of 55% (95% CI 31.5-76.9). A combination of *MAL/CADM1* and HPV16/18 genotyping showed a maximal sensitivity of 100% (95% CI 85.9-100) at the expense of a specificity of 30% (11.9-54.3). Using HPV16/18/31/33/45/52/58 genotyping, either as a single marker or combined with different methylation markers, showed high sensitivities but at the expense of low specificities (Table 2).

For self-collected samples, detection of individual and combined HPV genotypes had the same diagnostic performance as seen in clinician-collected samples (Table 2). A combination of *miR124-2 or miR124/MAL and* HPV16/HPV18 showed similar sensitivity found for clinician-collected samples of 95.5% (95%CI:77.2-99.9), but reduced specificity (33.3%, 95%CI: 14.6-50.7). A combination of *miR124-2/CADM1* and HPV16/18 genotyping showed a maximal sensitivity of 100% (95% CI 84.6-100) and specificity of 23.8% (8.2-47.2). Adding HPV16/18/31/33/45/52/58 genotyping combined with different methylation markers showed high sensitivities at the expense of low specificities (Table 2).

For peer review only - http://bmjopen.bmj.com/site/about/guidelines.xhtml

363 ***Table 2.*** Performance of HPV genotyping combined with methylation markers for the detection of HSIL+

	Combined HPV/methylation detection for decision making				
**Sample type**	**HPV genotype [a]**	**Host Gene[b]**	**Percent methylation cut-off**	**Sensitivity % (95% CI)**	**Specificity (95%CI)**
Cervical, clinician-collected	16	NA	NA	52.2 (30.6-73.2)	81.0 (58.1-94.6)
	16,18	NA	NA	60.9 (38.5-80.3)	81.0 (58.1-94.6)
	16,18,31,33,45,52,58	NA	NA	95.7 (78.1-99.9)	33.3 (14.6-56.9)
	16,18	*miR124-2*	3.7	85.7 (63.7-97.0)	55.0 (31.5-76.9)
	16,18,31,33,45,52,58	*miR124-2*	3.7	95.2 (76-2-99.9)	25.0 (8.7-49.1)
	16,18	*MAL*	0.25	76.2 (52.8-91.8)	70.0 (45.7-88.1)
	16,18,31,33,45,52,58	*MAL*	0.25	100 (84.6-100)	14.3 (3.1-36.3)
	16,18	*CADM1*	3.5	90.5 (69.6-98.8)	35.0 (15.4-59.2)
	16,18,31,33,45,52,58	*CADM1*	3.5	95.2 (76.2-99.9)	20.0 (5.7-43.7)
	16,18	*miR124-2/MAL*	3.7/0.25	95.2 (76.2-99.9)	55.0 (31.5-76.9)
	16,18,31,33,45,52,58	*miR124-2/MAL*	3.7/0.25	100 (83.9-100)	25.0 (8.7-49.0)
	16,18	*miR124-2/CADM1*	3.7/3.5	95.2 (76.2-99.9)	25.0 (8.7-49.1)
	16,18,31,33,45,52,58	*miR124-2/CADM1*	3.7/3.5	95.2 (76.2-99.9)	15.0 (3.2-37.9)
	16,18	*MAL/CADM1*	0.25/3.7	100 (85.9-100)	30.0 (11.9-54.3)
	16,18,31,33,45,52,58	*MAL/CADM1*	0.25/3.7	100 (83.9-100)	20.0 (5.7-43.7)
Vaginal, self-collected	16	NA	NA	52.2 (30.6-73.2)	81.0 (58.1-94.6)
	16,18	NA	NA	60.9 (38.5-80.3)	81.0 (58.1-94.6)
	16,18,31,33,45,52,58	NA	NA	95.7 (78.1-99.9)	33.3 (14.6-56.9)
	16,18	*miR124-2*	1.2	95.5 (77.2-99.9)	33.3 (14.6-50.7)
	16,18,31,33,45,52,58	*miR124-2*	1.2	100 (84.6-100)	14.3 (3.1-36.3)
	16,18	*MAL*	0.1	68.2 (45.1-86.1)	71.4 (47.8-88.7)
	16,18,31,33,45,52,58	*MAL*	0.1	95.5 (77.2-99.9)	28.6 (11.3-52.2)
	16,18	*CADM1*	3.9	90.9 (70.8-98.9)	33.3 (14.6-56.9)

For peer review only - http://bmjopen.bmj.com/site/about/guidelines.xhtml

364

16,18,31,33,45,52,58	*CADM1*	3.9	100 (84.6-100)	9.5 (1.2-30.4)
16,18	*miR124-2/MAL*	1.2/0.1	95.5 (77.2-99.9)	33.3 (14.6-57.0)
16,18,31,33,45,52,58	*miR124-2/MAL*	1.2/0.1	100 (84.6-100)	14.3 (3.1-36.3)
16,18	*miR124-2/CADM1*	1.2/3.9	100 (84.6-100)	23.8 (8.2-47.2)
16,18,31,33,45,52,58	*miR124-2/CADM1*	1.2/3.9	100 (84.6-100)	4.8 (0.1-23.8)
16,18	*MAL/CADM1*	0.1/3.9	90.9 (70.8-98.9)	28.6 (11.3-52.2)
16,18,31,33,45,52,58	*MAL/CADM1*	0.1/3.9	100 (84.6-100)	9.5 (1.2-30.4)

365 [a] Detection of at least one of the indicated HPV genotypes

366

367 [b] Positive for at least one of the indicated host genes

368

369 Shading rows show markers or combination of markers with the best diagnostic performance and those with the maximal sensitivity for detection of HSIL+

For peer review only - http://bmjopen.bmj.com/site/about/guidelines.xhtml

For peer review only

Comparison of levels of methylation between paired self-collected and clinician-collected samples

There was generally a lower percentage of methylation for each marker in vaginal samples compared with their paired cervical samples (Figure 3). However, this only reached statistical significance for *miR124-2* in HSIL (p=0.0002) (Figure 3).

Agreement of paired self-collected and clinician-collected samples for *miR124-2*, *MAL* and *CADM1* methylation

Analysis was performed for 40 patients with paired, assessable samples. Initial analysis was performed without considering the underlying disease status. OPA, PPA and NPA were 62.5%, 77.3% and 44.4% respectively for *miR124-2* methylation, 77.5%, 46.2% and 92.6% respectively for *MAL* methylation, and 57.5%, 67.9% and 33.3% respectively for *CADM1*.

In analyses limited to HSIL+ paired clinician- and self-collected samples, the OPA, PPA and NPA were 75.0% (53.1-88.8), 81.3% (57.0-93.4) and 50.0% (15.0-85.0) respectively for *miR124-2* methylation, 65.0% (43.3-81.9), 45.5% (21.3-72.0) and 88.9% (56.5-98.0) respectively for *MAL* methylation; and 65.0% (43.3-81.9), 68.8% (44.4-85.8) and 50.0% (15.0-85.0) respectively for *CADM1*.

For peer review only - http://bmjopen.bmj.com/site/about/guidelines.xhtml

For peer review only

Discussion

In this study, we explored *CADM1*, *MAL* and *miR124-2* DNA methylation using clinician- and self-collected HPV+ samples from Papua New Guinea, a setting with a high burden of cervical disease. In clinician-collected cervical samples, DNA methylation of *MAL* and *miR124-2* were significantly higher in HSIL/cancer compared with normal/LSIL samples, while the methylation of *CADM1* failed to distinguish between cytology grades. Methylation of *miR124-2* showed the highest AUC to distinguish HSIL from normal/LSIL and the second-highest performance to predict SCC, with AUC values above 0.819. Exploration of diagnostic performance of *miR124-2* in combination with *MAL* for detection of HSIL+, showed an excellent performance (sensitivity 90.5%, specificity 70%). Similar findings were obtained with self-collected samples, though their performance was reduced. Overall, *miR124-2* in combination with *MAL* DNA methylation performed well for the detection of HSIL+ in women from LMIC. Addition of HPV16/18 or extensive genotyping improved sensitivity at the expense of lower specificity and higher referrals to immediate treatment. *miR124-2/MAL* methylation markers are suitable for further evaluation against histology end points, and subsequently in LMIC-based field studies for triage of hrHPV+ women to identify high-grade disease.

Increased levels of *miR124-2*, *CADM1* and *MAL* methylation according to lesion grade have been observed in studies performed in clinician-collected cervical samples from high-income populations (27-29). *miR124-2* methylation alone or in combination with *CADM1* and *MAL* has high sensitivity and specificity for the detection of CIN3+ in HPV+ clinician-collected cervical specimens in women from high-income countries (sensitivity 68.0–94.7%, specificity 50.7–78.9%) (30).

For peer review only - http://bmjopen.bmj.com/site/about/guidelines.xhtml

Few studies have been performed in LMIC using clinician-collected samples. An analysis of the same markers in HPV+ and HIV+ women from Kenya found the AUC of *miR124-2* methylation for CIN2+ and CIN3+ were 0.730 and 0.810 respectively (31), marginally lower to those obtained in the current exploratory study. However, these results are not directly comparable to ours as their lesion grade was based on histological diagnosis and HIV+ participants. In the current study, we were unable to stratify by HIV status, but assume a low HIV prevalence as the HIV prevalence in the Papua New Guinean general population is <1%. Two other studies performed in South Africa and Thailand (upper middle-income countries) also showed good performance of some of these markers, alone or in combination, for the detection of HSIL+ (32, 33).

CADM1 was not a good diagnostic marker, this is consistent with the findings of two other studies conducted in high-income countries (34, 35).

No research has been conducted looking methylation in self-collected samples from LMIC. Diagnostic performance of *miR124-2* for detection of HSIL+ in self-collected samples showed a sensitivity of 77.3% and specificity of 47.6%, while combining *miR124-2* with *MAL* increased the sensitivity to 81.8%, showing promise for self-sampling applications. While the sensitivity/specificity was slightly lower than clinician-collected samples, this may be offset by higher screening rates achieved by this more acceptable method of collection (11).

By comparison, studies of *miR124-2* and *MAL* single or in combination with other methylation markers for detection of CIN3+ in self-collected samples from high-income settings, have shown a wide range of sensitivities and specificities with values between 37.5–78.4% and

For peer review only - http://bmjopen.bmj.com/site/about/guidelines.xhtml

47.2–98% respectively (36-39). This wide variation may be explained by variable methodology [self-sampling collection devices, methylation detection assays, genes analysed, thresholds to define methylation positivity, study populations, disease end points (cytological HSIL or histological CIN2/3+), etc].

Triage combining HPV16/18 genotyping and methylation analysis has shown promise in clinician-collected samples in high-income settings (37, 38, 40, 41). Methylation analysis of *miR124-2/MAL* combined with HPV16/18 genotyping had a sensitivity for CIN3+ of 77.6% and specificity of 54.8% (40). A recent prospective cohort study in an upper middle-income country analysing methylation markers ZNF671/ASTN1/ITGA4/RXFP3/SOX17/DLX1 showed a sensitivity and specificity of 96.6% and 58.3% respectively to detect CIN2+ when combining methylation and HPV16/18 (42). In our study, combining HPV16/18 detection with *miR124-2/MAL* methylation improved sensitivity (increasing from 90.5 to 95.2%), but reduced specificity (decreasing from 70.0% to 55.0%). In self-collected samples the sensitivity was similar to clinician-collected samples (95.5%) but the specificity was lower (33.3%).

Combining extended genotyping (HPV16/18/31/33/45/52/58) with miR124-2/MAL methylation had similar sensitivity to *miR124/MAL/HPV16/18* testing, but in our setting showed lower specificity. Regardless of study population, HPV prevalence, sample type, marker panel and age, the combination of host cell DNA methylation analysis with partial or extended genotyping could serve as a potential triage strategy for hrHPV+ women, at the expense of a higher referral to treatment.

For peer review only - http://bmjopen.bmj.com/site/about/guidelines.xhtml

Self-collected samples generally had lower levels of methylation than paired clinician-collected samples, likely due to lower diseased/normal cell ratios. For example, values for the GynTect® assay (detecting six methylation markers) were significantly lower for self-collected than clinician-collected samples and the overall concordance was moderate (kappa 0.394) (43). Further discovery work to identify markers suitable for self-collection should consider using self-collected samples from LMIC. Verlaat et al., 2018 performed a chip-based genome DNA methylation screen on self-samples from a high-income country and identified a three-gene methylation classifier (ASCL1, LHX8 and ST6GALNAC5) that was able to identify CIN3 in cervical self-samples with a ROC of 0.88, which is superior to currently available methods (44).

Limitations of this study include the small sample size which led to broad confidence intervals; however, it aimed to provide estimated effects (point estimates with confidence intervals) for all measures of interest which are valuable for planning subsequent studies in LMIC. It was not feasible to conduct cervical biopsies in this setting due to limited specialist staff and infrastructure; hence LBC HSIL+ was the diagnostic reference standard rather than a histological end point and this may have impacted estimates of sensitivity/specificity for methylation markers. Since HPV testing is more sensitive than cytology at detecting HSIL+, then the selection of cytology as a reference standard will result in an overestimation of the sensitivity of the HPV test and decrease in the specificity, compared to results using histology as a reference standard". However, to avoid disease misclassification by cytology all slides were assessed by two independent experienced cytologists, and dual p16/Ki-67 immunostaining was performed to resolve disagreements. A triage test based on methylation markers could be highly suited to regions without access to colposcopy, where the combination of HPV detection, methylation analysis and cytology could confirm HSIL+ in the

For peer review only - http://bmjopen.bmj.com/site/about/guidelines.xhtml

absence of colposcopy examination. Additional information is required to understand the long-term safety of methylation markers in screening programs, including longitudinal data from a low-resource setting and analysis of methylation in self-collected samples from general population and HIV+ women.

Conclusions

DNA methylation of *miR124-2/MAL* alone or in combination with HPV16/18 is very promising as a triage test for the prediction of HSIL/SCC among women with oncogenic HPV infections. Self-collected samples showed reduced specificity compared to clinician-collected samples, indicating a need to identify additional biomarkers for this sample type. Future evaluation of these results against histological end points, and subsequently in different populations may lead to better tests to manage HPV+ women in LMIC.

Acknowledgements

We thank the women who participated in this research and their families and community, without whom this work would not have been possible. Special thanks to the Papua New Guinea study team who collaborated to the fieldwork, and Cepheid (Sunnyvale, CA, USA) for donating Xpert HPV cartridges.

Authors' Contributions

For peer review only - http://bmjopen.bmj.com/site/about/guidelines.xhtml

MM: Conceptualization, methodology, laboratory work, writing original draft, software analysis. DAM. Supervision, methodology, investigation, writing–review and editing. GT: Coordinated the evaluation of liquid-based cytology specimens, review and editing. SMG: Conceived the original trial, supervision, funding acquisition, project administration, writing—review and editing. SP: Software analysis, data curation, review and editing. PB, DH, PJT: Investigation, laboratory work, review and editing. SGB: Conceived the original trial, coordinated clinical implementation, review and editing. JB, JG, ZK, GMM: coordinated clinical implementation in the original trial, review and editing. AC, GH investigation, review and editing. JMB, MS, JMK: Conceived the original trial, funding acquisition, review and editing. AJV: Conceived the original trial, conceptualization, funding acquisition, project administration, writing, review and editing GM: Conceptualization, supervision, funding acquisition, investigation, project administration, writing, review and editing.

For peer review only

References

1. Sung H, Ferlay J, Siegel RL, et al. Global cancer statistics 2020: GLOBOCAN estimates of incidence and mortality worldwide for 36 cancers in 185 countries. CA Cancer J Clin. 2021;71(3):209-249.

2. Lemp JM, De Neve JW, Bussmann H, et al. Lifetime prevalence of cervical cancer screening in 55 low- and middle-income countries. JAMA. 2020;324:1532-1542.

3. Ferlay J, Ervik M, Lam F, et al. Global Cancer Observatory: Cancer Today. Lyon, France: International Agency for Research on Cancer. Available from: https://gco.iarc.fr/today, accessed 20 07 2021.

4. PATH. Global HPV Vaccine Introduction Overview: projected and current national introductions, demonstration/pilot projects, gender-neutral vaccination programs, and global HPV vaccine introduction maps (2006-2023). PATH; 2020. Accessed November 23, 2020. Path.org/resources/global-hpv-vaccine-introduction-overview/ 179.

5. Simelela PN. WHO global strategy to eliminate cervical cancer as a public health problem: An opportunity to make it a disease of the past. Int J Gynaecol Obstet. 2021;152(1):1-3.

6. WHO. WHO guideline for screening and treatment of cervical pre-cancer lesions for cervical cancer prevention, second edition. Geneva: World Health Organization; 2021

7. Toliman P, Badman SG, Gabuzzi J, et al. Field Evaluation of Xpert HPV Point-of-Care Test for Detection of Human Papillomavirus Infection by Use of Self-Collected Vaginal and Clinician-Collected Cervical Specimens. J Clin Microbiol. 2016;54(7):1734-7.

8. Toliman PJ, Kaldor JM, Badman SG, et al. Evaluation of self-collected vaginal specimens for the detection of high-risk human papillomavirus infection and the prediction of high-grade

For peer review only

cervical intraepithelial lesions in a high-burden, low-resource setting. Clin Microbiol Infect. 2019;25:496-503.

9. Arbyn M, Smith SB, Temin S, et al. Detecting cervical precancer and reaching under screened women by using HPV testing on self samples: updated meta-analyses. BMJ (Clinical research ed) 2018; 363: k4823.

10. De Pauw H, Donders G, Weyers S, et al. Cervical cancer screening using HPV tests on self-samples: attitudes and preferences of women participating in the VALHUDES study. Arch Public Health. 2021;79(1):155.

11. Hawkes D, Keung MHT, Huang Y, et al. Self-Collection for Cervical Screening Programs: From Research to Reality. Cancers (Basel). 2020;12(4):1053.

12. Murillo R, Gamboa O, Hernández G, et al. Accuracy of combined molecular and morphology-based triage for HPV-positive women in routine cervical cancer screening services from Colombia. Prev Med. 2021;153:106801.

13. Mirabello L, Sun C, Ghosh A, et al. Methylation of human papillomavirus type 16 genome and risk of cervical precancer in a Costa Rican population. J. Natl Cancer Inst. 2012;104:556–565.

14. De Strooper LM, van Zummeren M, Steenbergen RD, . CADM1, MAL and miR124-2 methylation analysis in cervical scrapes to detect cervical and endometrial cancer. J Clin Pathol. 2014; 67(12): 1067-71.

15. Cook D A, Krajden M, Brentnall A R, et al. Evaluation of a validated methylation triage signature for human papillomavirus positive women in the HPV FOCAL cervical cancer screening trial. Int J Cancer. 2018; 144: 2587–2595.

For peer review only - http://bmjopen.bmj.com/site/about/guidelines.xhtml

16. Bonde J, Floore A, Ejegod D, et al. Methylation markers FAM19A4 and miR124-2 as triage strategy for primary human papillomavirus screen positive women: A large European multicenter study. Int J Cancer. 2021;148(2):396-405.

17. Verhoef VMJ, van Kemenade FJ, Rozendaal L, et al. Follow-up of high-risk HPV positive women by combined cytology and bi-marker CADM1/MAL methylation analysis on cervical scrapes. Gynecol Oncol. 2015;137(1):55–59.

18. Zhang L, Tan W, Yang H, et al. Detection of Host Cell Gene/HPV DNA Methylation Markers: A Promising Triage Approach for Cervical Cancer. Front Oncol. 2022;12:831949.

19. Vallely AJ, Saville M, Badman SG, et al. Point-of-care HPV-DNA testing of self-collected specimens and same-day thermal ablation for the early detection and treatment of cervical pre-cancer in high-burden, low-resource settings: Results of a prospective intervention trial in Papua New Guinea (HPV-STAT). Lancet Glob Health. 2022 Sep;10(9):e1336-e1346.

20. Li D, Xie Z, Pape ML, Dye T. An evaluation of statistical methods for DNA methylation microarray data analysis. BMC Bioinformatics. 2015 Jul 10;16:217.

21. Phillips S, Cassells K, Garland SM, et al. Gene methylation of CADM1 and MAL identified as a biomarker of high grade anal intraepithelial neoplasia. Sci Rep. 2022;12(1):3565.

22. Hesselink AT, Heideman DA, Steenbergen RD, et al. Methylation marker analysis of self-sampled cervico-vaginal lavage specimens to triage high-risk HPV-positive women for colposcopy. Int J Cancer. 2014;135(4):880-6.

23. Unal I. Defining an Optimal Cut-Point Value in ROC Analysis: An Alternative Approach. Comput Math Methods Med. 2017;2017:3762651.

24.Thiele C & Hirschfeld G. Cutpointr: Improved estimation and validation of optimal cutpoints in R. J. Stat. Softw. 2021;98(11): 1–27.

For peer review only - http://bmjopen.bmj.com/site/about/guidelines.xhtml

25. Food, U. S. “Drug Administration. Statistical Guidance on Reporting Results from Studies Evaluating Diagnostic Tests-Guidance for Industry and FDA Staff.” (2007).

26. Bossuyt PM, Reitsma JB, Bruns DE, et al. STARD 2015: An Updated List of Essential Items for Reporting Diagnostic Accuracy Studies.

27. Hesselink AT, Heideman DA, Steenbergen RD, et al. Combined promoter methylation analysis of CADM1 and MAL: an objective triage tool for high-risk human papillomavirus DNA-positive women. Clin Cancer Res. 2011;17:2459–2465.

28. Bierkens M, Hesselink AT, Meijer CJ, et al. CADM1 and MAL promoter methylation levels in hrHPV-positive cervical scrapes increase proportional to degree and duration of underlying cervical disease. Int J Cancer. 2013;133:1293–1299

29. Clarke MA, Luhn P, Gage JC, et al. Discovery and validation of candidate host DNA methylation markers for detection of cervical precancer and cancer. Int J Cancer. 2017;141(4):701-710

30. Kremer WW, Steenbergen R, Heideman D, et al. The use of host cell DNA methylation analysis in the detection and management of women with advanced cervical intraepithelial neoplasia: a review. BJOG. 2021;128(3):504-514.

31. De Vuyst H, Franceschi S, Plummer M, et al. Methylation levels of CADM1, MAL, and MIR124-2 in cervical scrapes for triage of HIV-infected, high-risk HPV-positive women in Kenya. J Acquir Immune Defic Syndr. 2015;70:311–8.

32. Van Zummeren M, Kremer WW, Van Aardt MC, et al. Selection of women at risk for cervical cancer in an HIV-infected South African population. AIDS. 2017;31:1945–53.

For peer review only

For peer review only - http://bmjopen.bmj.com/site/about/guidelines.xhtml

33. Dankai W, Khunamornpong S, Siriaunkgul S, et al. Role of genomic DNA methylation in detection of cytologic and histologic abnormalities in high risk HPV infected women. PloS One. 2019;14(1):e0210289

34. Vasiljevic N, Scibior-Bentkowska D, Brentnall AR, et al. Credentialing of DNA methylation assays for human genes as diagnostic biomarkers of cervical intraepithelial neoplasia in high-risk HPV positive women. Gynecol Oncol. 2014;132:709–14.

35. Salta S, Maia-Moço L, Estev~aoPereira H, et al. Performance of DNA methylation-based biomarkers in the cervical cancer screening program of northern Portugal: A feasibility study. Int J Cancer. 2021;149(11):1916-1925.

36. Verhoef VMJ, Bosgraaf RP, van Kemenade FJ, et al. Triage by methylation-marker testing versus cytology in women who test HPV-positive on self-collected cervicovaginal specimens (PROHTECT-3): a randomised controlled non-inferiority trial. Lancet Oncol. 2014;15(3):315–322.

37. De Strooper LMA, Verhoef VMJ, Berkhof J, et al. Validation of the FAM19A4/mir124-2 DNA methylation test for both lavage- and brush-based self-samples to detect cervical (pre)cancer in HPV positive. Gynecol Oncol. 2016;141:341–347.

38. Luttmer R, De Strooper LM, Dijkstra MG, et al. FAM19A4 methylation analysis in self samples compared with cervical scrapes for detecting cervical (pre)cancer in HPV-positive women. Br J Cancer. 2016;115:579–87

39. Del Mistro A, Frayle H, Rizzi M, et al. Methylation analysis and HPV genotyping of self-collected cervical samples from women not responding to screening invitation and review of the literature. PloS ONE. 2017;12(3):e0172226

For peer review only - http://bmjopen.bmj.com/site/about/guidelines.xhtml

40. Verhoef VMJ, Heideman DAM, van Kemenade FJ, et al. Methylation marker analysis and HPV16/18 genotyping in high-risk HPV positive self-sampled specimens to identify women with high grade CIN or cervical cancer. Gynecol Oncol. 2014;135(1):58–63.

41. Leeman A, Del Pino M, Marimon L, et al. Reliable Identification of Women With CIN3+ Using hrHPV Genotyping and Methylation Markers in a Cytology-Screened Referral Population. Int J Cancer. 2019; 144:160–8.

42. Zhang L, Zhao X, Hu S, et al. Triage performance and predictive value of the human gene methylation panel among women positive on self-collected HPV test: Results from a prospective cohort study. Int J Cancer. 2022;151(6):878-887

43. Klischke L, von Ehr J, Kohls F, et al. Performance of a six-methylation-marker assay on self-collected cervical samples – A feasibility study. J Virol Methods. 2021;295:114219.

44. Verlaat W, Snoek BC, Heideman DAM, et al. Identification and Validation of a 3-Gene Methylation Classifier for HPV-Based Cervical Screening on Self-Samples. Clin Cancer Res. 2018;24(14):3456-3464.

For peer review only - http://bmjopen.bmj.com/site/about/guidelines.xhtml

Figure 1. Percentage of DNA methylation of gene *CADM1, MAL* and *miR124-2* according to cytological grade in oncogenic HPV positive clinician-collected cervical samples (Cervical) in the top panel and self-collected vaginal samples (Vaginal) in the bottom panel. Whiskers correspond to the first and third quartiles (the 25th and 75th percentiles). Overall significance by Kruskal-Wallis is indicated.

For peer review only

For peer review only - http://bmjopen.bmj.com/site/about/guidelines.xhtml

Figure 2. Receiver operating characteristic (ROC) curve values of the performance of DNA methylation of *CADM1, MAL* and *miR124-2* for distinguishing HSIL from LSIL/normal, and SCC from LSIL/normal, stratified by type; clinician-collected cervical samples (A and C) or self-collected vaginal samples (B and D).

For peer review only

Figure 3. Percentage methylation of gene *CADM1, MAL* and *miR124-2* comparing paired clinician-collected cervical samples (Cervical) and self-collected vaginal samples (Vaginal). Comparisons assessed by Wilcoxon signed rank test, with whiskers corresponding to the first and third quartiles (the 25th and 75th percentiles).

For peer review only

For peer review only - http://bmjopen.bmj.com/site/about/guidelines.xhtml

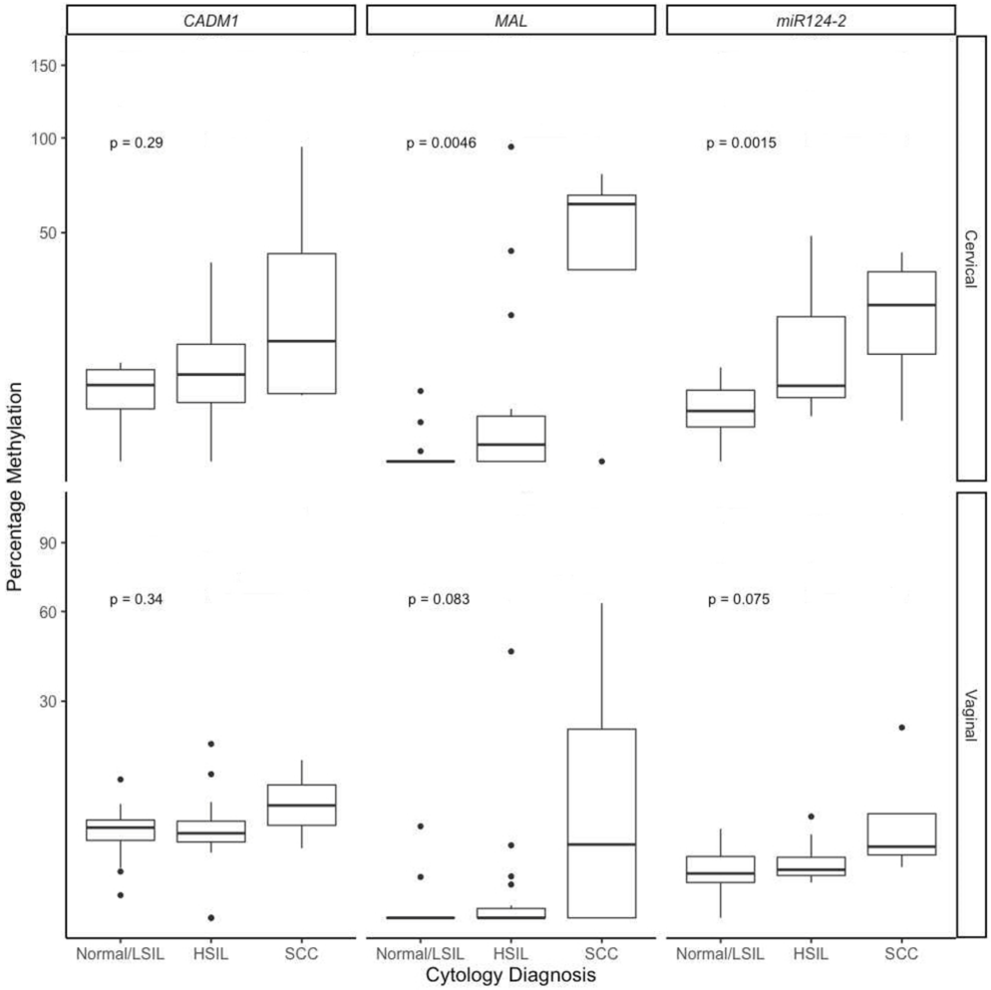

Figure 1. Percentage of DNA methylation of gene CADM1, MAL and miR124-2 according to cytological grade in oncogenic HPV positive clinician-collected cervical samples (Cervical) in the top panel and self-collected vaginal samples (Vaginal) in the bottom panel. Whiskers correspond to the first and third quartiles (the 25th and 75th percentiles). Overall significance by Kruskal-Wallis is indicated.

190x190mm (133 x 133 DPI)

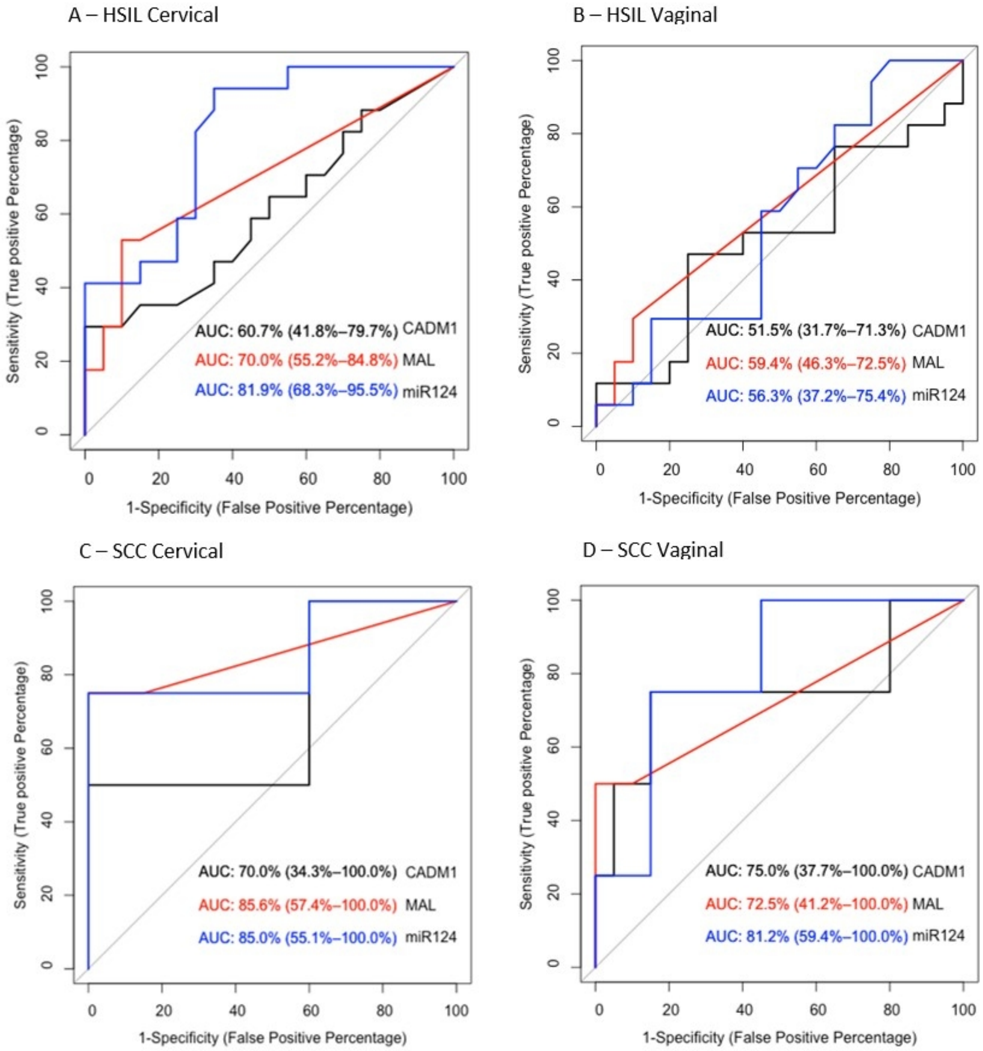

Figure 2. Receiver operating characteristic (ROC) curve values of the performance of DNA methylation of CADM1, MAL and miR124-2 for distinguishing HSIL from LSIL/normal, and SCC from LSIL/normal, stratified by type; clinician-collected cervical samples (A and C) or self-collected vaginal samples (B and D).

146x155mm (144 x 144 DPI)

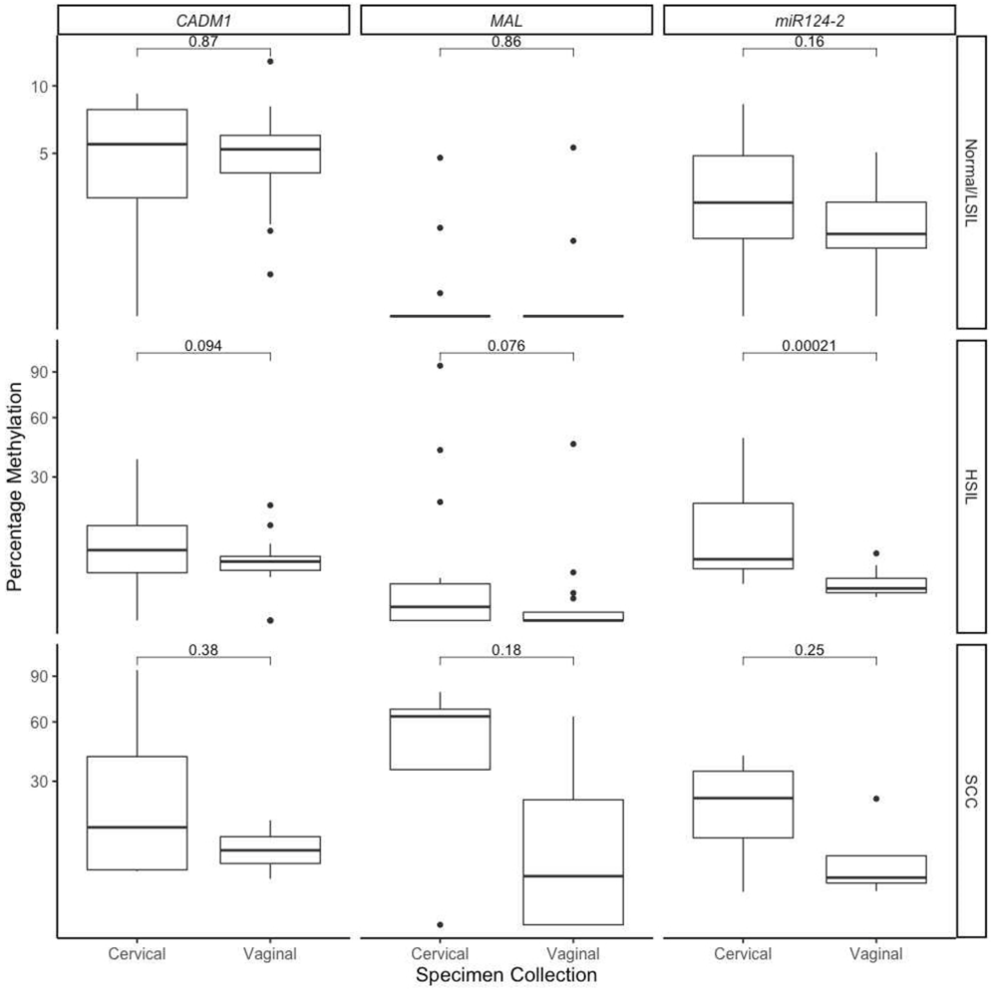

Figure 3. Percentage methylation of gene CADM1, MAL and miR124-2 comparing paired clinician-collected cervical samples (Cervical) and self-collected vaginal samples (Vaginal). Comparisons assessed by Wilcoxon signed rank test, with whiskers corresponding to the first and third quartiles (the 25th and 75th percentiles).

190x190mm (133 x 133 DPI)

Reporting checklist for diagnostic test accuracy study.

Based on the STARD guidelines.

Instructions to authors

Complete this checklist by entering the page numbers from your manuscript where readers will find each of the items listed below.

Your article may not currently address all the items on the checklist. Please modify your text to include the missing information. If you are certain that an item does not apply, please write "n/a" and provide a short explanation.

Upload your completed checklist as an extra file when you submit to a journal.

In your methods section, say that you used the STARDreporting guidelines, and cite them as:

Bossuyt PM, Reitsma JB, Bruns DE, Gatsonis CA, Glasziou PP, Irwig L, LijmerJG Moher D, Rennie D, de Vet HCW, Kressel HY, Rifai N, Golub RM, Altman DG, Hooft L, Korevaar DA, Cohen JF, For the STARD Group. STARD 2015: An Updated List of Essential Items for Reporting Diagnostic Accuracy Studies.

		Reporting Item	Page Number
**Title or abstract**			
None	#1	Identification as a study of diagnostic accuracy using at least one measure of accuracy (such	3

For peer review only

		as sensitivity, specificity, predictive values, or AUC)	

Abstract

None	#2	Structured summary of study design, methods, results, and conclusions (for specific guidance, see STARD for Abstracts https://www.equator-network.org/reporting-guidelines/stard-abstracts/)	3

Introduction

None	#3	Scientific and clinical background, including the intended use and clinical role of the index test	6-7
None	#4	Study objectives and hypotheses	8

Methods

Study design	#5	Whether data collection was planned before the index test and reference standard were performed (prospective study) or after (retrospective study)	9-10
Participants	#6	Eligibility criteria	9-10

For peer review only

Participants	#7	On what basis potentially eligible participants were identified (such as symptoms, results from previous tests, inclusion in registry)	9-10
Participants	#8	Where and when potentially eligible participants were identified (setting, location and dates)	9,11
Participants	#9	Whether participants formed a consecutive, random or convenience series	9,11
Test methods	#10	Index and reference tests in sufficient detail to allow replication	9-13
Test methods	#11	Rationale for choosing the reference standard (if alternatives exist)	10
Test methods	#12	Definition of and rationale for test positivity cut-offs or result categories of the index and reference tests, distinguishing pre-specified from exploratory	10,12-13
Test methods	#13	Whether clinical information and reference standard results were available to the performers / readers of the index test; Whether clinical information and index test results were available to the assessors of the reference standard	11

For peer review only

Analysis	#14	Methods for estimating or comparing measures of diagnostic accuracy	12-13
Analysis	#15	How indeterminate index test or reference standard results were handled	12-13
Analysis	#16	How missing data on the index test and reference standard were handled	12
Analysis	#17	Any analyses of variability in diagnostic accuracy, distinguishing pre-specified from exploratory	10,13
Analysis	#18	Intended sample size and how it was determined	9,11
**Results**			
Participants	#19	Flow of participants, using a diagram	NA This is an exploratory study. The flow of participants was outlined in detail in the results section, and thus a diagram was not deemed necessary.
Participants	#20	Baseline demographic and clinical characteristics of participants	15

For peer review only

Participants	#21	Distribution of severity of disease in those with the target condition, and distribution of alternative diagnoses in those without the target condition	15
Participants	#22	Time interval and any clinical interventions between index test and reference standard	11,15
Test results	#23	Cross tabulation of the index test results (or their distribution) by the results of the reference standard	15,16,22
Test results	#24	Estimates of diagnostic accuracy and their precision (such as 95% confidence intervals)	16-21
Test results	#25	Any adverse events from performing the index test or the reference standard	15
**Discussion**			
None	#26	Study limitations, including sources of potential bias, statistical uncertainty, and generalisability	26
None	#27	Implications for practice, including the intended use and clinical role of the index test	26-27
**Other information**			
None	#28	Registration number and name of registry	9

For peer review only

None	#29	Where the full study protocol can be accessed	9
None	#30	Sources of funding and other support; role of funders	2

The STARD checklist is distributed under the terms of the Creative Commons Attribution License CC-BY. This checklist was completed on 10. October 2023 using https://www.goodreports.org/, a tool made by the EQUATOR Network in collaboration with Penelope.ai

For peer review only
